# Selective Synthesis of *N*-[1,3,5]Triazinyl-α-Ketoamides and *N*-[1,3,5]Triazinyl-Amides from the Reactions of 2-Amine-[1,3,5]Triazines with Ketones

**DOI:** 10.3390/molecules28114338

**Published:** 2023-05-25

**Authors:** Yue Li, Pengzhen Zhong, Junna Zhao, Zexi Pan, Chen Zhang, Dongmei Cui

**Affiliations:** 1 College of Pharmaceutical Science, Zhejiang University of Technology, Hangzhou 310014, China; li__yue@163.com (Y.L.); zhongpengzhenn@163.com (P.Z.); zhaojunna9@163.com (J.Z.); 2111907117@zjut.edu.cn (Z.P.); 2 School of Pharmaceutical Sciences, Zhejiang University, Hangzhou 310058, China

**Keywords:** amino [1,3,5]triazines, ketones, α-ketoamides, amides, oxidation

## Abstract

In this study, we report a selective approach for synthesizing *N*-([1,3,5]triazine-2-yl) α-ketoamides and *N*-([1,3,5]triazine-2-yl) amides from ketones with 2-amino[1,3,5]triazines through oxidation and oxidative C−C bond cleavage reaction, respectively. The transformation proceeds under mild conditions, provides good functional group tolerance and chemoselectivity, and will serve as a valuable tool for the synthesis of bioactive products.

## 1. Introduction

**Scheme 1:**
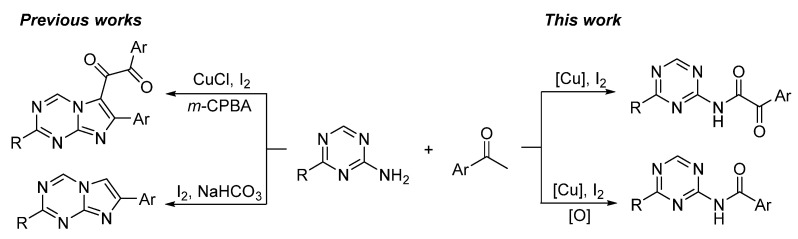
The reaction of 2-amino[1,3,5]triazines and ketones.

The 1,3,5-triazines are ubiquitous structural motifs in pharmaceutically active molecules and have a wide array of biological activities, including antibacterial, anti-HSV-1, anticancer, and anti-HIV biological activities [1,2,3]. Therefore, the development of novel and practical methods for the construction of functionalized 1,3,5-triazines is highly desirable. The α-ketoamides and amides are important intermediates in organic synthesis and are also building blocks in many natural products and pharmaceuticals [4,5,6,7,8,9,10,11,12]. Due to their wide utilities, the synthesis of these compounds has attracted considerable interest in recent years. Traditionally, α-ketoamides and amides have been synthesized mainly by the amidation of carboxylic acid derivatives, such as acids and acyl halides. However, this conventional method requires harsh reaction conditions, harmful reagents, and the generation of waste, limiting their practical applicability. Recently, the direct synthesis of α-ketoamides and amides from ketones via oxidation or oxidative C−C bond cleavage has been reported; however, the utility of these methods is restricted owing to the limited availability of the starting materials and other reasons [13,14,15,16,17,18,19,20,21,22,23,24]. Very recently, we developed a new protocol for the synthesis of imidazo [1,2-a][1,3,5]triazines by using the reaction of 2-amino[1,3,5]triazines and ketones (Scheme 1) [25]. As part of our continued research on 2-amino[1,3,5]triazines, herein we report an efficient method for the selective construction of *N*-([1,3,5]triazine-2-yl) α-ketoamide and *N*-([1,3,5]triazine-2-yl) amide derivatives from 2-amino[1,3,5]triazines and ketones [26,27,28].

## 2. Results

In a recent paper, we reported the annulation of 2-amino-[1,3,5]triazines and ketones to construct imidazo [1,2-a][1,3,5]triazines. However, trace unexpected α-ketoamides **3** and amides **4** were found as byproducts. Interestingly, α-ketoamides **3a** became the favored product using CuCl (20 mol%) along with iodine (2 eq.) in DMSO at 120 °C for 1.5 h under a nitrogen atmosphere (90%) (Table 1, entry 1). Solvent screening studies revealed that PhCl, 1,2-dichlorobenzene (1,2-DCB), toluene, DMF, and dioxane furnished only a trace amount of the product **3a** (Table 1, entries 2–6). Increasing and reducing the temperature or time lowered the yields (Table 1, entries 7–10). Only a trace of the product **3a** was observed for long time (13 h), and the reaction became complex. Different copper salts were examined, but the yield did not improve (Table 1, entries 11–14). We also observed that the yield of the product was lower when CuCl, I_2_ or **2a** was reduced (Table 1, entries 15–17). Notably, **3a** was afforded in 75% or 48% yield when running the reaction under air or O_2_ (Table 1, entries 18–19).

**Scheme 2:**
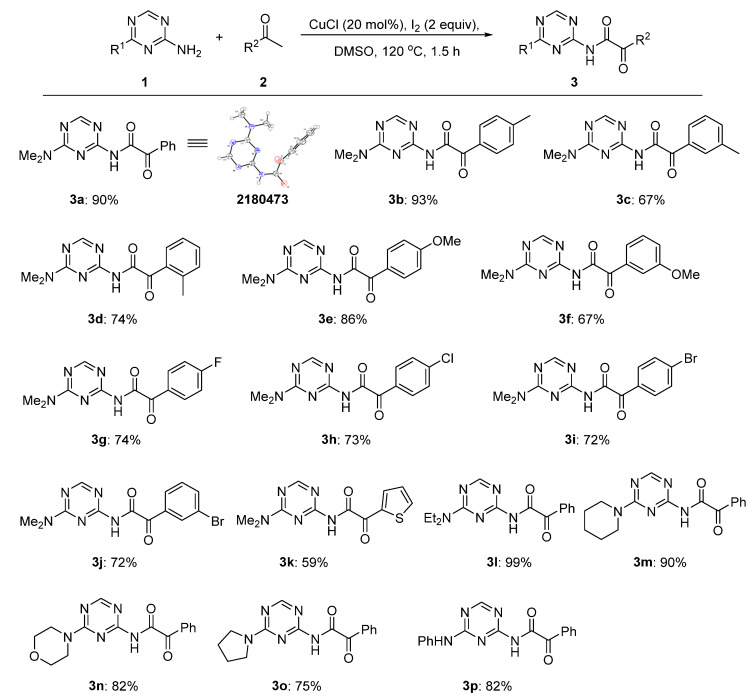
Substrate scope for synthesis of α-ketoamides **3**. Reaction Conditions: All reactions were performed with **1** (0.5 mmol), **2** (1.1 mmol), I_2_ (2.0 eq.), and CuCl (20 mol%) in DMSO (4 mL) for 1.5 h at 120 °C under N_2_; isolated yields.

Having established the optimal conditions, the substrate scope was examined (Scheme 2). Various electron-donating substituents on the aryl unit of ketones were investigated and furnished the respective products in 67–93% yields (**3b**–**f**). In addition, aryl ketones with electron-withdrawing substituents also work well in the reaction. For example, aryl ketones substituted with halogens at either the meta or para positions successfully underwent amidation to give α-ketoamides **3g**–**j** in good yields. In addition, thiophone was found to be suitable reaction partner and afforded the desired product **3k** with a 59% yield. Next, we surveyed the scope of 2-amino[1,3,5]triazine derivatives with different substituents on the C4-position. The derivatives with *N*,*N*-diethylamino, morpholino, pyrrolidino, and piperidino substituents were also competent starting materials for this strategy and performed the corresponding products **3l**–**o** in 75–99% yields. 2-Amine-[1,3,5]triazines with phenylamino substituent at the C4-position were also tolerated under the standard reaction conditions, giving the desired product **3p** with an 82% yield. Additionally, the structure of **3a** was unambiguously confirmed using the single crystal X-ray analysis (CCDC 2180473).

After demonstrating the reaction scope for the synthesis of *N*-([1,3,5]triazine-2-yl) α-ketoamides, we decided to pursue an oxidative C−C bond cleavage approach with *N*-([1,3,5]triazine-2-yl) amides formation. Using the above reaction conditions, we failed to obtain the desired product **4a** (Table 2, entry 1). The use of other copper salts such as CuI, CuBr, and Cu(OAc)_2_ was also found ineffective for the reaction (Table 2, entries 2–5). Employing CuCl_2_ along with 1,2-DCB or Diethylene glycol dimethyl ether (diglyme) solvent afforded the desired product **4a** with 27% and 11% yields, respectively (Table 2, entries 6–7). However, the other solvent (1,2,4-trichlorobenzene (1,2,4-TCB), DMF, NMP, and toluene) proved to be unproductive for the reaction (Table 2, entries 8–11). Increasing the amount of CuCl_2_ to 40 mol% and the temperature to 140 °C in the mixture of 1,2-DCB and diglyme afforded product **4a** with a 63% yield (Table 2, entry 13). The amount of I_2_ was screened, and the results showed that 1.5 equiv of I_2_ was optimal for this reaction (Table 2, entry 15). Stirring the reaction for a longer time and a shorter time gave product **4a** in lower yields (Table 2, entries 18–19). The reaction did not proceed either in the absence of copper salts and I_2_, and control experiments showed that oxygen was needed to perform the reaction (Table 2, entries 20–23).

**Scheme 3:**
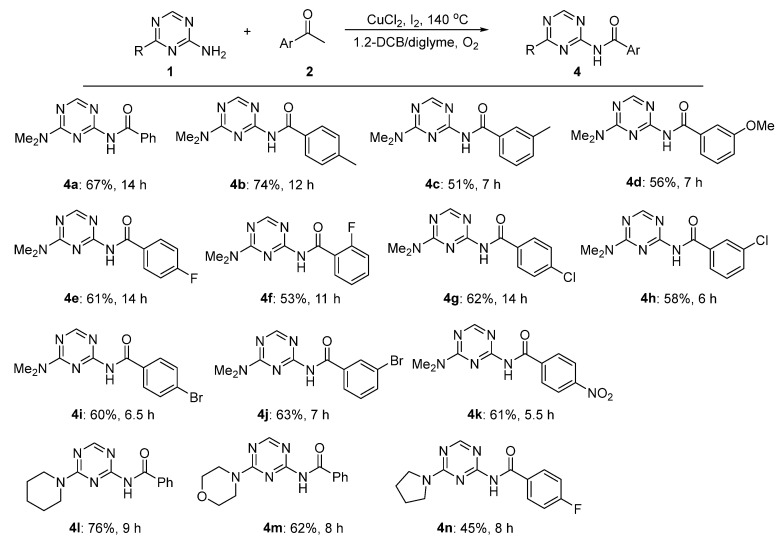
Substrate scope for synthesis of amides **4**. Reaction Conditions: All reactions were performed with **1** (0.5 mmol), **2** (1.1 mmol), I_2_ (0.75 eq.), and CuCl_2_ (40 mol%) in 1,2-DCB (2 mL) and diglyme (1 mL) at 140 °C oil bath under O_2_; isolated yields.

With a set of optimized conditions for the construction of *N*-([1,3,5]triazine-2-yl) amides in hand, the generality and scope of this protocol were explored (Scheme 3). Methyl and methoxy substituted aryl ketones reacted smoothly under the standard reaction conditions to deliver the desired products **4b**–**d**. Likewise, aryl ketones with electron-withdrawing groups such as F, Cl, and Br illustrated similar reactivity and afforded the corresponding amides **4e**–**j** with 53–63% yields. Regarding the scope of 2-amine-[1,3,5]triazines, the reaction proceeded well for substrates with morpholino, pyrrolidino, and piperidino groups.

**Scheme 4:**
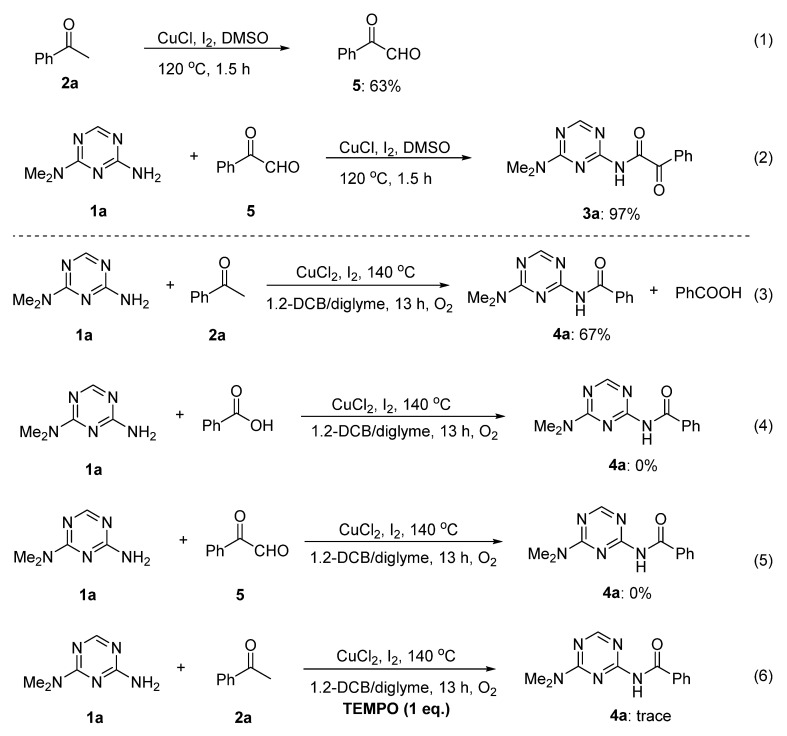
Control experiments.

To probe the reaction mechanistic path, a series of control experiments were further conducted (Scheme 4). For α-ketoamidation reaction, treatment of **2a** in the absence of **1a** under the standard condition gave phenyl glyoxal (**5**) (63%), and then **5** treated with **1a** to isolate the desired product **3a** with a 97% yield, indicating that **5** should be an intermediate for the formation of **3a** (Equations (1) and (2)). For the amidation reaction, first, when the reaction of **1a** with **2a** was carried out under the standard conditions, the trace of benzoic acid could be detected using ^1^H NMR (Equation (3)). However, no desired product **4a** was obtained from benzoic acid with **1a**, indicating that it is a byproduct for the formation of amides (Equation (4)). Next, the reaction of **5** that is potentially generated with **1a** did not give the amide **4a**, indicated that this compound was not the intermediate (Equation (5)). Furthermore, the reaction failed to furnish desired amide **4a** using TEMPO as a radical scavenger, suggesting that a radical pathway might be involved in this C–C single cleavage reaction (Equation (6)).

**Scheme 5:**
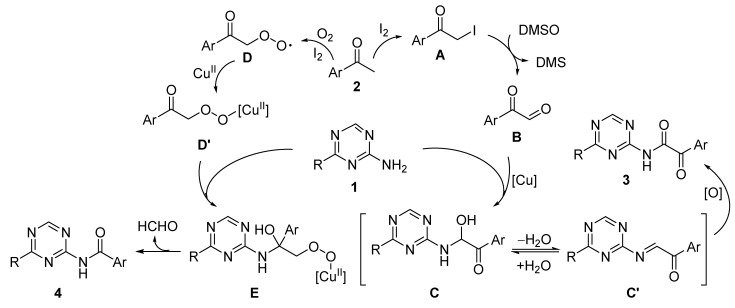
Proposed mechanism.

On the basis of our study and related reports, a plausible mechanism is depicted (Scheme 5) [14,17,20,23,29,30]. Initially, acetophenone **2** with I_2_ produced intermediate **A**, followed by the oxidation to produce intermediate **B**. The latter then reacted with 2-amino[1,3,5]triazine **1** to furnish intermediate **C** or **C′**, followed by further rapid oxidation to give **3** under the reaction conditions. On the other hand, ketone **2** was oxidized in the presence of copper, I_2_, and oxygen to generate superoxide intermediate **D′** via intermediate **D**. Then, the nucleophilic attack of 2-amine-[1,3,5]triazines to intermediate **D′** occurred to produce intermediate **E**. Subsequently, the cleavage of the C–C bond occurred during the rearrangement of intermediate **E**, leading to amide **4** and the aldehyde byproduct.

## 3. Materials and Methods

### 3.1. General Experiment

Under otherwise noted, materials were obtained from commercial suppliers and used without further purification. Thin layer chromatography (TLC) was performed using silica gel 60 F254 and visualized using UV light. Column chromatography was performed with silica gel (mesh 300–400). ^1^H NMR and ^13^C NMR spectra were recorded on a Bruker Avance 500 MHz spectrometer in CDCl_3_ with Me_4_Si as an internal standard. Data were reported as follows: chemical shift in parts per million (*δ*), multiplicity (s = singlet, d = doublet, t = triplet, q = quartet, br = broad, and m = multiplet), coupling constant in Hertz (Hz) and integration. IR spectra were recorded on an FT-IR spectrometer, and only major peaks are reported in cm^−1^. HRMS and mass data were recorded via ESI on a TOF mass spectrometer.

### 3.2. General Procedure for the Synthesis of N-([1,3,5]Triazine-2-yl) α-Ketoamides 3

A mixture of 1,3,5-triazine (0.5 mmol), ketone (1.1 mmol), CuCl (0.10 mmol) and I_2_ (1.00 mmol) was added in DMSO (4 mL). The resulting mixture was then stirred at 120 °C under N_2_. After the completion of the reaction, the reaction mixture was cooled to room temperature, 10% Na_2_S_2_O_3_ was added and the mixture was extracted with ethyl acetate (4 × 20 mL). The organic phase was dried over anhydrous Na_2_SO_4_ and concentrated in vacuo. The residue was purified using flash chromatography with petroleum ether and ethyl acetate as the eluent to give the pure product.

*N-(4-(dimethylamino)-1,3,5-triazin-2-yl)-2-oxo-2-phenylacetamide* (**3a**), White solid; mp 222–223 °C; ^1^H NMR (500 MHz, CDCl_3_) *δ* 11.66 (br, 1H), 8.51 (s, 1H), 8.10–7.94 (m, 2H), 7.67–7.60 (m, 1H), 7.51 (t, *J* = 7.4 Hz, 2H), 3.08 (s, 3H), 2.52 (s, 3H); ^13^C NMR (125 MHz, CDCl_3_) *δ* 187.5, 165.9, 163.6, 161.4, 134.3, 133.1, 129.8, 128.8, 36.7, 36.0; IR (KBr, cm^−1^): 3413, 2873, 1682, 1609, 1545, 1413, 1357, 1215, 1176, 993, 725, 597. HRMS (ESI) *m*/*z* [M + H]^+^ calcd for C_13_H_14_N_5_O_2_ 272.1147, found 272.1148

*N-(4-(dimethylamino)-1,3,5-triazin-2-yl)-2-oxo-2-(p-tolyl)acetamide* (**3b**), White solid; mp 219–221 °C; ^1^H NMR (500 MHz, CDCl_3_) *δ* 11.55 (br, 1H), 8.51 (s, 1H), 7.99–7.84 (m, 2H), 7.30 (d, *J* = 8.0 Hz, 2H), 3.08 (s, 3H), 2.57 (s, 3H), 2.44 (s, 3H); ^13^C NMR (125 MHz, CDCl_3_) *δ* 187.2, 165.9, 163.7, 161.4, 145.4, 130.7, 129.9, 129.5, 36.7, 36.1, 21.9; IR (KBr, cm^−1^): 3443, 1689, 1608, 1549, 1409, 1358, 1220, 1177, 996, 762. HRMS (ESI) *m*/*z* [M + H]^+^ calcd for C_14_H_16_N_5_O_2_ 286.1304, found 286.1281.

*N-(4-(dimethylamino)-1,3,5-triazin-2-yl)-2-oxo-2-(m-tolyl)acetamide* (**3c**), White solid; mp 241–242 °C; ^1^H NMR (400 MHz, CDCl_3_) *δ* 11.59 (br, 1H), 8.53 (s, 1H), 7.93–7.73 (m, 2H), 7.45 (d, *J* = 7.6 Hz, 1H), 7.41 (t, *J* = 7.6 Hz, 1H), 3.10 (s, 3H), 2.57 (s, 3H), 2.43 (s, 3H); ^13^C NMR (100 MHz, CDCl_3_) *δ* 187.7, 165.9, 163.6, 161.4, 138.7, 135.1, 133.1, 130.3, 128.7, 127.0, 36.7, 36.0, 21.3; IR (KBr, cm^−1^): 3463, 2888, 1693, 1602, 1551, 1410, 1356, 1210, 1180, 996, 774. HRMS (ESI) *m*/*z* [M + H]^+^ calcd for C_14_H_16_N_5_O_2_ 286.1304, found 286.1312.

*N-(4-(dimethylamino)-1,3,5-triazin-2-yl)-2-oxo-2-(o-tolyl)acetamide (***3d***)*, White solid; mp 236–237 °C; ^1^H NMR (500 MHz, CDCl_3_) *δ* 11.28 (br, 1H), 8.50 (s, 1H), 7.80 (d, *J* = 7.5 Hz, 1H), 7.49–7.43 (m, 1H), 7.34–7.27 (m, 2H), 3.08 (s, 3H), 2.69 (s, 3H), 2.45 (s, 3H); ^13^C NMR (125 MHz, CDCl_3_) *δ* 189.2, 165.9, 163.7, 161.4, 141.5, 133.3, 133.1, 132.3, 131.2, 126.0, 36.6, 35.6, 21.7; IR (KBr, cm^−1^): 3486, 1688, 1604, 1548, 1 412, 1343, 729, 600. HRMS (ESI) *m*/*z* [M + H]^+^ calcd for C_14_H_16_N_5_O_2_ 286.1304, found 286.1318

*N-(4-(dimethylamino)-1,3,5-triazin-2-yl)-2-(4-methoxyphenyl)-2-oxoacetamide* (**3e**), White solid; mp 208–209 °C; ^1^H NMR (500 MHz, CDCl_3_) *δ* 8.50 (s, 1H), 8.08–7.95 (m, 2H), 6.97 (d, *J* = 9.0 Hz, 2H), 3.89 (s, 3H), 3.10 (s, 3H), 2.65 (s, 3H); ^13^C NMR (125 MHz, CDCl_3_) *δ* 186.3, 166.0, 164.5, 163.8, 161.5, 132.4, 126.1, 114.1, 55.6, 36.6, 36.1; IR (KBr, cm^−1^): 2848, 1684, 1610, 1551, 1415, 1359, 1265, 1170, 996, 777. HRMS (ESI) *m*/*z* [M + H]^+^ calcd for C_14_H_16_N_5_O_3_ 302.1253, found 302.1259

*N-(4-(dimethylamino)-1,3,5-triazin-2-yl)-2-(3-methoxyphenyl)-2-oxoacetamide* (**3f**), White solid; mp 191–192 °C; ^1^H NMR (400 MHz, CDCl_3_) *δ* 11.44 (br, 1H), 8.51 (s, 1H), 7.66–7.54 (m, 2H), 7.42 (t, *J* = 7.9 Hz, 1H), 7.19 (dd, *J* = 7.9, 2.1 Hz, 1H), 3.11 (s, 3H), 2.60 (s, 3H); ^13^C NMR (100 MHz, CDCl_3_) *δ* 187.1, 165.9, 163.7, 161.4, 159.9, 134.4, 129.9, 122.9, 121.0, 113.3, 55.6, 36.7, 36.0; IR (KBr, cm^−1^): 3461, 2843, 1698, 1675, 1603, 1549, 1426, 1345, 1261, 1000, 810, 763. HRMS (ESI) *m*/*z* [M + H]^+^ calcd for C_14_H_16_N_5_O_3_ 302.1253, found 302.1232.

*N-(4-(dimethylamino)-1,3,5-triazin-2-yl)-2-(4-fluorophenyl)-2-oxoacetamide* (**3g**), White solid; mp 214–215 °C; ^1^H NMR (400 MHz, CDCl_3_) *δ* 8.52 (s, H), 8.16–8.00 (m, 2H), 7.28–6.94 (m, 2H), 3.12 (s, 3H), 2.58 (s, 3H); ^13^C NMR (125 MHz, CDCl_3_) *δ* 184.6, 166.4 (d, *J* = 257.7 Hz), 166.0, 163.8, 161.4, 132.7 (d, *J* = 15.1 Hz), 129.6 (d, *J* = 2.8 Hz), 116.2 (d, *J* = 22.2 Hz), 36.7, 36.1; IR (KBr, cm^−1^): 3850, 1558, 1539, 1127, 1109, 1076, 1062, 1029, 477, 438. HRMS (ESI) *m*/*z* [M + H]^+^ calcd for C_13_H_13_FN_5_O_2_ 290.1053, found 290.1070

*2-(4-chlorophenyl)-N-(4-(dimethylamino)-1,3,5-triazin-2-yl)-2-oxoacetamide* (**3h**), White solid; mp 233–234 °C; ^1^H NMR (400 MHz, CDCl_3_) *δ* 11.36 (br, 1H), 8.50 (s, 1H), 8.10–7.89 (m, 2H), 7.51 (d, *J* = 8.7 Hz, 2H), 3.13 (s, 3H), 2.61 (s, 3H); ^13^C NMR (100 MHz, DMSO-*d*_6_) *δ* 186.6, 169.6, 166.7, 163.6, 162.3, 139.6, 132.1, 131.4, 129.8, 36.6, 35.7. IR (KBr, cm^−1^): 3484, 1693, 1614, 1552, 1410, 1359, 1213, 1175, 996, 769. HRMS (ESI) *m*/*z* [M + H]^+^ calcd for C_13_H_13_ClN_5_O_2_ 306.0758, found 306.0752

*2-(4-bromophenyl)-N-(4-(dimethylamino)-1,3,5-triazin-2-yl)-2-oxoacetamide* (**3i**), White solid; mp 221–222 °C; ^1^H NMR (400 MHz, CDCl_3_) *δ* 11.48 (br, 1H), 8.51 (s, 1H), 8.00–7.80 (m, 2H), 7.68 (d, *J* = 8.5 Hz, 2H), 3.13 (s, 3H), 2.60 (s, 3H); ^13^C NMR (100 MHz, CDCl_3_) *δ* 186.2, 166.0, 163.7, 161.4, 132.2, 131.9, 131.4, 129.7, 36.7, 36.1. IR (KBr, cm^−1^): 3480, 1680, 1607, 1553, 1409, 1354, 1245, 1155, 993, 810. HRMS (ESI) *m*/*z* [M + H]^+^ calcd for C_13_H_13_BrN_5_O_2_ 350.0253, found 350.0259.

*2-(3-bromophenyl)-N-(4-(dimethylamino)-1,3,5-triazin-2-yl)-2-oxoacetamide* (**3j**), White solid; mp 176–177 °C; ^1^H NMR (400 MHz, CDCl_3_) *δ* 8.52 (s, 1H), 8.22–8.11 (m, 1H), 8.03–7.88 (m, 1H), 7.81–7.72 (m, 1H), 7.42 (t, *J* = 8.0 Hz, 1H), 3.13 (s, 3H), 2.60 (s, 3H); ^13^C NMR (100 MHz, CDCl_3_) *δ* 185.8, 165.8, 163.6, 161.3, 137.1, 134.9, 132.5, 130.4, 128.4, 123.0, 36.8, 36.1; IR (KBr, cm^−1^): 3446, 1683, 1609, 1548, 1407, 1356, 1200, 1179, 997, 782. HRMS (ESI) *m*/*z* [M + H]^+^ calcd for C_13_H_13_BrN_5_O_2_ 350.0253, found 350.0250.

*N-(4-(dimethylamino)-1,3,5-triazin-2-yl)-2-oxo-2-(thiophen-2-yl)acetamide* (**3k**), Yellow solid; mp 193–194 °C; ^1^H NMR (400 MHz, CDCl_3_) *δ* 9.61 (br, 1H), 8.54 (s, 1H), 8.41 (d, *J* = 4.2 Hz, 1H), 7.89 (d, *J* = 4.4 Hz, 1H), 7.24 (dd, *J* = 4.4, 4.2 Hz, 1H), 3.22 (s, 3H), 3.17 (s, 3H); ^13^C NMR (100 MHz, CDCl_3_) *δ* 177.4, 166.6, 164.5, 161.8, 139.1, 138.9, 138.6, 128.6, 36.5, 36.3; IR (KBr, cm^−1^): 3461, 2358, 1645, 1614, 1547, 1408, 1360, 1238, 1155, 1054, 996, 722. HRMS (ESI) *m*/*z* [M + H]^+^ calcd for C_11_H_12_N_5_O_2_S 278.0712, found 278.0708.

*N-(4-(diethylamino)-1,3,5-triazin-2-yl)-2-oxo-2-phenylacetamide* (**3l**), Pale yellow solid; mp 165–166 °C; ^1^H NMR (400 MHz, CDCl_3_) *δ* 8.50 (s, 1H), 8.10–7.98 (m, 2H), 7.68–7.61 (m, 1H), 7.54 (t, *J* = 7.4 Hz, 2H), 3.55–3.47 (m, 2H), 3.08–2.88 (m, 2H), 1.11 (t, *J* = 6.8 Hz, 3H), 0.75–0.54 (m, 3H); ^13^C NMR (100 MHz, CDCl_3_) *δ* 187.3, 166.0, 162.7, 161.5, 134.3, 133.0, 130.0, 128.8, 41.9, 41.4, 12.7, 12.1; IR (KBr, cm^−1^): 3457, 1682, 1607, 1544, 1424, 1353, 1213, 1167, 994, 726. HRMS (ESI) *m*/*z* [M + H]^+^ calcd for C_15_H_18_N_5_O_2_ 300.1460, found 300.1465

*2-oxo-2-phenyl-N-(4-(piperidin-1-yl)-1,3,5-triazin-2-yl)acetamide* (**3m**), White solid; mp 222–223 °C; ^1^H NMR (500 MHz, CDCl_3_) *δ* 11.40 (br, 1H), 8.37 (s, 1H), 7.97–7.90 (m, 2H), 7.68–7.61 (m, 1H), 7.53 (t, *J* = 7.4 Hz, 2H), 3.77–3.65 (m, 2H), 3.64–3.53 (m, 2H), 3.33–3.19 (m, 2H), 3.11–2.96 (m, 2H), 2.85–2.72 (m, 2H); ^13^C NMR (125 MHz, CDCl_3_) *δ* 187.4, 166.5, 162.8, 162.1, 134.2, 132.9, 129.4, 128.8, 66.2, 65.9, 43.9, 43.1, 39.9; IR (KBr, cm^−1^): 3466, 1681, 1601, 1548, 1422, 1351, 1218, 1110, 992, 728. HRMS (ESI) *m*/*z* [M + H]^+^ calcd for C_16_H_18_N_5_O_2_ 312.1460, found 312.1434.

*N-(4-morpholino-1,3,5-triazin-2-yl)-2-oxo-2-phenylacetamide* (**3n**), White solid; mp 213–214 °C; ^1^H NMR (500 MHz, CDCl_3_) *δ* 11.46 (br, 1H), 8.51 (s, 1H), 8.08–7.94 (m, 2H), 7.68–7.58 (m, 1H), 7.53 (t, *J* = 7.8 Hz, 2H), 3.82–3.71 (m, 2H), 3.67–3.56 (m, 2H), 3.38–3.32 (m, 2H), 3.15–2.95 (m, 2H); ^13^C NMR (125 MHz, CDCl_3_) *δ* 187.3, 166.4, 163.0, 161.8, 134.5, 132.9, 129.8, 128.9, 66.4, 66.1, 44.0, 43.4; IR (KBr, cm^−1^): 3448, 2860, 1686, 1611, 1543, 1407, 1357, 1201, 1179, 997, 751. HRMS (ESI) *m*/*z* [M + H]^+^ calcd for C_15_H_16_N_5_O_3_ 314.1253, found 314.1263.

*2-oxo-2-phenyl-N-(4-(pyrrolidin-1-yl)-1,3,5-triazin-2-yl)acetamide (***3o***)*, White solid; mp 232–233 °C; ^1^H NMR (500 MHz, CDCl_3_) *δ* 11.70 (br, 1H), 8.51 (s, 1H), 8.07–7.94 (m, 2H), 7.66–7.59 (m, 1H), 7.51 (t, *J* = 7.7 Hz, 2H), 3.53–3.42 (m, 2H), 2.99–2.55 (m, 2H), 1.90–1.78 (m, 2H), 1.78–1.66 (m, 2H); ^13^C NMR (125 MHz, CDCl_3_) *δ* 187.5, 165.8, 161.2, 134.1, 133.2, 129.8, 128.7, 46.6, 45.8, 25.0, 24.7; IR (KBr, cm^−1^): 3466, 1694, 1603, 1539, 1417, 1351, 1327, 1208, 997, 725. HRMS (ESI) *m*/*z* [M + H]^+^ calcd for C_15_H_16_N_5_O_2_ 298.1304, found 298.1293.

*2-oxo-2-phenyl-N-(4-(phenylamino)-1,3,5-triazin-2-yl)acetamide* (**3p**), Yellow solid; mp 209–210 °C; ^1^H NMR (400 MHz, DMSO-*d*_6_) *δ* 11.84 (br, 1H), 10.29 (s, 1H), 8.29 (s, 1H), 7.90–7.75 (m, 2H), 7.70–7.43 (m, 6H), 7.33–7.20 (m, 1H), 7.11–7.04 (m, 1H); ^13^C NMR (100 MHz, DMSO-*d*_6_) *δ* 188.0, 166.6, 163.8, 162.8, 138.5, 137.6, 134.6, 133.4, 129.5, 129.0, 126.1, 121.0; IR (KBr, cm^−1^): 3423, 1681, 1587, 1538, 1453, 1417, 1352, 1215, 1177, 1002, 813, 725. HRMS (ESI) *m*/*z* [M + H]^+^ calcd for C_17_H_14_N_5_O_2_ 320.1147, found 320.1128.

### 3.3. General Procedure for the Synthesis of N-([1,3,5]Triazine-2-yl) Amides 4

A mixture of 1,3,5-triazine (0.5 mmol), ketone (1.1 mmol), CuCl_2_ (0.20 mmol), and I_2_ (0.75 mmol) was added in 1,2-dichlorobenzene (2 mL) and bis(2-methoxy ethyl)ether (1 mL). The resulting mixture was then sealed and stirred at 140 °C under O_2_. After completion of the reaction, the reaction mixture was cooled to room temperature, 10% Na_2_S_2_O_3_ was added and the mixture was extracted with ethyl acetate (4 × 20 mL). The organic phase was dried over anhydrous Na_2_SO_4_. The crude residue was obtained after the evaporation of the solvent in vacuum, and the residue was purified using flash chromatography with petroleum ether/ethyl acetate as the eluent to give the pure product.

*N-(4-(dimethylamino)-1,3,5-triazin-2-yl)benzamide* (**4a**), White solid; mp 186–188 °C ([lit]^27^: 187–189 °C); ^1^H NMR (500 MHz, CDCl_3_) δ 8.85 (br, 1H), 8.32 (s, 1H), 7.88 (dt, *J* = 7.5, 1.0 Hz, 2H), 7.57 (tt, *J* = 7.5, 1.0 Hz, 1H), 7.48 (t, *J* = 7.5 Hz, 2H), 3.20 (s, 3H), 3.17 (s, 3H).

*N-(4-(dimethylamino)-1,3,5-triazin-2-yl)-4-methylbenzamide* (**4b**), White solid; mp 186–188 °C ([lit]^27^: 182–184 °C); ^1^H NMR (400 MHz, CDCl_3_) δ 8.59 (br, 1H), 8.38 (s, 1H), 7.79 (d, *J* = 8.4 Hz, 2H), 7.28 (d, *J* = 8.4 Hz, 2H), 3.21 (s, 3H), 3.20 (s, 3H), 2.42 (s, 3H).

*N-(4-(dimethylamino)-1,3,5-triazin-2-yl)-3-methylbenzamide* (**4c**), White solid; mp 119–121 °C; ^1^H NMR (400 MHz, CDCl_3_) *δ* 8.79 (br, 1H), 8.34–8.30 (m, 1H), 7.72–7.62 (m, 2H), 7.39–7.34 (m, 2H), 3.19 (s, 6H), 2.42 (s, 3H); ^13^C NMR (100 MHz, CDCl_3_) *δ* 166.5, 165.9, 164.8, 162.8, 138.8, 134.4, 133.4, 128.7, 128.4, 124.7, 36.5, 36.3, 21.4; IR (KBr, cm^−1^): 3177, 2933, 1712, 1616, 1543, 1402, 1276, 1217, 1182, 1032, 813, 753. HRMS (ESI) *m*/*z* [M + H]^+^ calcd for C_13_H_16_N_5_O 258.1355, found 258.1356.

*N-(4-(dimethylamino)-1,3,5-triazin-2-yl)-3-methoxybenzamide* (**4d**), White solid; mp 116–119 °C; ^1^H NMR (400 MHz, CDCl_3_) *δ* 8.74 (br, 1H), 8.35 (s, 1H), 7.45–7.41 (m, 2H), 7.38 (t, *J* = 7.9 Hz, 1H), 7.12–7.09 (m, 1H), 3.86 (s, 3H), 3.20 (s, 3H), 3.19 (s, 3H); ^13^C NMR (100 MHz, CDCl_3_) *δ* 166.6, 165.4, 164.8, 162.8, 160.1, 135.8, 129.9, 119.5, 118.9, 113.0, 55.6, 36.5, 36.4; IR (KBr, cm^−1^): 3174, 2945, 1711, 1615, 1539, 1402, 1275, 1181, 1114, 983, 813, 755. HRMS (ESI) *m*/*z* [M + H]^+^ calcd for C_13_H_16_N_5_O_2_ 274.1304, found 274.1293.

*N-(4-(dimethylamino)-1,3,5-triazin-2-yl)-4-fluorobenzamide* (**4e**), White solid; mp 175–177 °C ([lit]^27^: 173–178 °C); ^1^H NMR (400 MHz, CDCl_3_) *δ* 8.80 (br, 1H), 8.35 (s, 1H), 7.96–7.87 (m, 2H), 7.21–7.12 (m, 2H), 3.20 (s, 3H), 3.17 (s, 3H).

*N-(4-(dimethylamino)-1,3,5-triazin-2-yl)-2-fluorobenzamide* (**4f**), White solid; mp 178–180 °C; ^1^H NMR (400 MHz, CDCl_3_) *δ* 9.01 (br, 1H), 8.45 (s, 1H), 8.10–8.05 (m, 1H), 7.57–7.51 (m, 1H), 7.33–7.27 (m, 1H), 7.20–7.15 (m, 1H), 3.21 (s, 3H), 3.14 (s, 3H); ^13^C NMR (100 MHz, CDCl_3_) *δ* 166.6, 164.8, 162.6, 161.9, 160.5 (d, *J* = 247.3 Hz), 134.2 (d, *J* = 9.2 Hz), 132.2 (d, *J* = 2.1 Hz), 125.3 (d, *J* = 3.3 Hz), 121.9 (d, *J* = 12.0 Hz), 116.3 (d, *J* = 24.1 Hz), 36.5, 36.3; IR (KBr, cm^−1^): 3100, 2930, 1683, 1604, 1551, 1493, 1413, 1341, 1219, 1136, 992, 751, 625. HRMS (ESI) *m*/*z* [M + H]^+^ calcd for C_12_H_13_FN_5_O 262.1104, found 262.1068.

*4-chloro-N-(4-(dimethylamino)-1,3,5-triazin-2-yl)benzamide* (**4g**), White solid; mp 183–185 °C ([lit]^27^: 176–177 °C); ^1^H NMR (400 MHz, CDCl_3_) *δ* 8.78 (br, 1H), 8.37 (s, 1H), 7.83 (d, *J* = 8.6 Hz, 2H), 7.46 (d, *J* = 8.6 Hz, 2H), 3.21 (s, 3H), 3.16 (s, 3H).

*3-chloro-N-(4-(dimethylamino)-1,3,5-triazin-2-yl)benzamide* (**4h**), White solid; mp 149–151 °C ([lit]^27^: 163–165 °C); ^1^H NMR (400 MHz, CDCl_3_) *δ* 9.13 (br, 1H), 8.40 (s, 1H), 7.95–7.90 (m, 1H), 7.80 (d, *J* = 7.8 Hz, 1H), 7.58–7.52 (m, 1H), 7.47–7.40 (m, 1H), 3.22 (s, 3H), 3.19 (s, 3H).

*4-bromo-N-(4-(dimethylamino)-1,3,5-triazin-2-yl)benzamide* (**4i**), White solid; mp 191–192 °C; ^1^H NMR (400 MHz, CDCl_3_) *δ* 9.04 (br, 1H), 8.34 (s, 1H), 7.75 (d, *J* = 8.6 Hz, 2H), 7.61 (d, *J* = 8.6 Hz, 2H), 3.20 (s, 3H), 3.13 (s, 3H); ^13^C NMR (100 MHz, CDCl_3_) *δ* 166.4, 165.3, 164.7, 162.7, 133.4, 132.1, 129.4, 127.4, 36.5, 36.3; IR (KBr, cm^−1^): 3101, 2874, 1668, 1611, 1542,1427, 1339, 1124, 994, 758, 707, 647. HRMS (ESI) *m*/*z* [M + H]^+^ calcd for C_12_H_13_BrN_5_O 322.0303, found 322.0275.

*3-bromo-N-(4-(dimethylamino)-1,3,5-triazin-2-yl)benzamide* (**4j**), White solid; mp 152–153 °C; ^1^H NMR (400 MHz, CDCl_3_) *δ* 8.82 (br, 1H), 8.39 (s, 1H), 8.08–8.01 (m, 1H), 7.82–7.80 (m, 1H), 7.71–7.68 (m, 1H), 7.37 (t, *J* = 7.9 Hz, 1H), 3.21 (s, 3H), 3.16 (s, 3H); ^13^C NMR (100 MHz, CDCl_3_) *δ* 166.3, 165.0, 164.6, 162.6, 136.5, 135.4, 131.0, 130.3, 126.4, 122.9, 36.5, 36.3; IR (KBr, cm^−1^): 3211, 3060, 2923, 1731, 1636, 1543, 1411, 1310, 1256, 1183, 993, 807, 747. HRMS (ESI) *m*/*z* [M + H]^+^ calcd for C_12_H_13_BrN_5_O 322.0303, found 322.0274.

*N-(4-(dimethylamino)-1,3,5-triazin-2-yl)-4-nitrobenzamide* (**4k**), Light yellow solid (87.0 mg, 61%), mp 237–240 °C. ^1^H NMR (400 MHz, DMSO-*d_6_*) *δ* 11.12 (br, 1H), 8.43 (s, 1H), 8.31 (d, *J* = 8.5 Hz, 2H), 8.05 (d, *J* = 8.5 Hz, 2H), 3.11 (s, 3H), 2.98 (s, 3H); ^13^C NMR (100 MHz, DMSO-*d_6_*) *δ* 166.4, 165.5, 164.2, 163.2, 149.2, 140.5, 129.6, 123.4, 35.8, 35.4; IR (KBr, cm^−1^): 3106, 2881, 1676, 1611, 1540,1521, 1417, 1328, 1314, 1124, 993, 863, 751, 645. HRMS (ESI) *m*/*z* [M + H]^+^ calcd for C_12_H_13_N_6_O_3_ 289.1049, found 289.1081

*N-(4-(piperidin-1-yl)-1,3,5-triazin-2-yl)benzamide* (**4l**), White solid; mp 166–168 °C ([lit]^27^: 164–166 °C); ^1^H NMR (400 MHz, CDCl_3_) *δ* 8.85 (br, 1H), 8.49–8.18 (m, 1H), 7.88 (d, *J* = 7.8 Hz, 2H), 7.57 (t, *J* = 7.8 Hz, 1H), 7.48 (t, *J* = 7.8 Hz, 2H), 3.93–3.63 (m, 4H), 1.70–1.60 (m, 6H).

*N-(4-morpholino-1,3,5-triazin-2-yl)benzamide* (**4m**), White solid; mp 178–179 °C; ^1^H NMR (400 MHz, CDCl_3_) *δ* 8.76 (br, 1H), 8.36–8.34 (m, 1H), 7.90–7.87 (m, 2H), 7.60–7.55 (m, 1H), 7.53–7.45 (m, 2H), 3.93–3.80 (m, 4H), 3.78–3.70 (m, 4H); ^13^C NMR (100 MHz, CDCl_3_) *δ* 166.8, 165.7, 164.1, 163.0, 134.3, 132.6, 128.8, 127.7, 66.6, 43.8, 43.6; IR (KBr, cm^−1^): 3172, 2910, 2853, 1717, 1610, 1534, 1424, 1268, 1209, 1114, 980, 810, 697. HRMS (ESI) *m*/*z* [M + H]^+^ calcd for C_14_H_16_N_5_O_2_ 286.1304, found 286.1304.

*4-fluoro-N-(4-(pyrrolidin-1-yl)-1,3,5-triazin-2-yl)benzamide* (**4n**), White solid; mp 197–199 °C; ^1^H NMR (400 MHz, CDCl_3_) *δ* 8.85 (br, 1H), 8.34 (s, 1H), 7.93–7.88 (m, 2H), 7.26–7.11 (m, 2H), 3.60–3.52 (m, 4H), 2.00–1.94 (m, 4H); ^13^C NMR (100 MHz, CDCl_3_) *δ* 166.3, 165.4 (d, *J* = 254.8 Hz), 164.9, 162.7, 162.5, 130.6 (d, *J* = 3.2 Hz), 130.4 (d, *J* = 9.1 Hz), 116.0 (d, *J* = 21.9 Hz), 46.6, 46.5, 25.3, 25.2; IR (KBr, cm^−1^): 3109, 2974, 2882, 1670, 1603, 1537, 1422, 1326, 1230, 1157, 994, 846, 760. HRMS (ESI) *m*/*z* [M + H]^+^ calcd for C_14_H_15_FN_5_O 288.1261, found 288.1248.

### 3.4. Synthesis of 2-oxo-2-phenylacetaldehyde *(**5**)*

A mixture of acetophenone (1.0 mmol, 121.3 mg), CuCl (0.2 mmol, 20.1 mg) and I_2_ (2.0 mmol, 508.6 mg) was added in DMSO (4 mL). The resulting mixture was then stirred at 120 °C for 1.5 h under N_2_. After completion of the reaction, the reaction mixture was cooled to room temperature, 10% Na_2_S_2_O_3_ was added and the mixture was extracted with ethyl acetate (4 × 20 mL). The organic phase was dried over anhydrous Na_2_SO_4_ and concentrated in vacuo. The residue was purified using flash chromatography with petroleum ether/ethyl acetate as the eluent to give **5** [31] (85.3 mg, 63% yield). ^1^H NMR (400 MHz, CDCl_3_) *δ* 9.67 (s, 1H), 8.21–8.19 (m, 2H), 7.70–7.65 (m, 3H).

## 4. Conclusions

In summary, we found that oxidative α-ketoamidation and oxidative C−C bond cleavage amidation of ketones with 2-amino[1,3,5]triazines to form *N*-([1,3,5]triazine-2-yl) α-ketoamide and *N*-([1,3,5]triazine-2-yl) amide derivatives are available by using a suitable reaction condition. The new method tolerates a variety of functional groups of the substrate and furnished moderate to good yields of the corresponding products under mild conditions. Work is currently ongoing to investigate the use of afforded products in the fields of medicinal chemistry.

## Figures and Tables

**Table 1:** Optimization of the reaction conditions for the formation of **3a** ^a^.

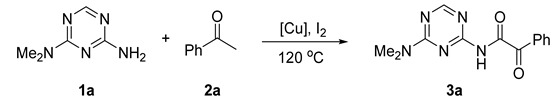					
Entry	[Cu] (mol%)	I_2_ (Eq.)	Solvent	Time (h)	Yield of 3a (%)
1	CuCl (20)	2.0	DMSO	1.5	90
2	CuCl (20)	2.0	PhCl	1.5	trace
3	CuCl (20)	2.0	1,2-DCB	1.5	trace
4	CuCl (20)	2.0	Toluene	1.5	trace
5	CuCl (20)	2.0	DMF	1.5	trace
6	CuCl (20)	2.0	Dioxane	1.5	trace
7	CuCl (20)	2.0	DMSO	3	63
8	CuCl (20)	2.0	DMSO	1.0	88
9	CuCl (20)	2.0	DMSO	1.0	79 ^b^
10	CuCl (20)	2.0	DMSO	1.5	69 ^c^
11	CuBr (20)	2.0	DMSO	1.5	82
12	CuI (20)	2.0	DMSO	1.5	77
13	Cu(OAc)_2_ (20)	2.0	DMSO	1.5	69
14	CuCl_2_ (20)	2.0	DMSO	1.5	80
15	CuCl (10)	2.0	DMSO	1.5	64
16	CuCl (20)	2.0	DMSO	1.5	69 ^d^
17	CuCl (20)	1.0	DMSO	1.5	53
18	CuCl (20)	2.0	DMSO	1.5	75 ^e^
19	CuCl (20)	2.0	DMSO	1.5	48 ^f^

^a^ Reaction Conditions: **1a** (0.5 mmol), **2a** (1.1 mmol), [Cu] (10–20 mol%), I_2_ (1.0–2.0 eq.), solvent (4 mL) under N_2_; isolated yields. ^b^ 130 °C. ^c^ 100 °C. ^d^ **2a** (0.7 mmol). ^e^ Air. ^f^ O_2_.

**Table 2:** Optimization of the reaction conditions for the formation of **4a** ^a^.

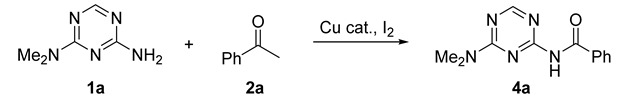						
Entry	Cat. (Mol%)	I_2_ (Eq.)	Solvent	Temp. (°C)	Time (h)	Yield (%)
1	CuCl (20)	1	DMSO	120	13	trace
2	CuCl (20)	1	1,2-DCB	120	13	trace
3	CuI (20)	1	1,2-DCB	120	13	trace
4	CuBr (20)	1	1,2-DCB	120	13	trace
5	Cu_2_(OAc)_4_ (20)	1	1,2-DCB	120	13	trace
6	CuCl_2_ (20)	1	1,2-DCB	120	13	27
7	CuCl_2_ (20)	1	Diglyme	120	13	11
8	CuCl_2_ (20)	1	1,2,4-TCB	120	13	trace
9	CuCl_2_ (20)	1	DMF	120	13	trace
10	CuCl_2_ (20)	1	NMP	120	13	trace
11	CuCl_2_ (20)	1	Toluene	120	13	trace
12	CuCl_2_ (20)	1	1,2-DCB/diglyme = 2:1	120	13	43
13	CuCl_2_ (40)	1	1,2-DCB/diglyme = 2:1	140	13	63
14	CuCl_2_ (40)	1	1,2-DCB/diglyme = 2:1	150	13	27
15	CuCl_2_ (40)	0.75	1,2-DCB/diglyme = 2:1	140	13	67
16	CuCl_2_ (40)	0.5	1,2-DCB/diglyme = 2:1	140	13	40
17	CuCl_2_ (50)	0.75	1,2-DCB/diglyme = 2:1	140	13	61
18	CuCl_2_ (40)	0.75	1,2-DCB/diglyme = 2:1	140	18	13
19	CuCl_2_ (40)	0.75	1,2-DCB/diglyme = 2:1	140	8	34
20	CuCl_2_ (40)	-	1,2-DCB/diglyme = 2:1	140	13	0
21	-	0.75	1,2-DCB/diglyme = 2:1	140	13	0
22	CuCl_2_ (40)	0.75	1,2-DCB/diglyme = 2:1	140	13	trace ^b^
23	CuCl_2_ (40)	0.75	1,2-DCB/diglyme = 2:1	140	13	trace ^c^

^a^ Reaction Conditions: a mixture of **1a** (0.5 mmol), **2a** (1.1 mmol), I_2_ and Cu salts in solvent (3 mL) was stirred under O_2_ (1 atm); isolated yields are shown. ^b^ N_2_. ^c^ Air.

## Data Availability

The data presented in this study are available in the article and Supplementary Materials.

## Supplementary Materials

The following supporting information can be downloaded at: https://www.mdpi.com/article/10.3390/molecules28114338/s1, crystal data, copies of NMR spectra, IR spectra, and HRMS spectra.

## Sample Availability

Samples of the compounds **3a**–**p** and **4a**–**n** are available from the authors.
