# Supplementary material for: Selective Synthesis of N-[1,3,5]Triazinyl-α-Ketoamides and N-[1,3,5]Triazinyl-Amides from the Reactions of 2-Amine-[1,3,5]Triazines with Ketones
Source: Molecules. 2023 May 25;28(11):4338. doi: 10.3390/molecules28114338 (PMC10254511; doi:10.3390/molecules28114338)

Supporting Information

For

Selective synthesis of *N*-[1,3,5]Triazinyl-α-ketoamides and *N*-[1,3,5]Triazinyl-amides from the reactions of 2-amine-[1,3,5]triazines with ketones

Yue Li, Pengzhen Zhong, Junna Zhao, Zexi Pan, Chen Zhang, and Dongmei Cui

checkCIF/PLATON report

Structure factors have been supplied for datablock(s) mo_210507_pzc_2_0m

THIS REPORT IS FOR GUIDANCE ONLY. IF USED AS PART OF A REVIEW PROCEDURE FOR PUBLICATION, IT SHOULD NOT REPLACE THE EXPERTISE OF AN EXPERIENCED CRYSTALLOGRAPHIC REFEREE.

No syntax errors found. CIF dictionary Interpreting this report

Datablock: mo_210507_pzc_2_0m

```
Bond precision:  C-C = 0.0016 A         Wavelength=0.71073

Cell:            a=7.6548(2)    b=12.1882(3)       c=14.4395(4)
                 alpha=90       beta=103.691(1)    gamma=90
Temperature:     170 K

                Calculated                 Reported
Volume          1308.90(6)                 1308.90(6)
Space group     P 21/c                     P 1 21/c 1
Hall group      -P 2ybc                    -P 2ybc
Moiety formula  C13 H13 N5 O2              C13 H13 N5 O2
Sum formula     C13 H13 N5 O2              C13 H13 N5 O2
Mr              271.28                     271.28
Dx,g cm-3       1.377                      1.377
Z               4                          4
Mu (mm-1)       0.098                      0.098
F000            568.0                      568.0
F000'           568.22
h,k,lmax        9,15,18                    9,15,18
Nref            2895                       2894
Tmin,Tmax       0.957,0.966                0.687,0.746
Tmin'           0.957

Correction method= # Reported T Limits: Tmin=0.687 Tmax=0.746
AbsCorr = MULTI-SCAN

Data completeness= 1.000          Theta(max)= 27.099

R(reflections)= 0.0356( 2569)     wR2(reflections)= 0.0989( 2894)

S = 1.066                  Npar= 183
```

The following ALERTS were generated. Each ALERT has the format
test-name_ALERT_alert-type_alert-level.
Click on the hyperlinks for more details of the test.

Alert level C

```
PLAT369_ALERT_2_C Long   C(sp2)-C(sp2) Bond  C7       - C8       .       1.53 Ang.
```

Alert level G

```
PLAT007_ALERT_5_G Number of Unrefined Donor-H Atoms .............          1 Report
PLAT432_ALERT_2_G Short Inter X...Y Contact  C5       ..C5               3.15 Ang.
                                                1-x,1-y,2-z  =      3_667 Check
PLAT910_ALERT_3_G Missing # of FCF Reflection(s) Below Theta(Min).         1 Note
PLAT933_ALERT_2_G Number of OMIT Records in Embedded .res File ...         1 Note
PLAT978_ALERT_2_G Number C-C Bonds with Positive Residual Density.         6 Info
```

```
   0 ALERT level A = Most likely a serious problem - resolve or explain
   0 ALERT level B = A potentially serious problem, consider carefully
   1 ALERT level C = Check. Ensure it is not caused by an omission or oversight
   5 ALERT level G = General information/check it is not something unexpected

   0 ALERT type 1 CIF construction/syntax error, inconsistent or missing data
   4 ALERT type 2 Indicator that the structure model may be wrong or deficient
   1 ALERT type 3 Indicator that the structure quality may be low
   0 ALERT type 4 Improvement, methodology, query or suggestion
   1 ALERT type 5 Informative message, check
```

It is advisable to attempt to resolve as many as possible of the alerts in all categories. Often the minor alerts point to easily fixed oversights, errors and omissions in your CIF or refinement strategy, so attention to these fine details can be worthwhile. In order to resolve some of the more serious problems it may be necessary to carry out additional measurements or structure refinements. However, the purpose of your study may justify the reported deviations and the more serious of these should normally be commented upon in the discussion or experimental section of a paper or in the "special_details" fields of the CIF. checkCIF was carefully designed to identify outliers and unusual parameters, but every test has its limitations and alerts that are not important in a particular case may appear. Conversely, the absence of alerts does not guarantee there are no aspects of the results needing attention. It is up to the individual to critically assess their own results and, if necessary, seek expert advice.

Publication of your CIF in IUCr journals

A basic structural check has been run on your CIF. These basic checks will be run on all CIFs submitted for publication in IUCr journals (*Acta Crystallographica*, *Journal of Applied Crystallography*, *Journal of Synchrotron Radiation*); however, if you intend to submit to *Acta Crystallographica Section C* or *E* or *IUCrData*, you should make sure that full publication checks are run on the final version of your CIF prior to submission.

Publication of your CIF in other journals

Please refer to the *Notes for Authors* of the relevant journal for any special instructions relating to CIF submission.

PLATON version of 22/03/2021; check.def file version of 19/03/2021

Datablock mo_210507_pzc_2_0m - ellipsoid plot

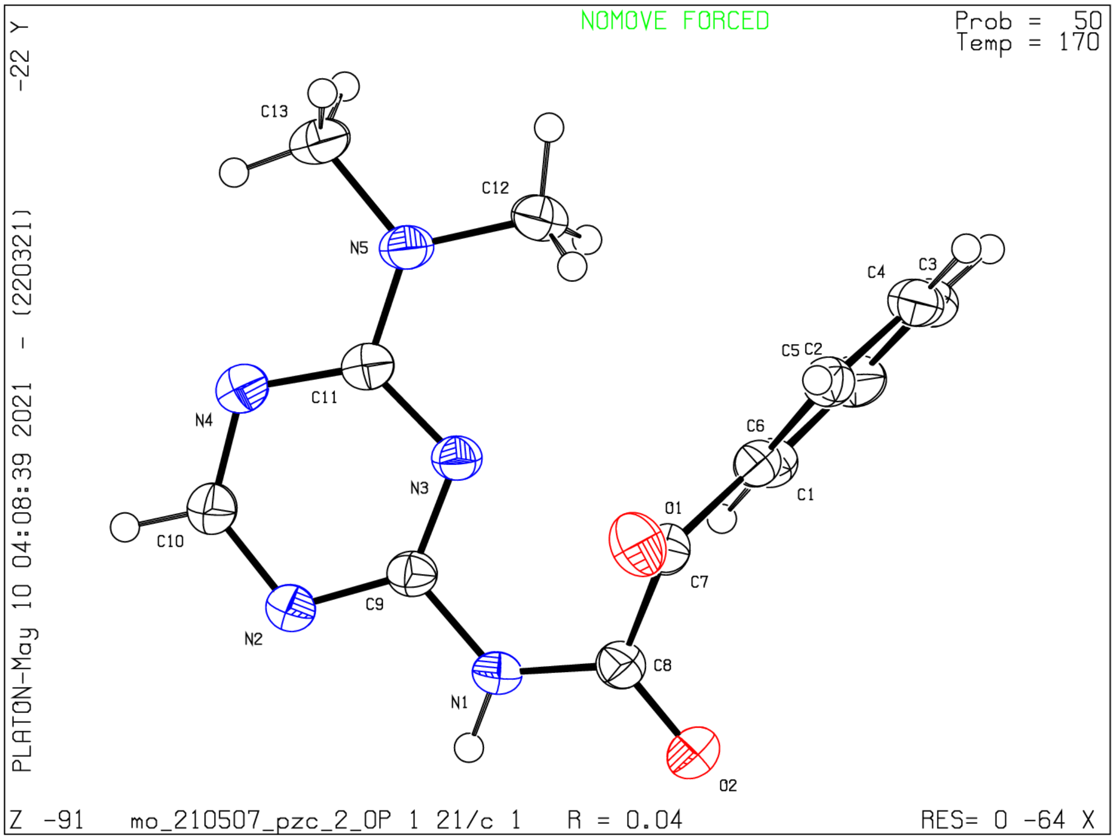

ZWQ200603-b CDCl3

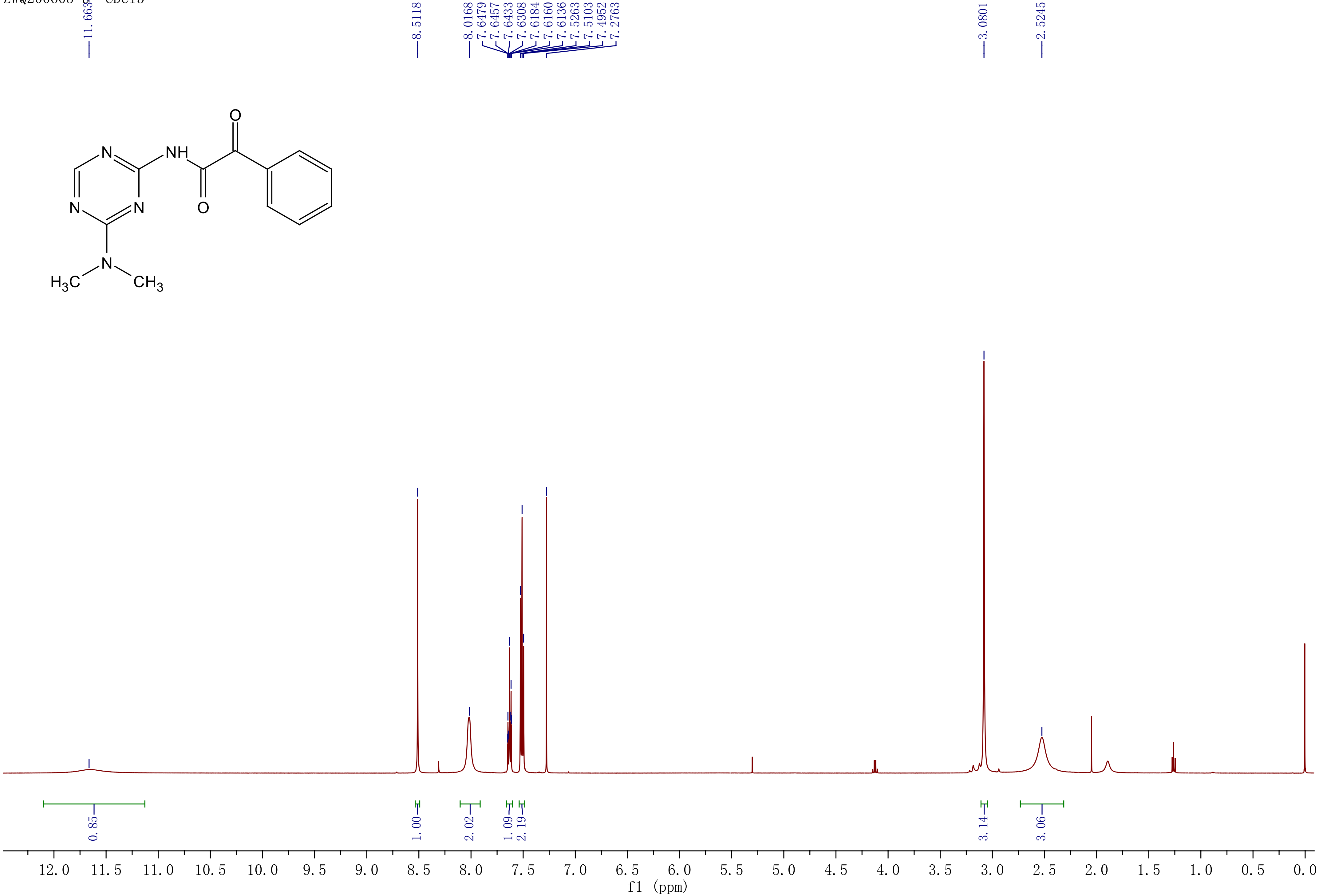

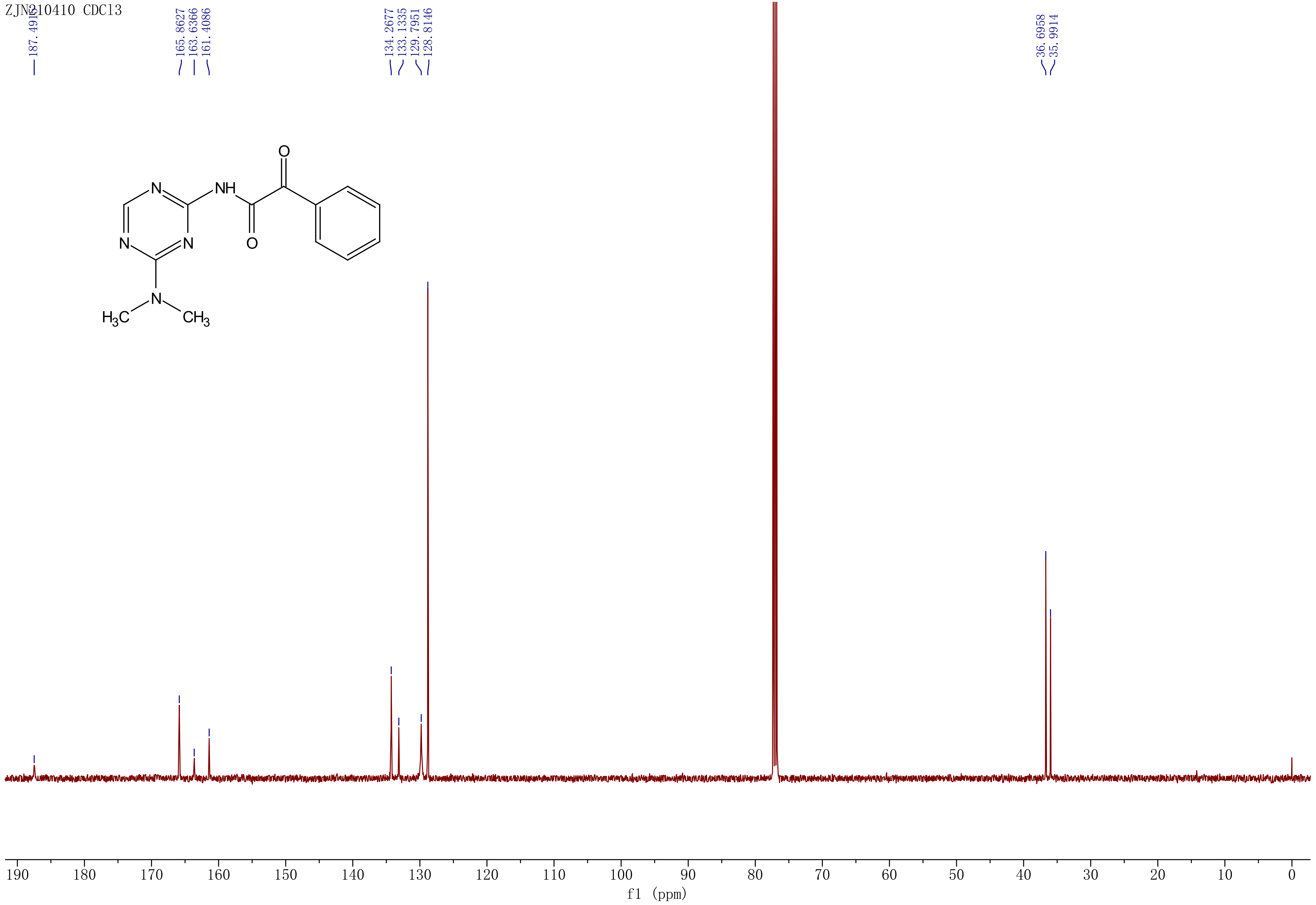
ZJN210410 CDCl3
187.4915
165.8627
163.6366
161.4086
134.2677
133.1335
129.7951
128.8146
36.6958
35.9914
N
NH
O
N
N
O
N
H3C
CH3
190
180
170
160
150
140
130
120
110
100
90
80
70
60
50
40
30
20
10
0
f1 (ppm)

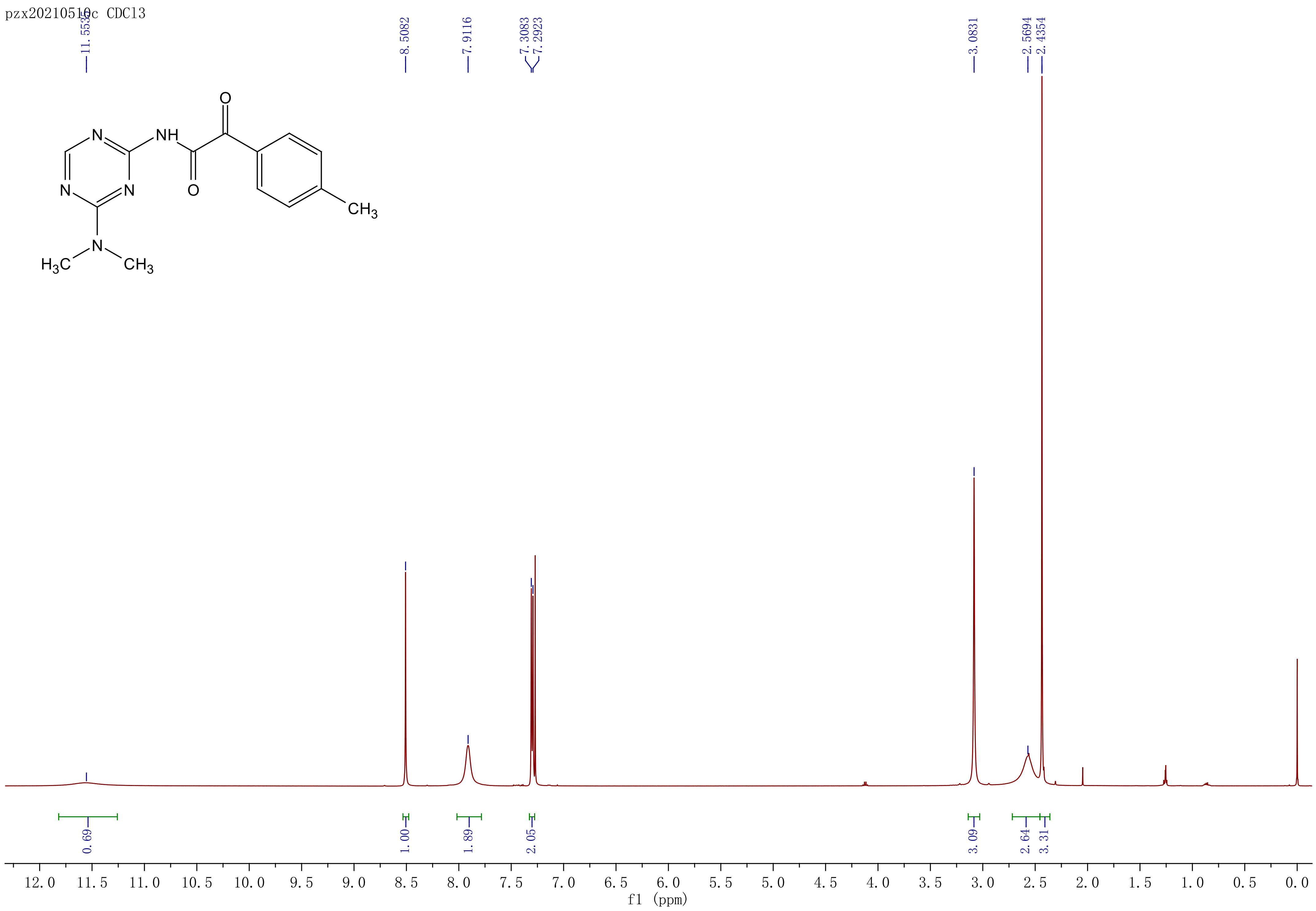

pzx20210510c CDCl3
11.553
8.5082
7.9116
7.3083
7.2923
3.0831
2.5694
2.4354
NH
O
O
N
N
N
N
CH3
H3C
CH3
0.69
1.00
1.89
2.05
3.09
2.64
3.31
12.0
11.5
11.0
10.5
10.0
9.5
9.0
8.5
8.0
7.5
7.0
6.5
6.0
5.5
5.0
4.5
4.0
3.5
3.0
2.5
2.0
1.5
1.0
0.5
0.0
f1 (ppm)

pzx20210510c CDCl3

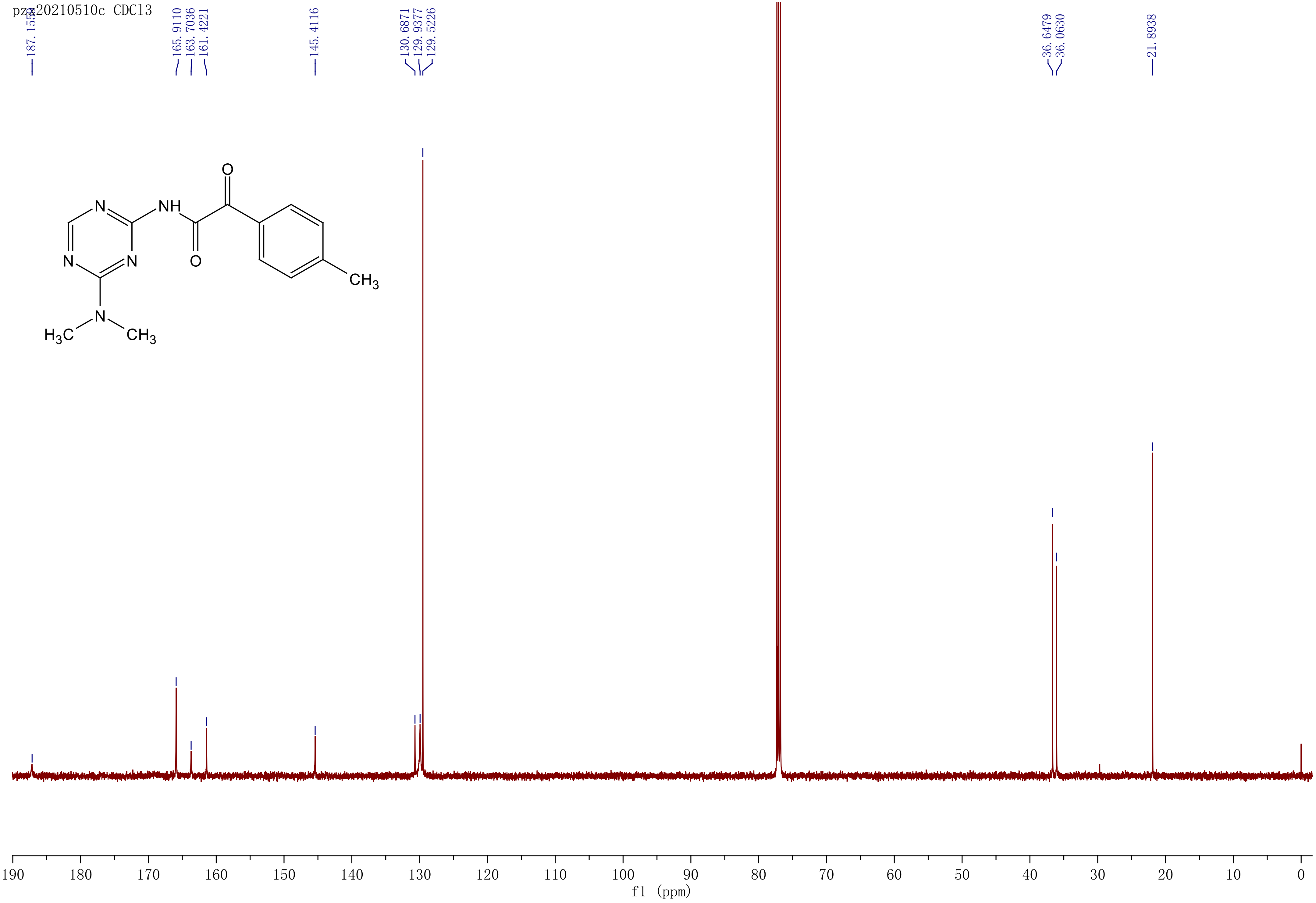

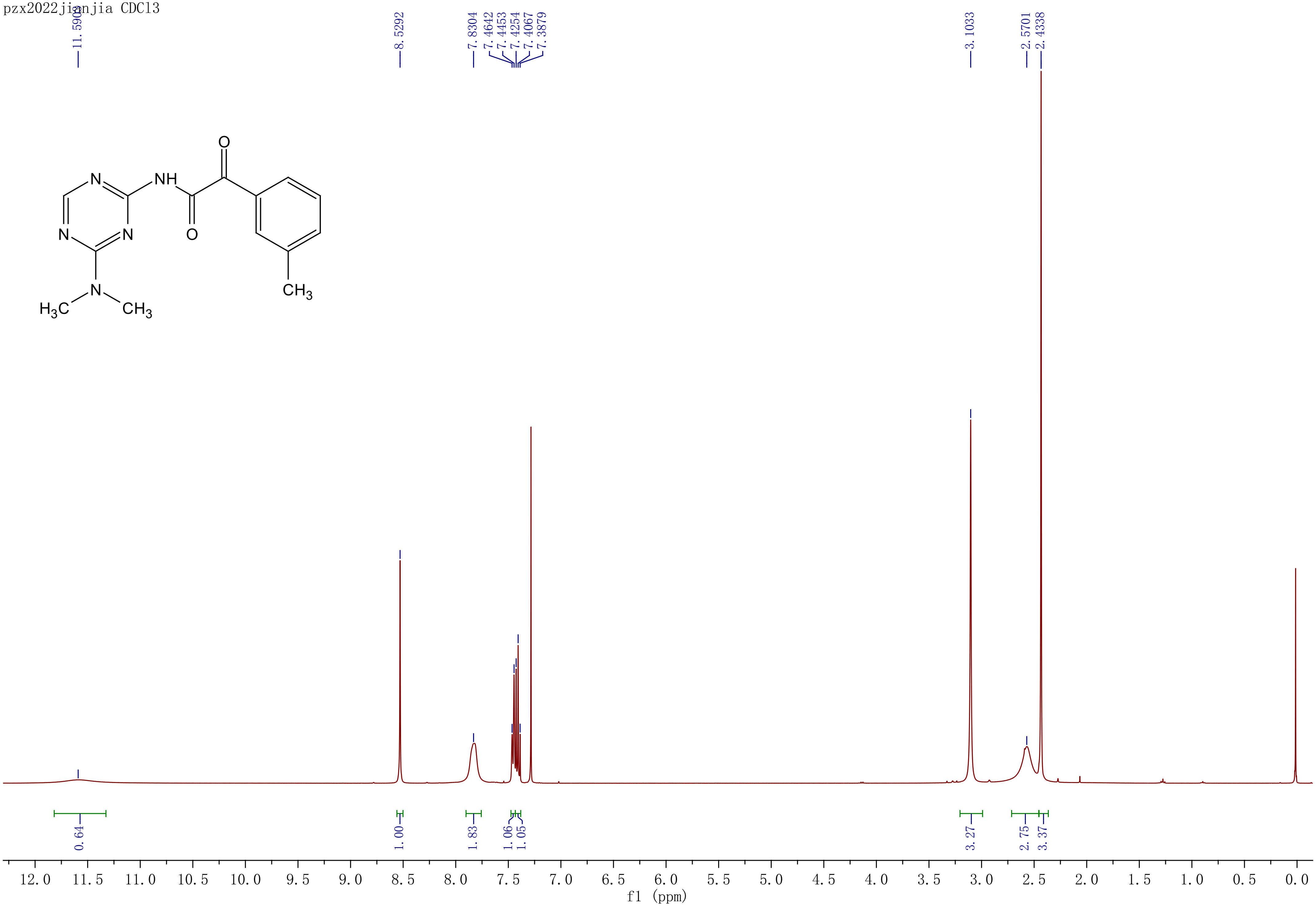
pzx2022jianjia CDCl3
11.5904
8.5292
7.8304
7.4642
7.4453
7.4254
7.4067
7.3879
3.1033
2.5701
2.4338
O
N
NH
N
N
O
N
H3C
CH3
CH3
0.64
1.00
1.83
1.06
1.05
3.27
2.75
3.37
12.0
11.5
11.0
10.5
10.0
9.5
9.0
8.5
8.0
7.5
7.0
6.5
6.0
5.5
5.0
4.5
4.0
3.5
3.0
2.5
2.0
1.5
1.0
0.5
0.0
f1 (ppm)

pzx2021jianjia CDCl3

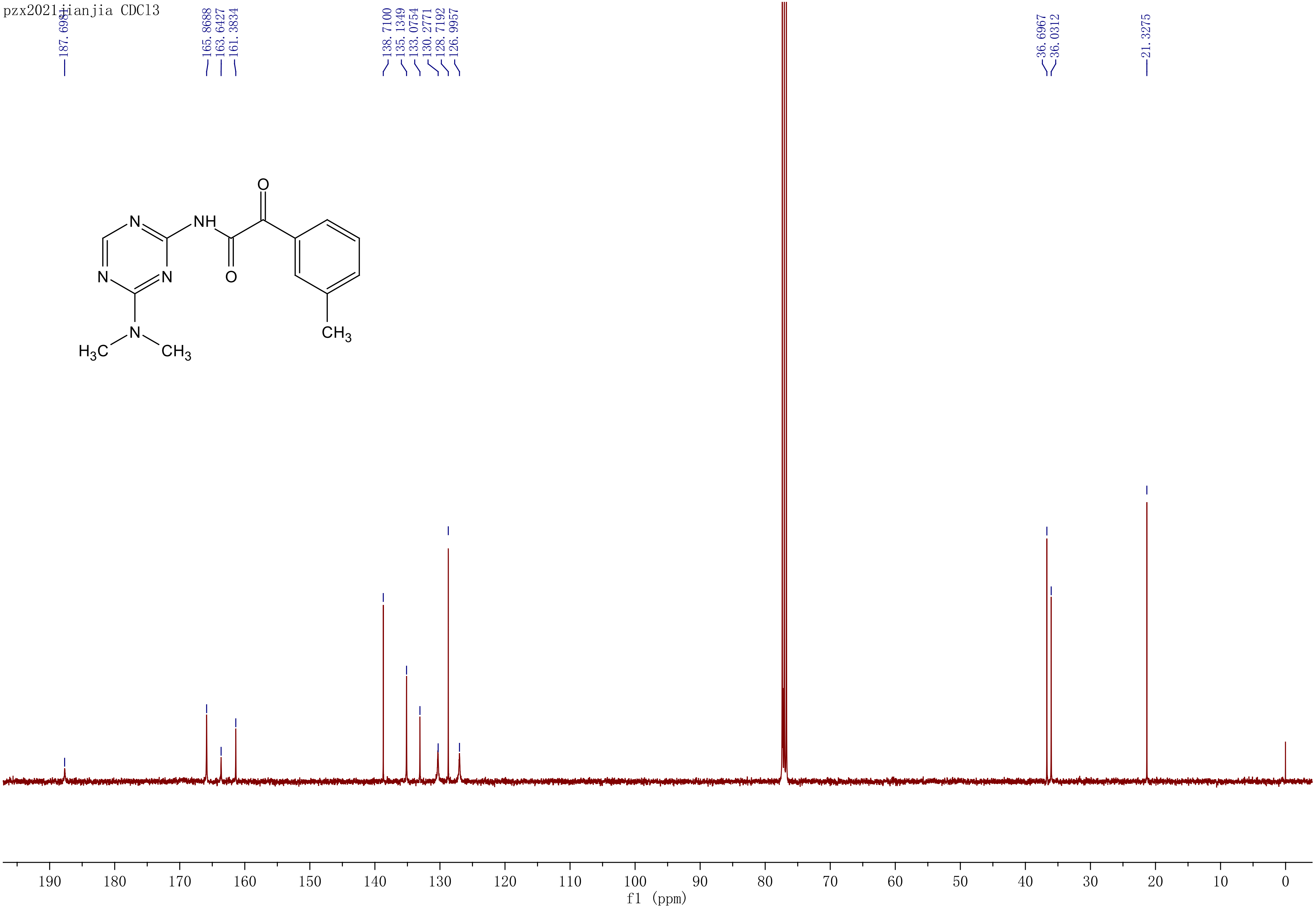

PZX20211229 CDCl3

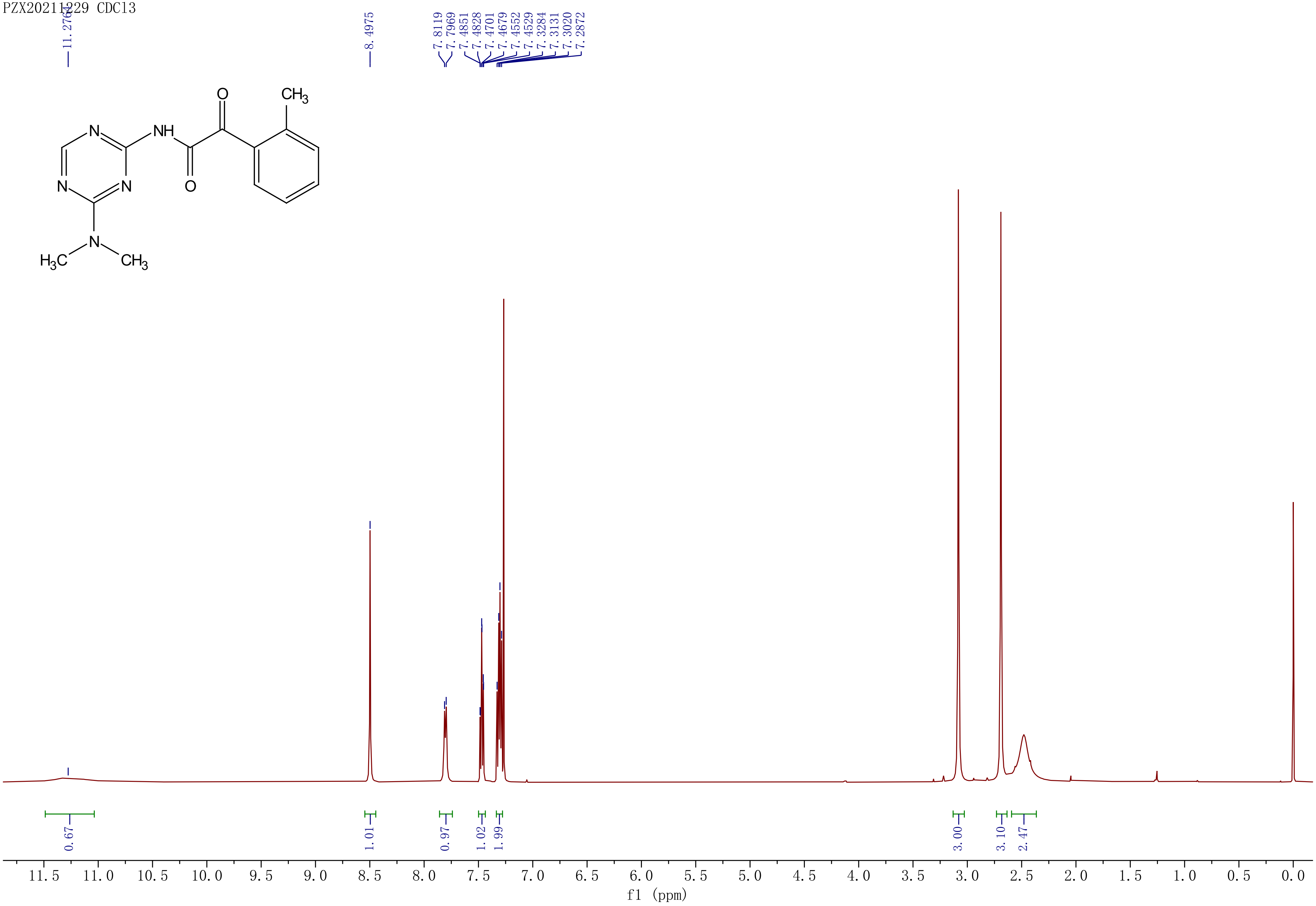

PZX20211229 CDCl3

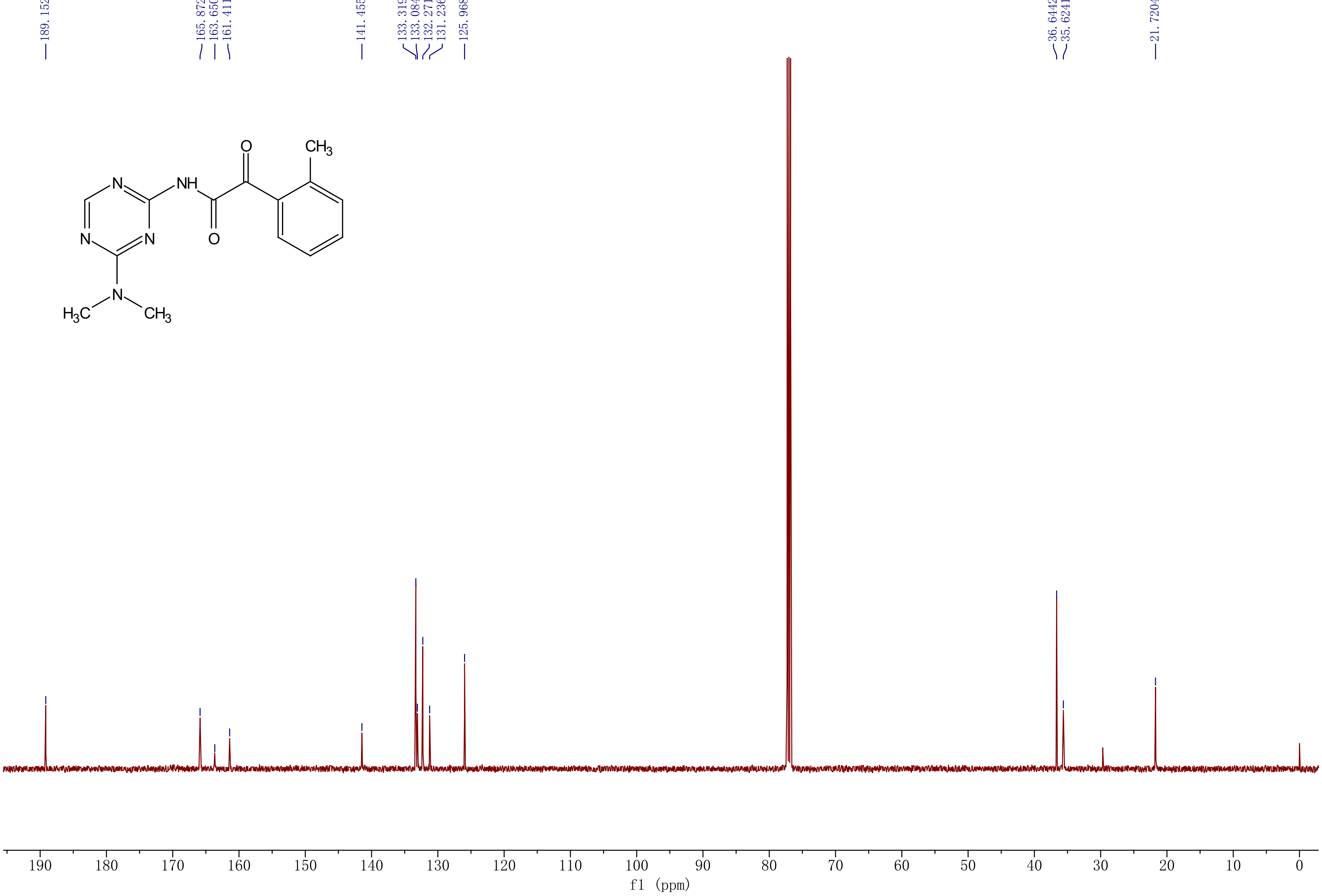

pzx20211019a CDCl3

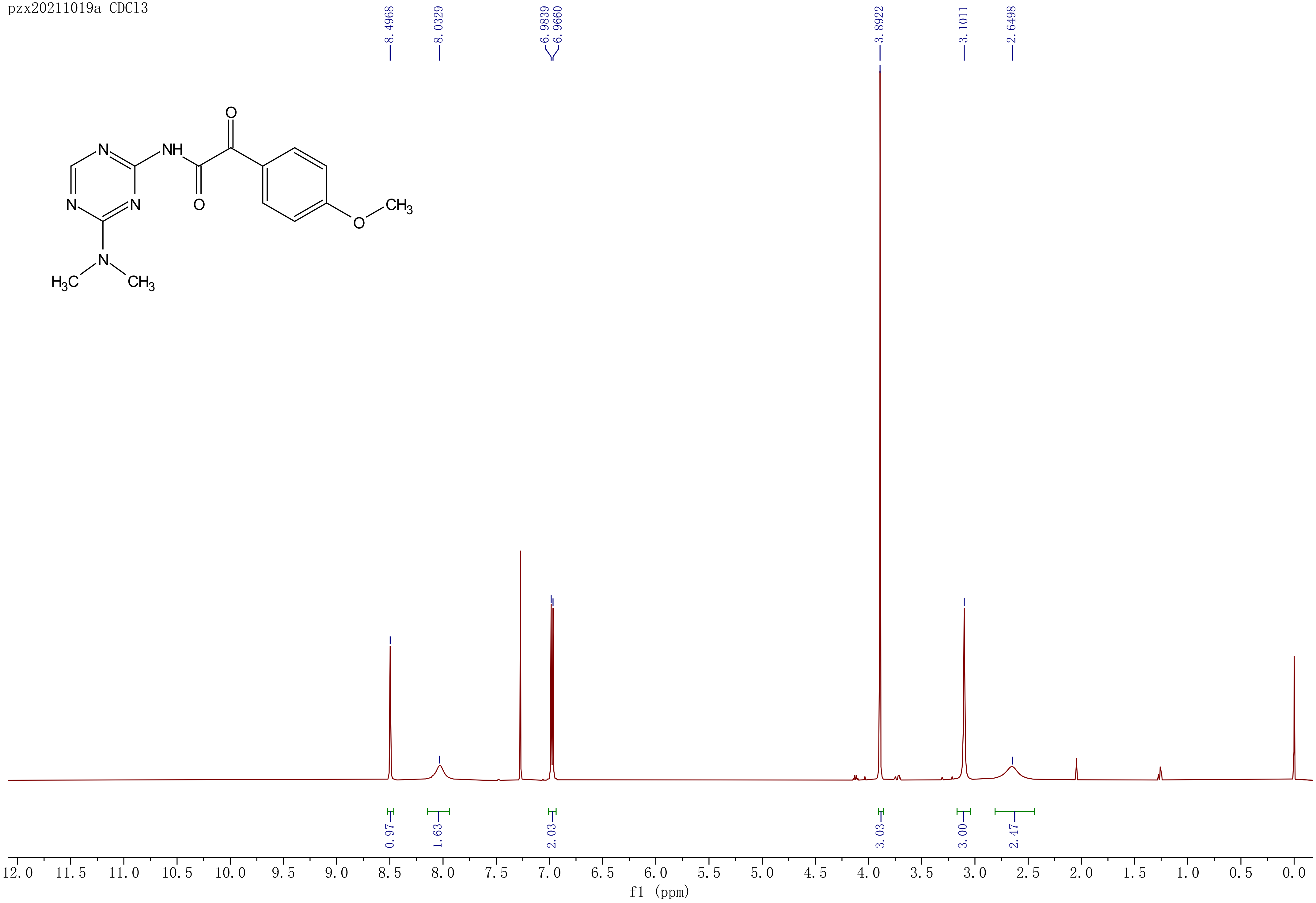

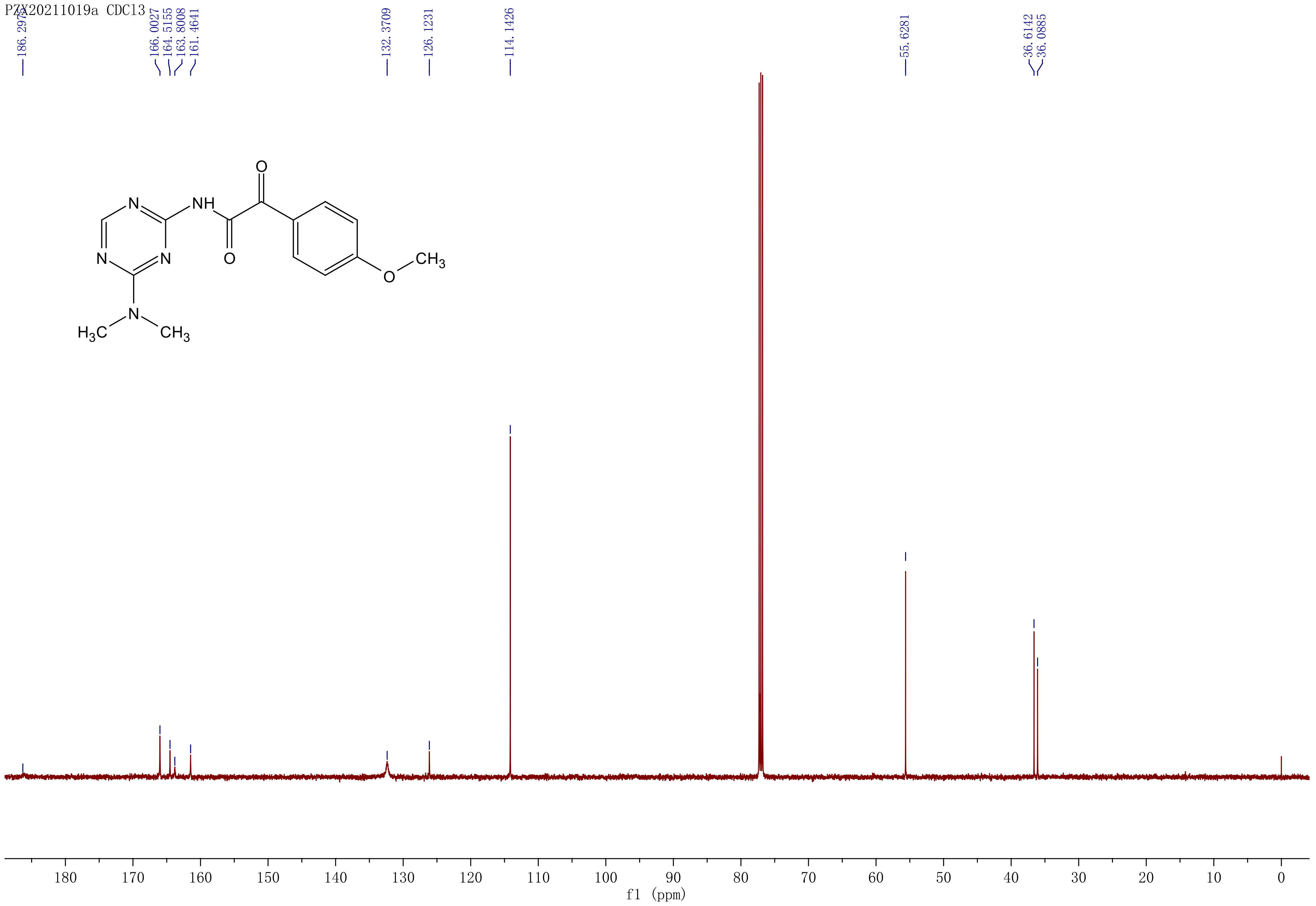
PZX20211019a CDCl3
186.297
166.0027
164.5155
163.8008
161.4641
132.3709
126.1231
114.1426
55.6281
36.6142
36.0885
180
170
160
150
140
130
120
110
100
90
80
70
60
50
40
30
20
10
0
f1 (ppm)

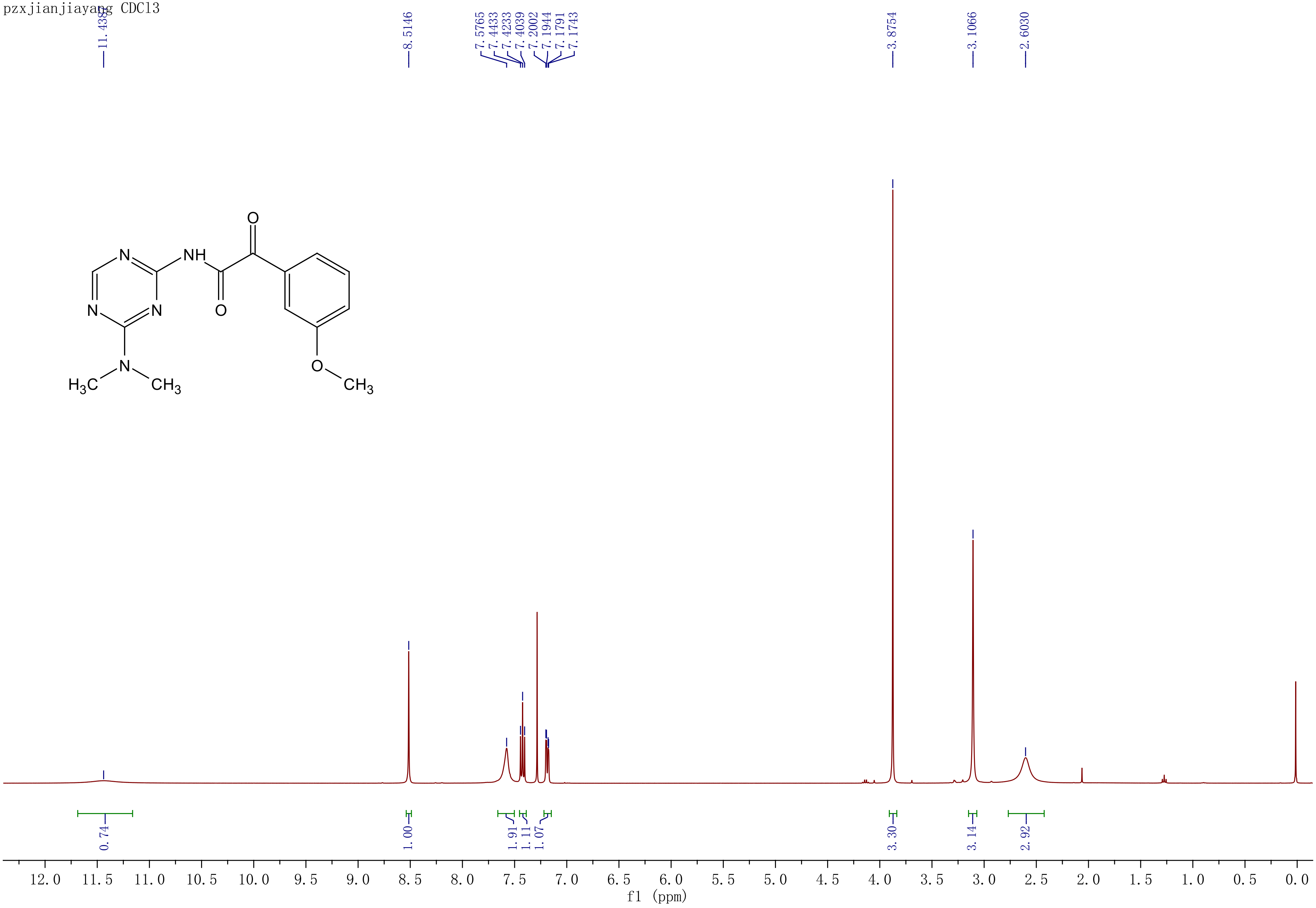
pzxjianjiayang CDCl3
11.4382
8.5146
7.5765
7.4433
7.4233
7.4039
7.2002
7.1944
7.1791
7.1743
3.8754
3.1066
2.6030
N
NH
O
O
N
N
N
H3C
CH3
O
CH3
0.74
1.00
1.91
1.11
1.07
3.30
3.14
2.92
12.0
11.5
11.0
10.5
10.0
9.5
9.0
8.5
8.0
7.5
7.0
6.5
6.0
5.5
5.0
4.5
4.0
3.5
3.0
2.5
2.0
1.5
1.0
0.5
0.0
f1 (ppm)

pzx2022jianjiayang CDCl3

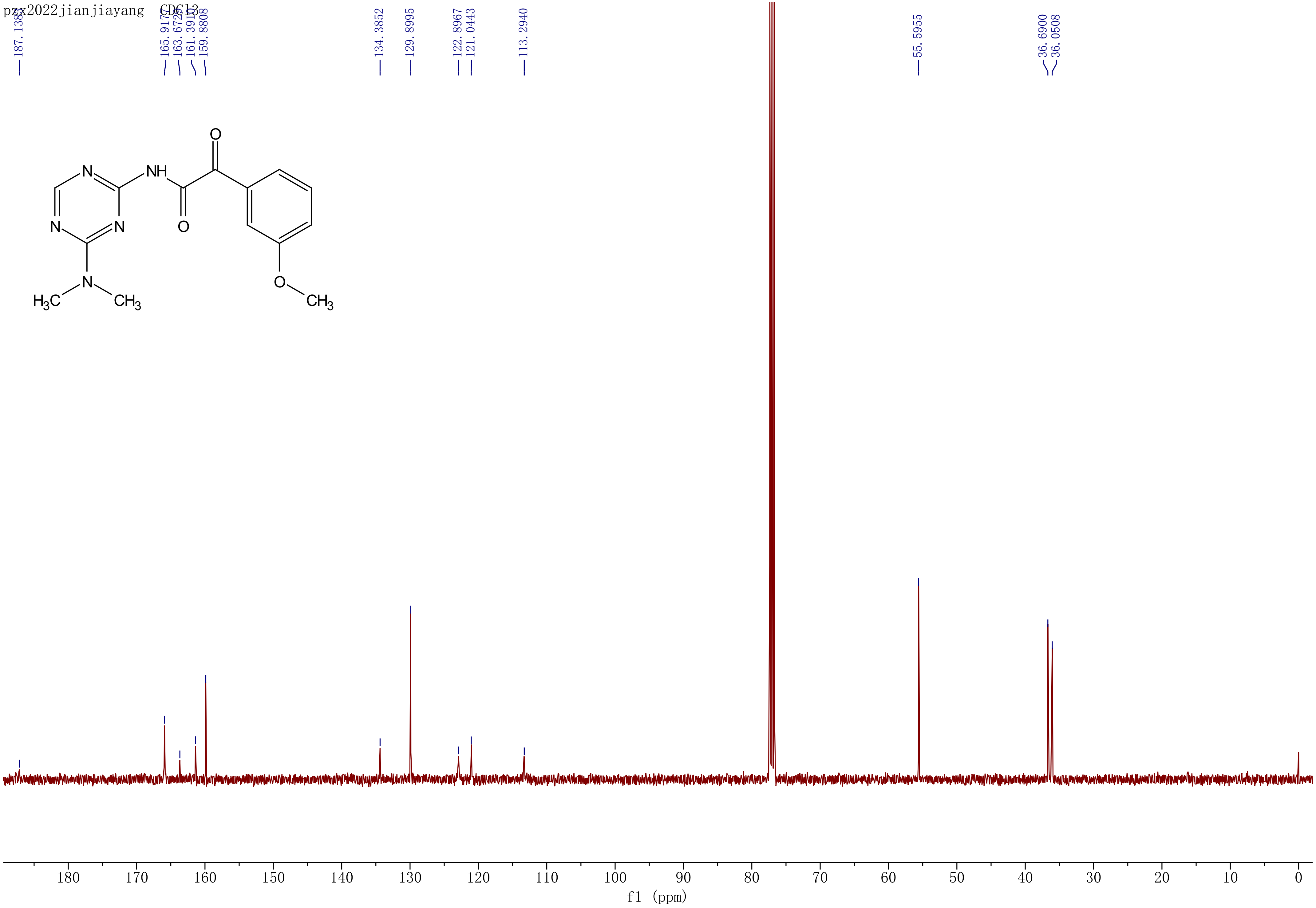

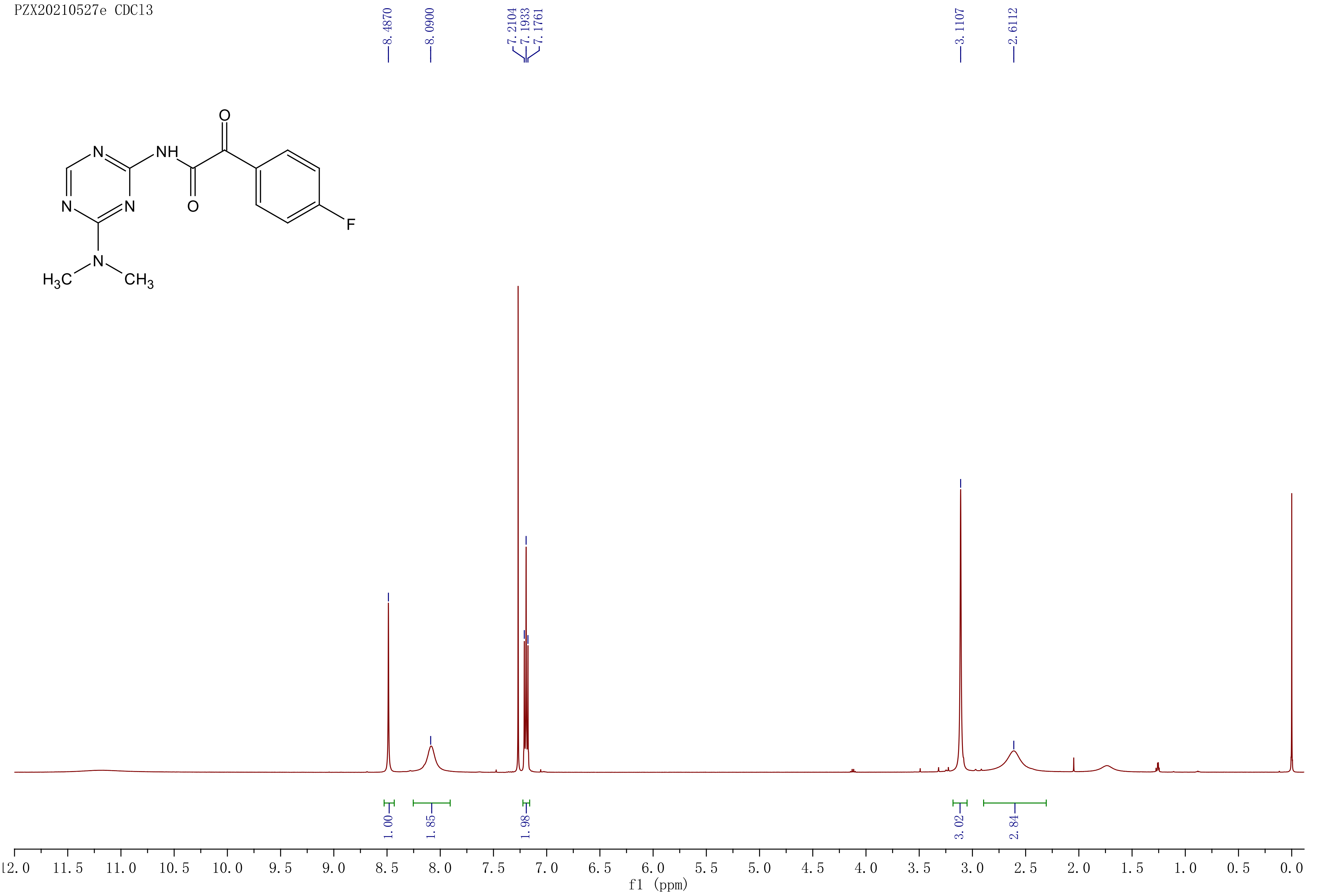

PZX20210527e CDCl3
8.4870
8.0900
7.2104
7.1933
7.1761
3.1107
2.6112
N
NH
O
O
N
N
F
N
H_3C
CH_3
1.00
1.85
1.98
3.02
2.84
12.0
11.5
11.0
10.5
10.0
9.5
9.0
8.5
8.0
7.5
7.0
6.5
6.0
5.5
5.0
4.5
4.0
3.5
3.0
2.5
2.0
1.5
1.0
0.5
0.0
f1 (ppm)

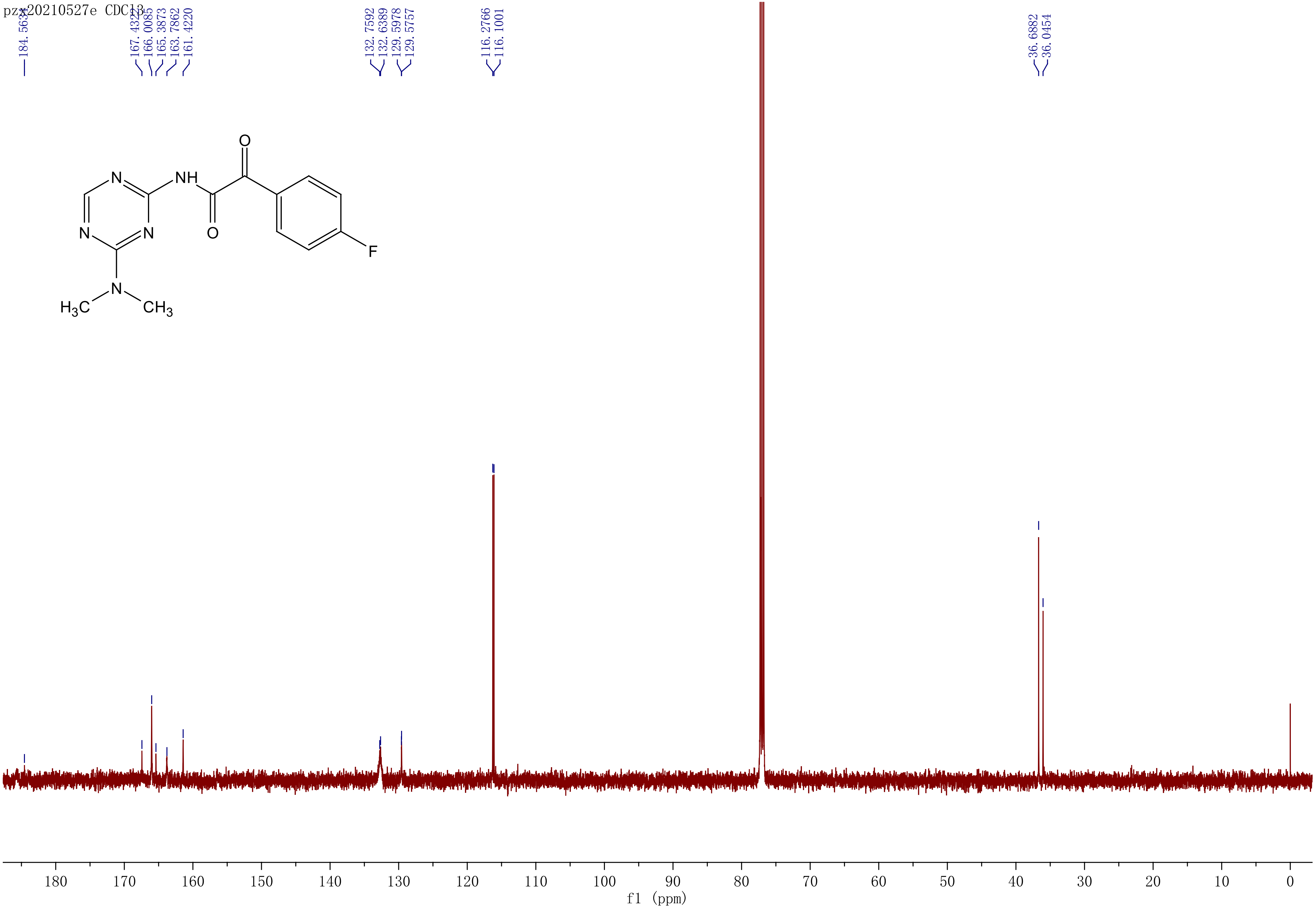
pzx20210527e CDCl3
166.0085
165.3873
163.7862
161.4220
132.7592
132.6389
129.5978
129.5757
116.2766
116.1001
36.6882
36.0454
O
N
NH
N
N
O
F
N
H3C
CH3
180 170 160 150 140 130 120 110 100 90 80 70 60 50 40 30 20 10 0
f1 (ppm)

pzx20220327 CDCl3

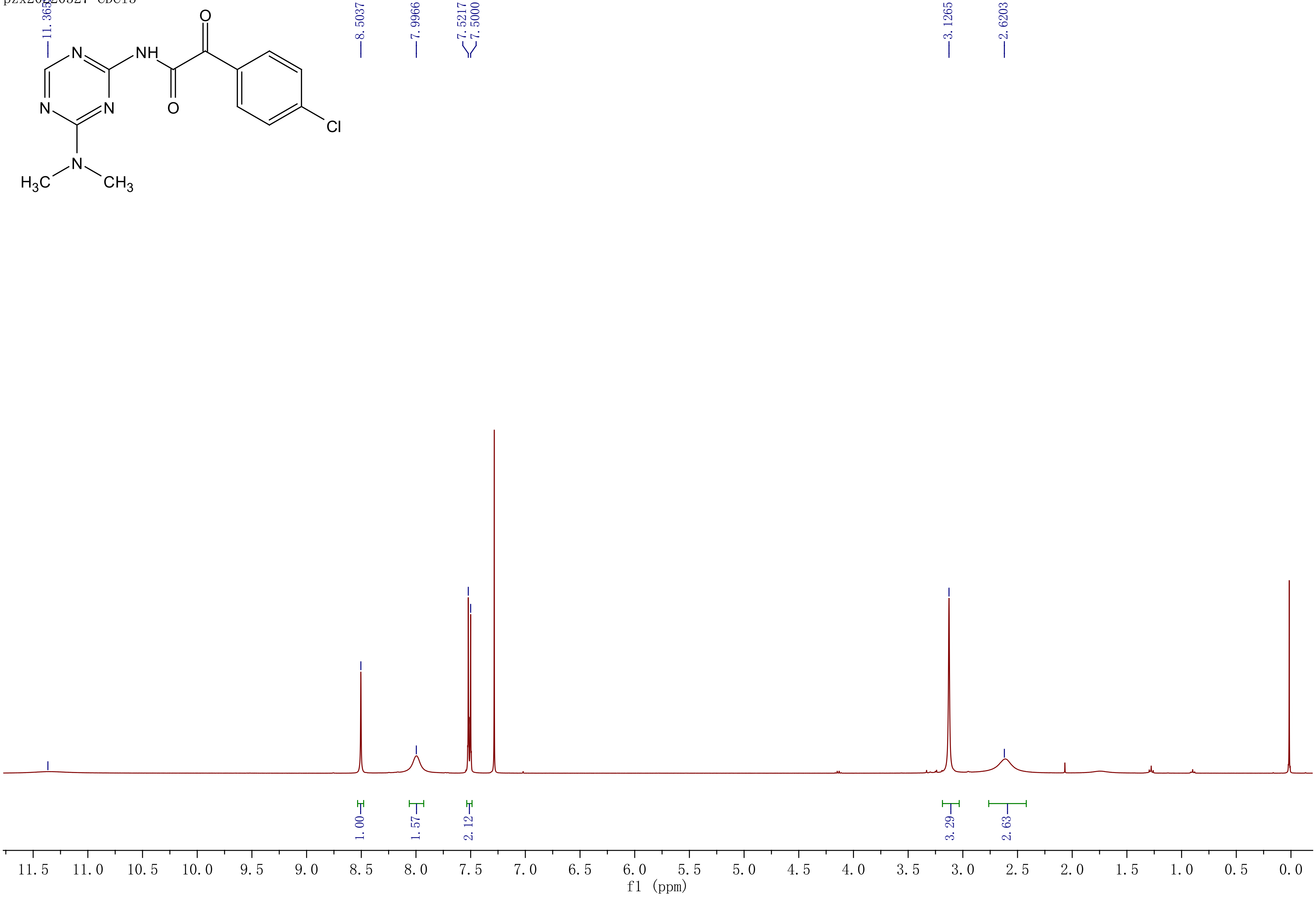

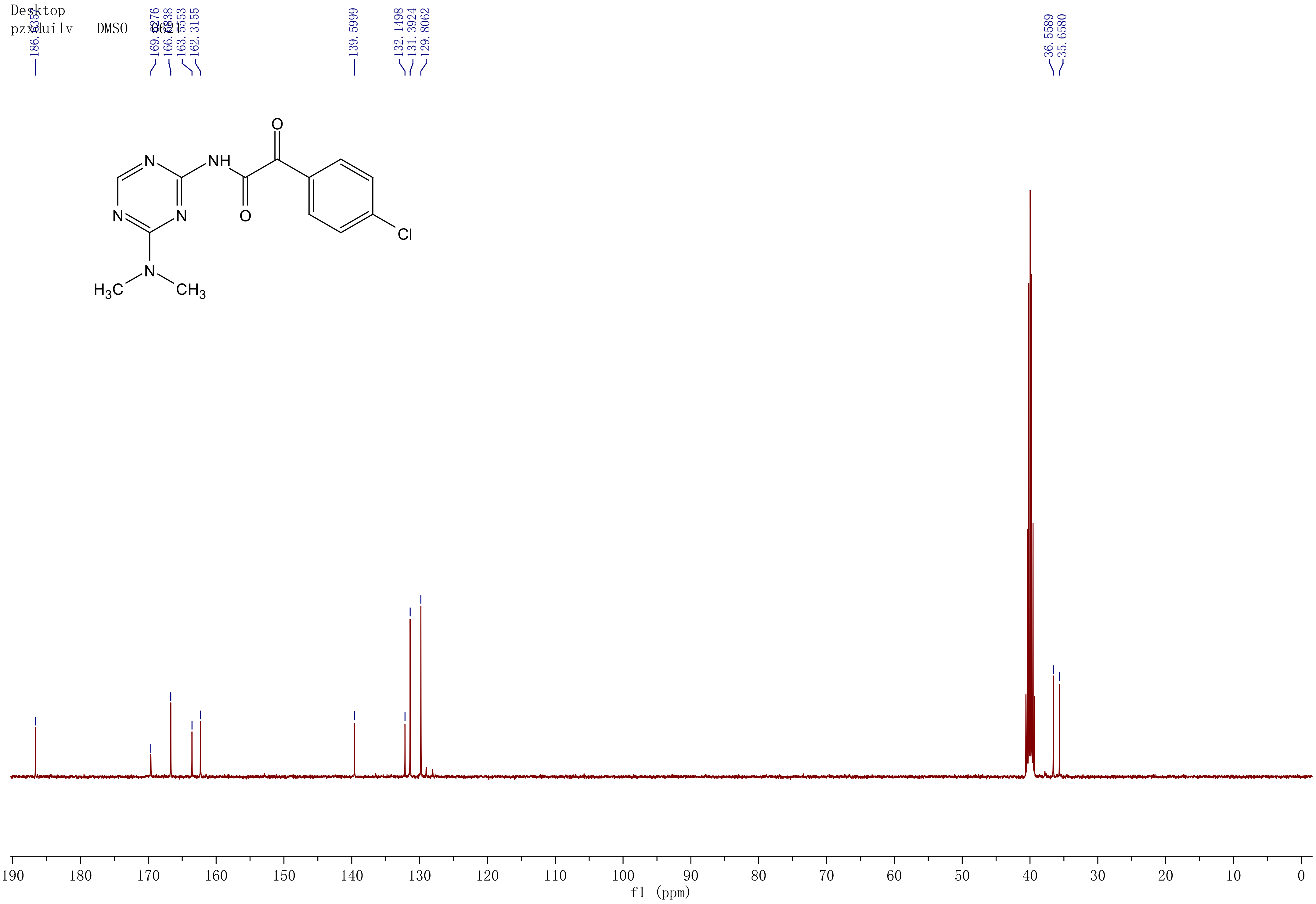
Desktop
pzxduilv DMSO
162.3155
139.5999
132.1498
131.3924
129.8062
36.5589
35.6580
O
NH
N
N
N
O
Cl
N
H3C
CH3
190 180 170 160 150 140 130 120 110 100 90 80 70 60 50 40 30 20 10 0
f1 (ppm)

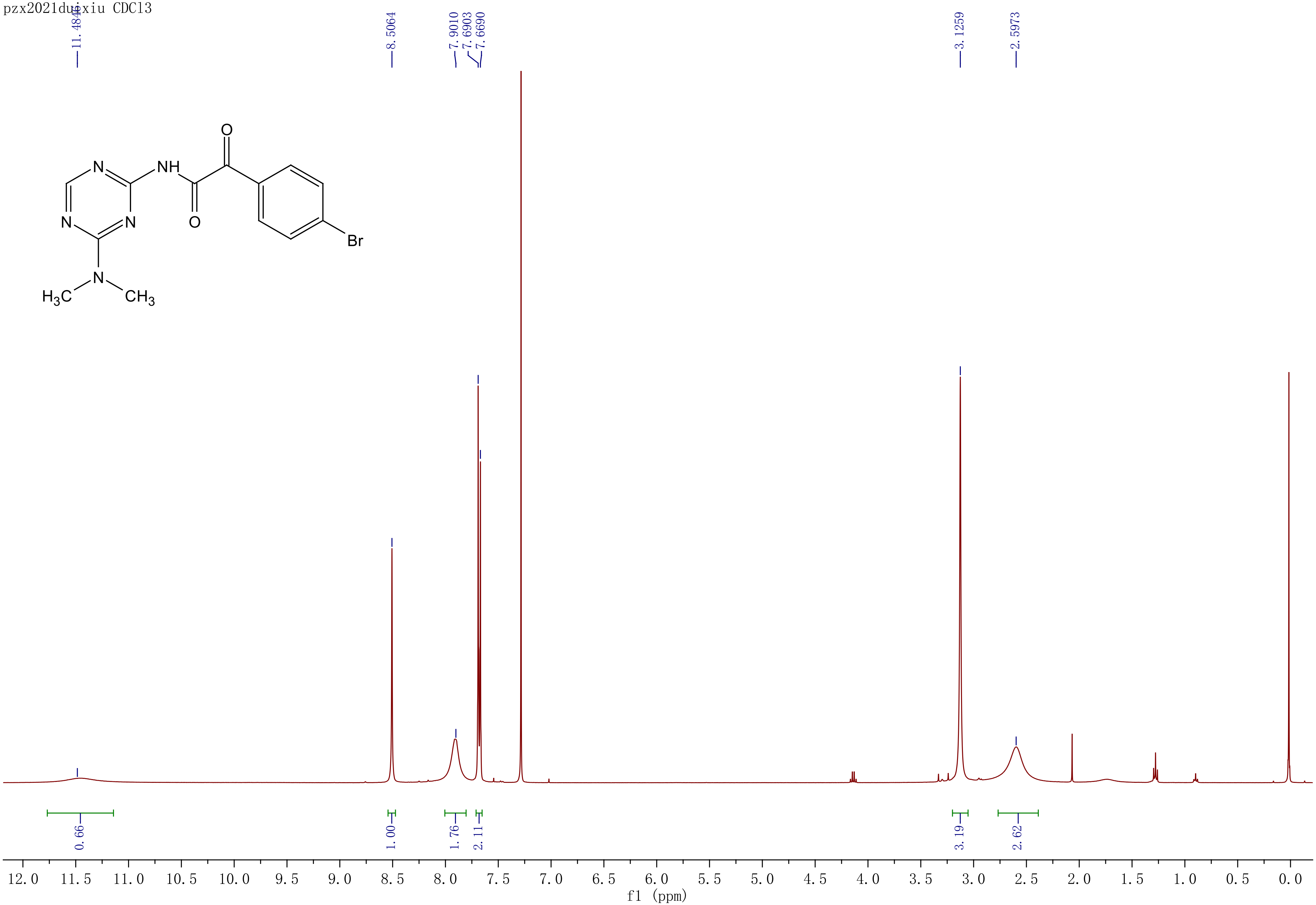
pzx2021duixiu CDCl3
11.4848
8.5064
7.9010
7.6903
7.6690
3.1259
2.5973
N
NH
O
O
N
N
Br
N
H3C
CH3
0.66
1.00
1.76
2.11
3.19
2.62
12.0
11.5
11.0
10.5
10.0
9.5
9.0
8.5
8.0
7.5
7.0
6.5
6.0
5.5
5.0
4.5
4.0
3.5
3.0
2.5
2.0
1.5
1.0
0.5
0.0
f1 (ppm)

Desktop
pzx2021duixiu CDCl3 0221

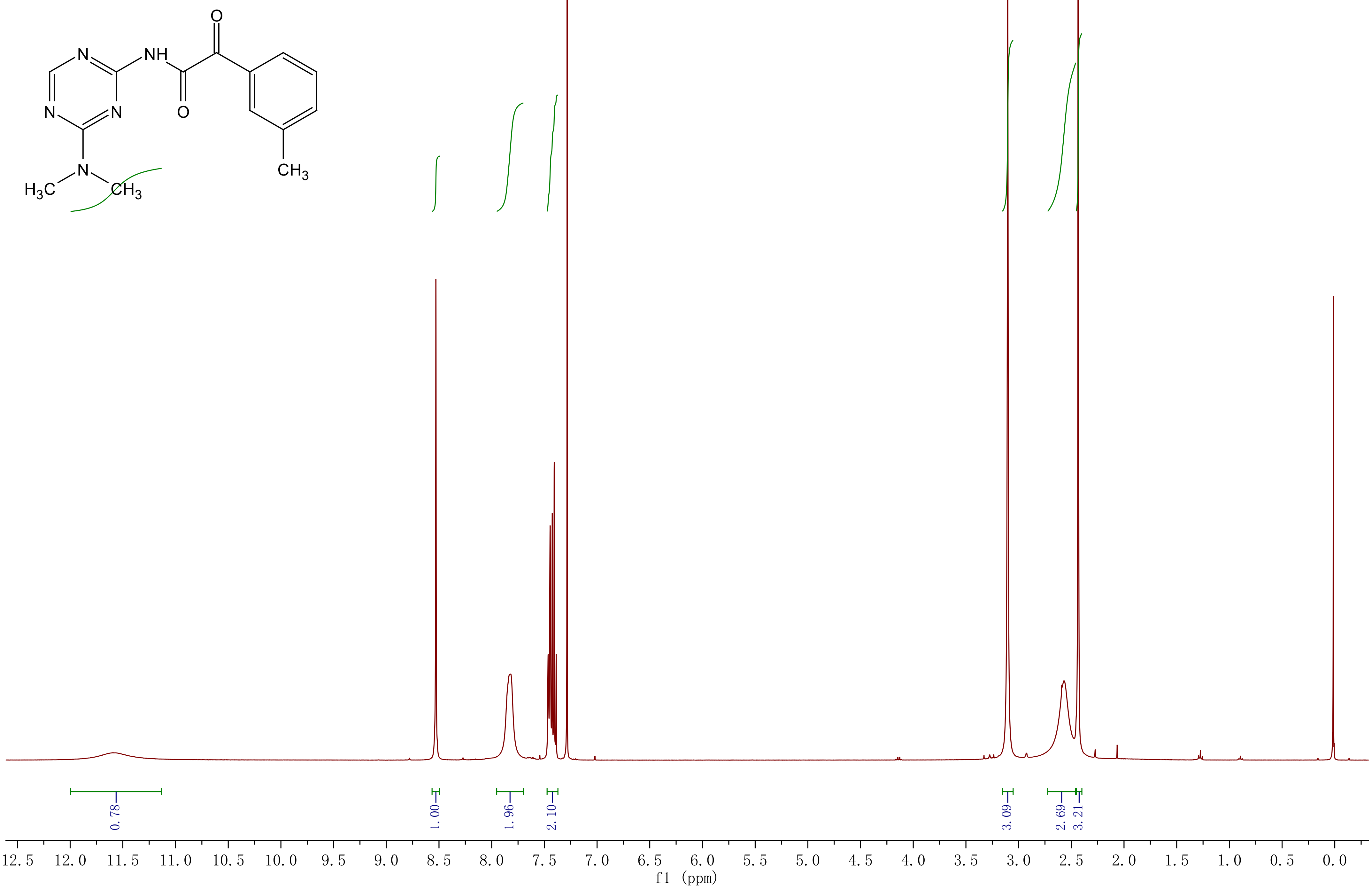

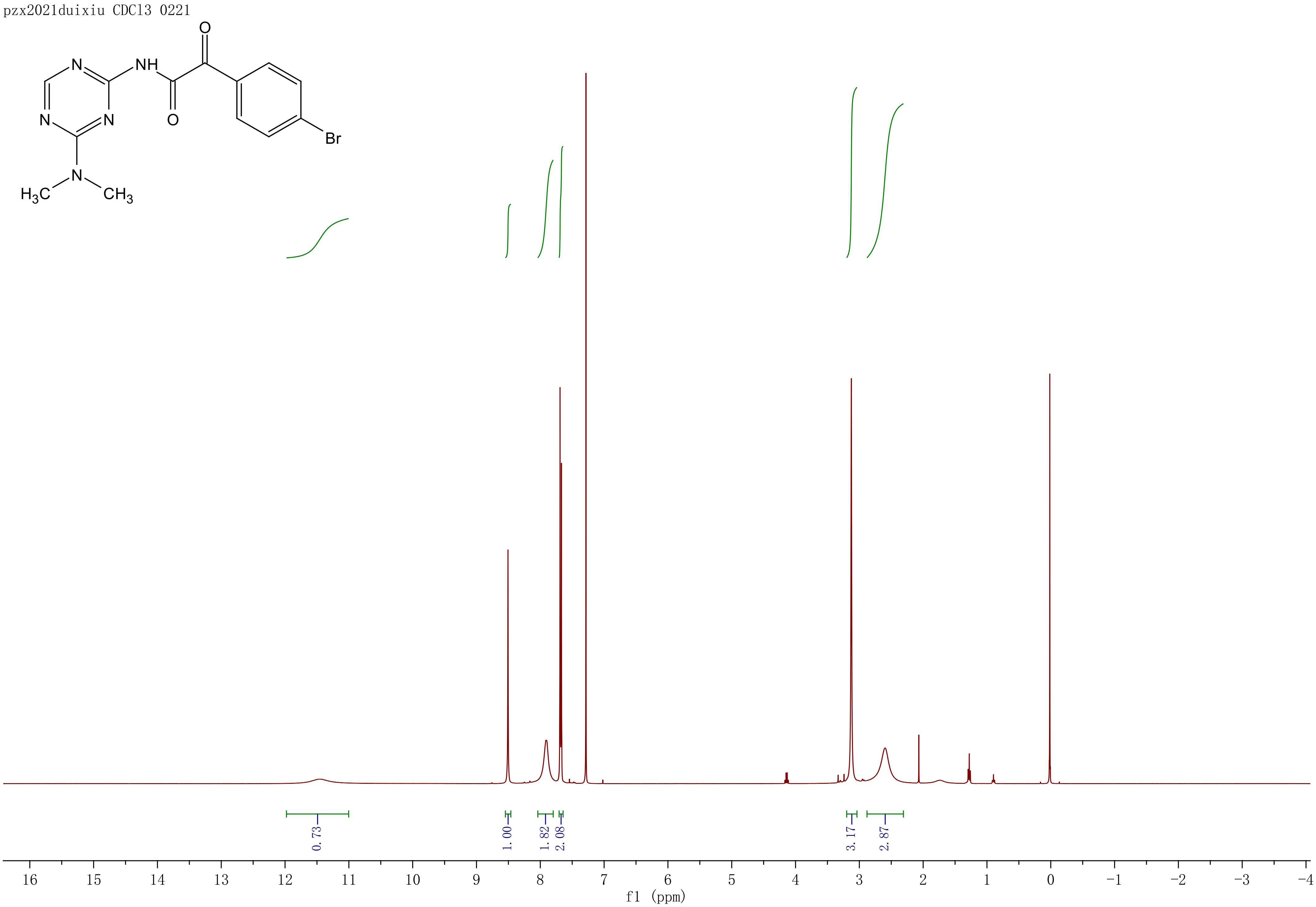
pzx2021duixiu CDCl3 0221
O
N
NH
N
N
O
Br
N
H3C
CH3
0.73
1.00
1.82
2.08
3.17
2.87
16 15 14 13 12 11 10 9 8 7 6 5 4 3 2 1 0 −1 −2 −3 −4
f1 (ppm)

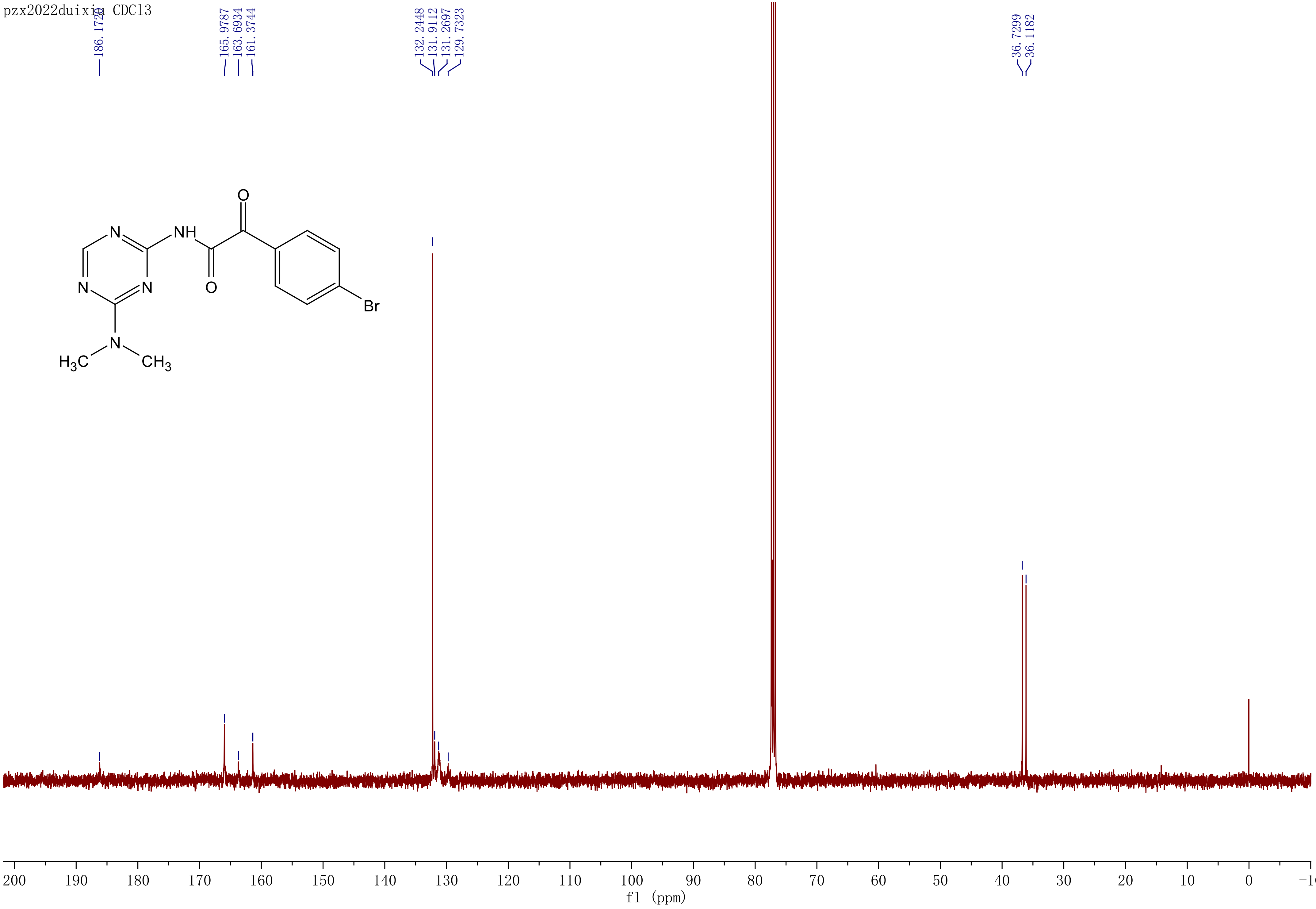

pzx2022duixiu CDCl3
186.1720
165.9787
163.6934
161.3744
132.2448
131.9112
131.2697
129.7323
36.7299
36.1182
N
N
N
NH
O
O
Br
N
H3C
CH3
200 190 180 170 160 150 140 130 120 110 100 90 80 70 60 50 40 30 20 10 0 −10
f1 (ppm)

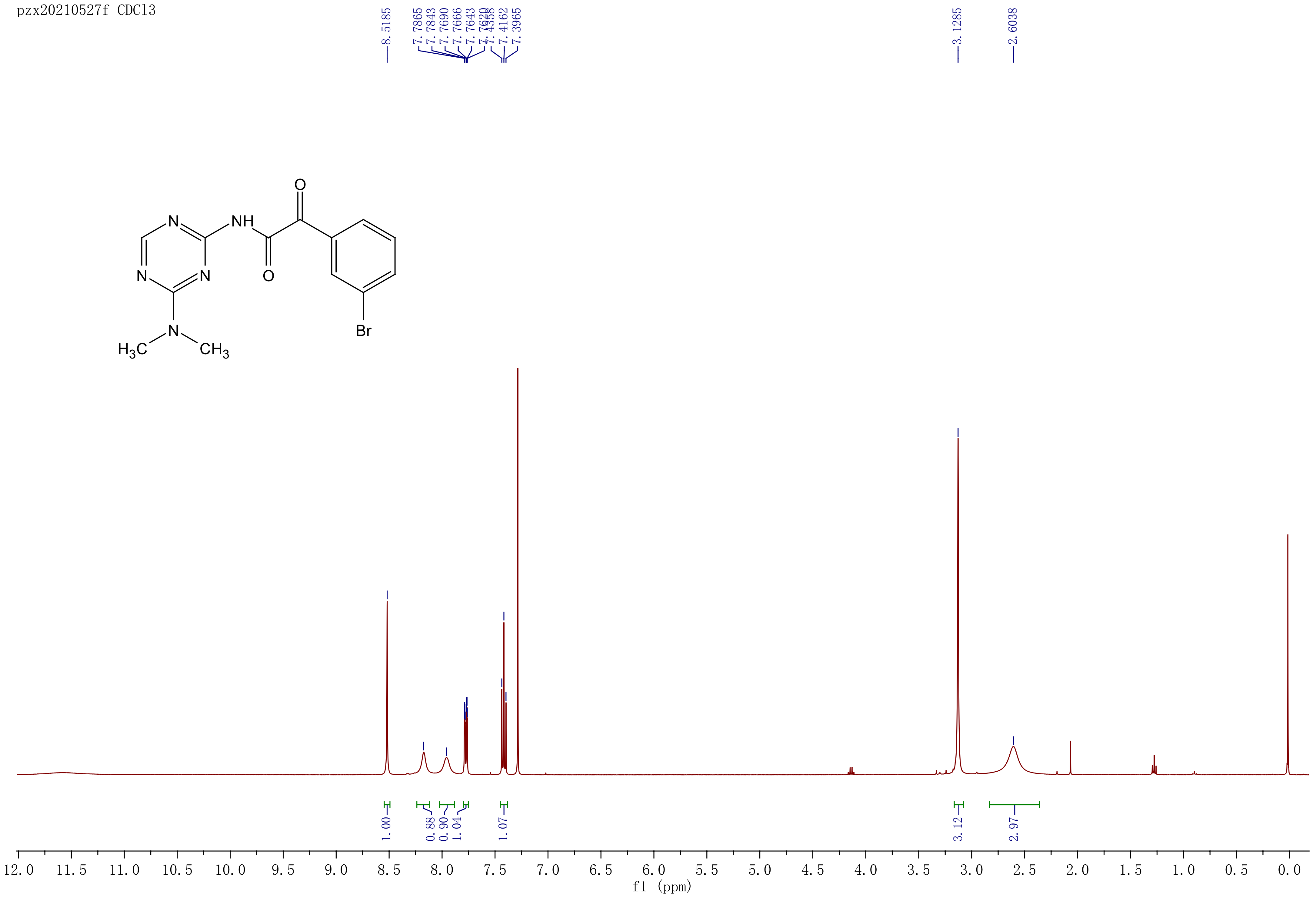

pzx20210527f CDCl3
8.5185
7.7865
7.7843
7.7690
7.7666
7.7643
7.7620
7.4358
7.4162
7.3965
3.1285
2.6038
N
NH
O
N
N
O
N
H_3C
CH_3
Br
1.00
0.88
0.90
1.04
1.07
3.12
2.97
f1 (ppm)

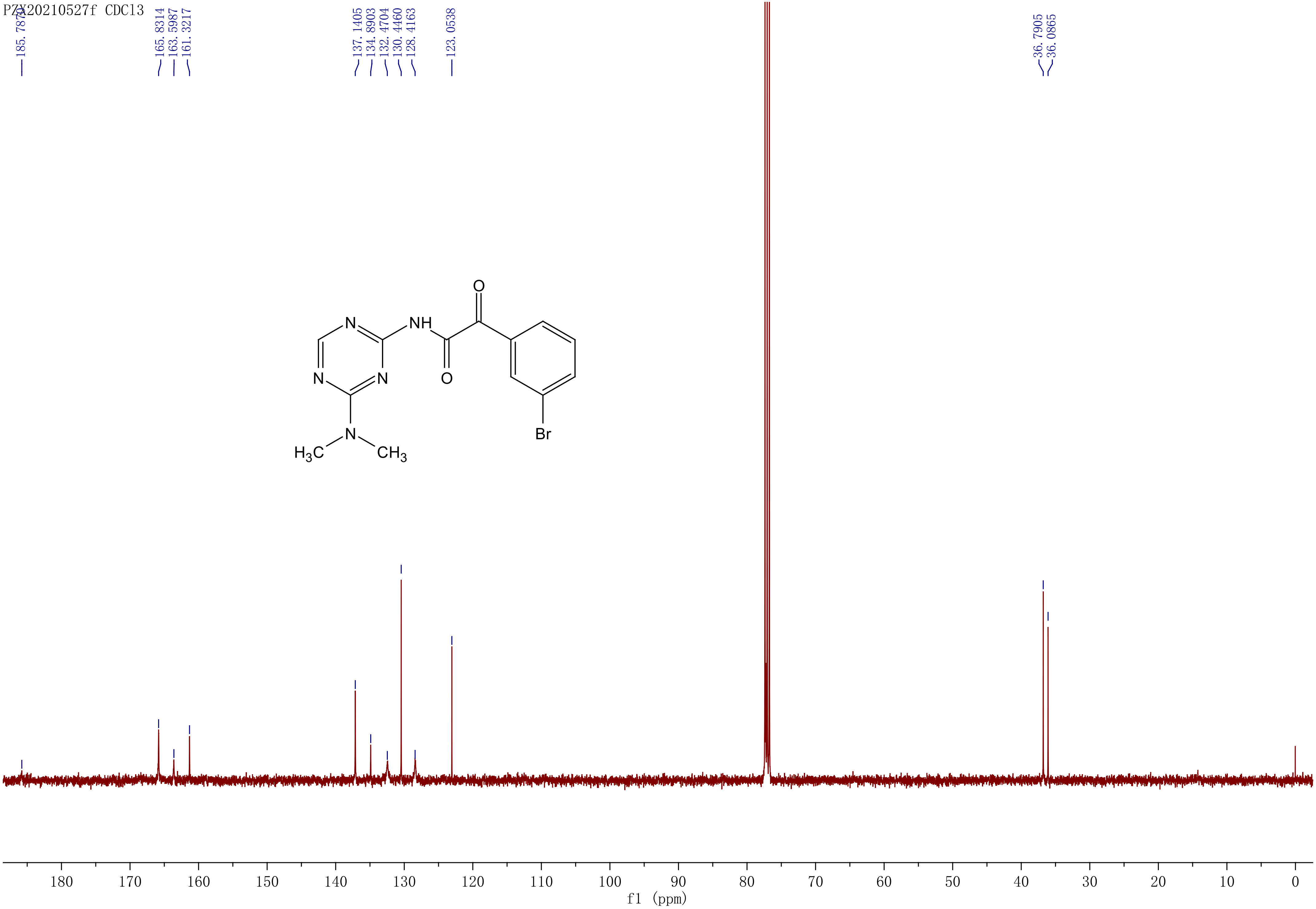
PZY20210527f CDCl3
185.7870
165.8314
163.5987
161.3217
137.1405
134.8903
132.4704
130.4460
128.4163
123.0538
36.7905
36.0865
O
N
NH
N
N
O
N
H3C
CH3
Br
180
170
160
150
140
130
120
110
100
90
80
70
60
50
40
30
20
10
0
f1 (ppm)

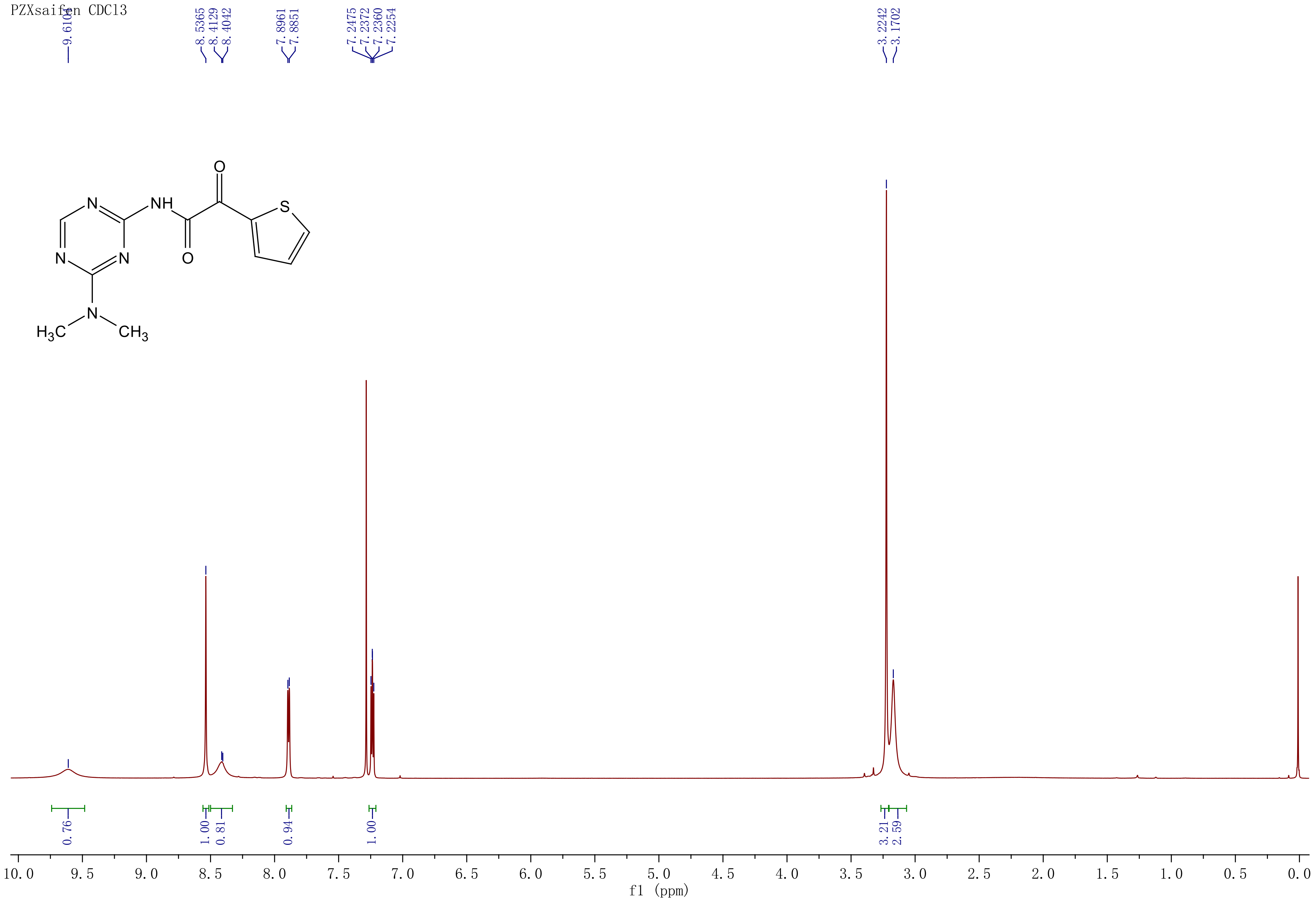

PZXsaifen CDCl3
9.6104
8.5365
8.4129
8.4042
7.8961
7.8851
7.2475
7.2372
7.2360
7.2254
3.2242
3.1702
O
N
NH
S
N
N
O
N
H3C
CH3
0.76
1.00
0.81
0.94
1.00
3.21
2.59
10.0 9.5 9.0 8.5 8.0 7.5 7.0 6.5 6.0 5.5 5.0 4.5 4.0 3.5 3.0 2.5 2.0 1.5 1.0 0.5 0.0
f1 (ppm)

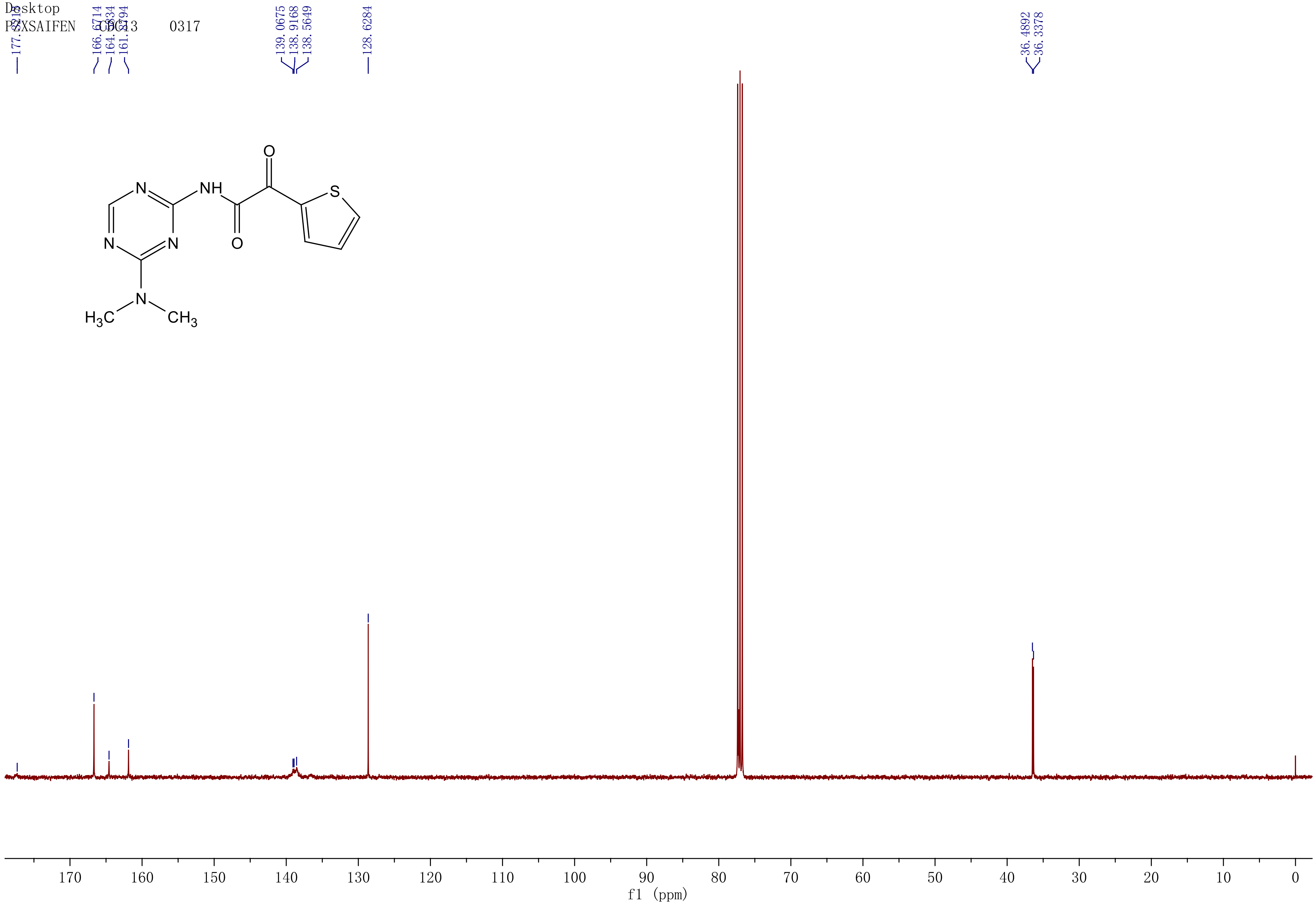

Desktop
PZXSAIFEN CDCl3 0317
177.3213
166.6714
164.5834
161.8794
139.0675
138.9168
138.5649
128.6284
36.4892
36.3378
O
NH
S
N
N
N
O
N
H3C
CH3
170
160
150
140
130
120
110
100
90
80
70
60
50
40
30
20
10
0
f1 (ppm)

PZX20211121 CDCl3

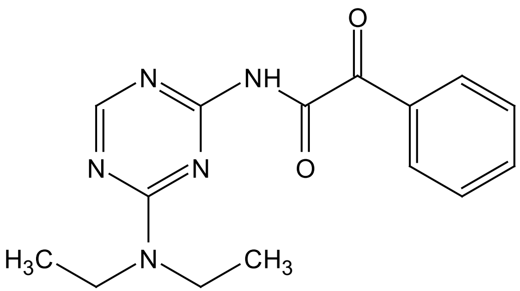

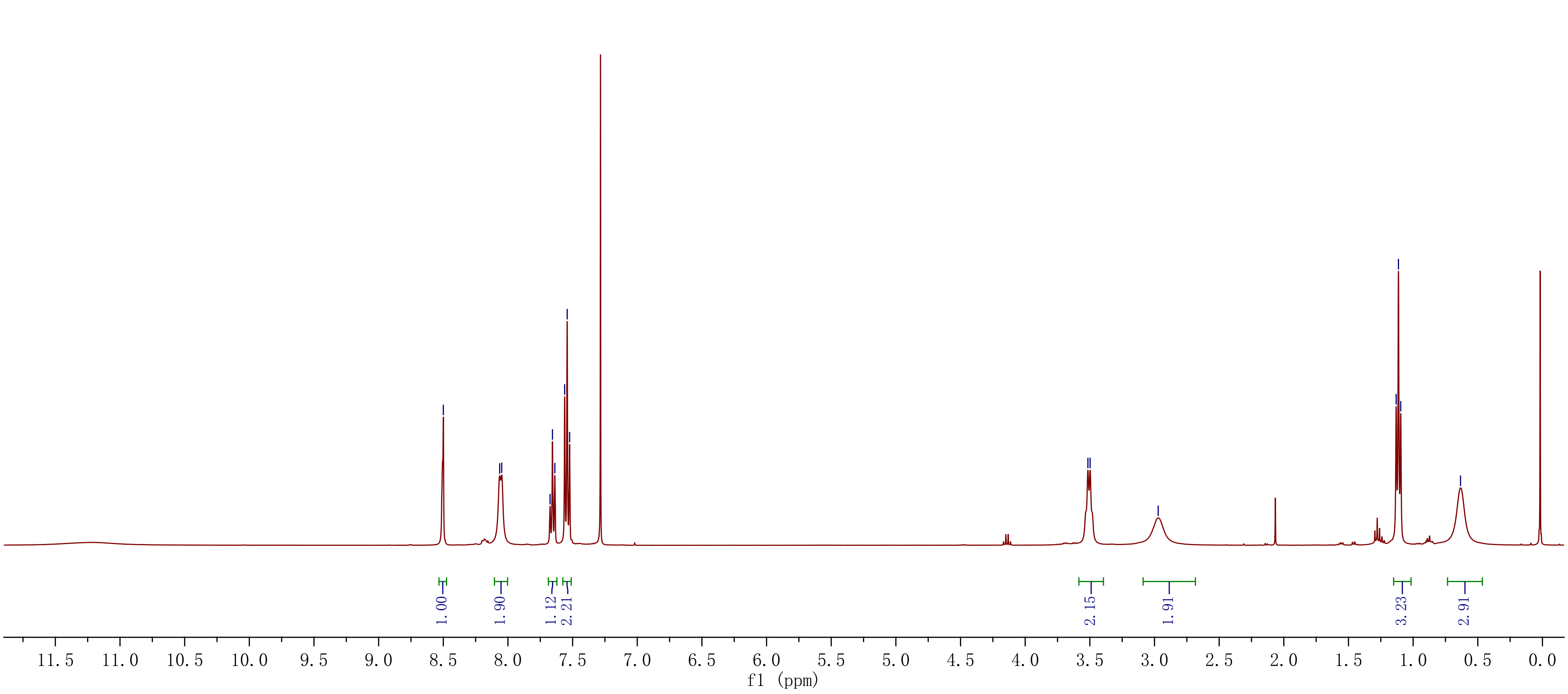

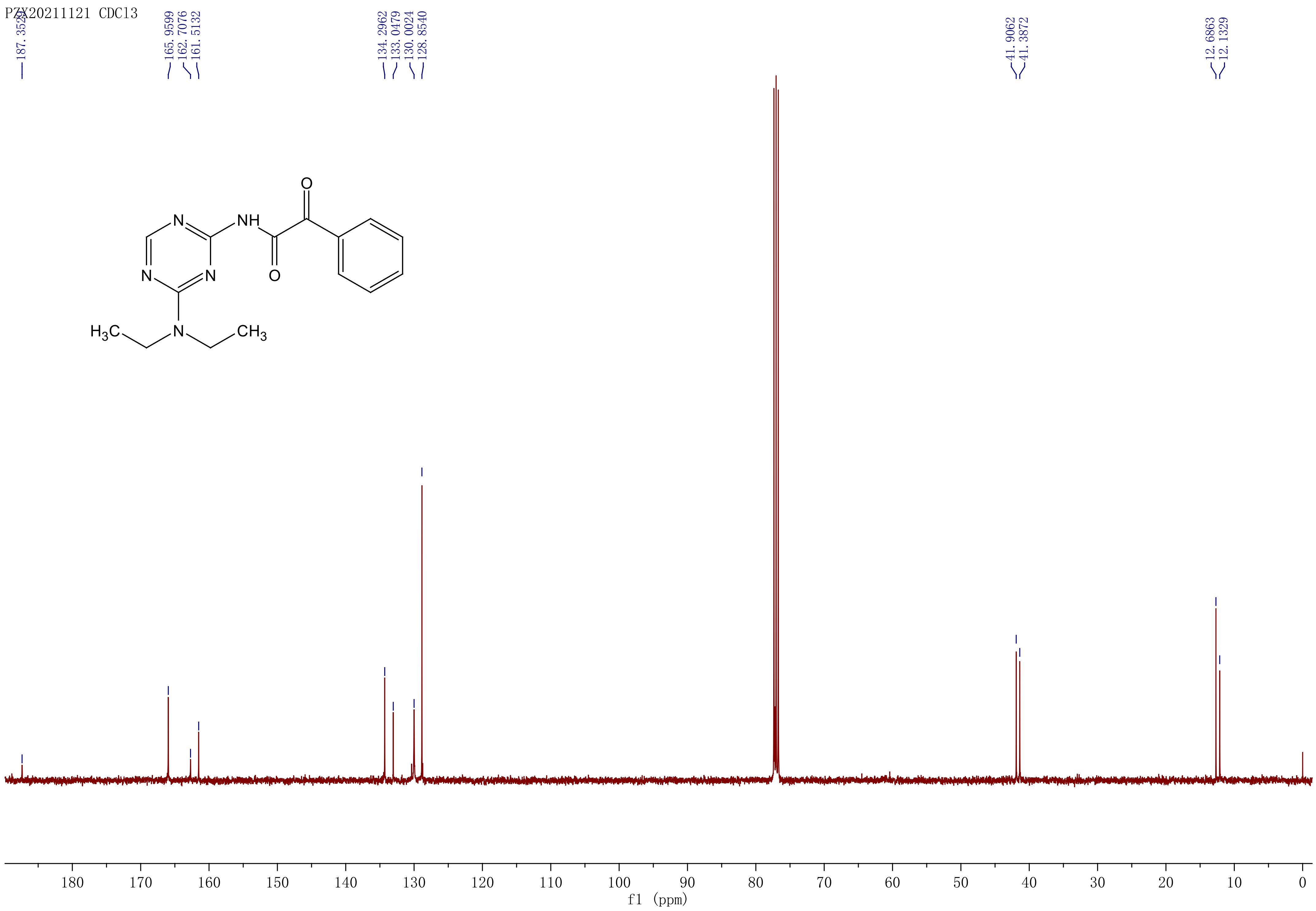
PZX20211121 CDCl3
187.352
165.9599
162.7076
161.5132
134.2962
133.0479
130.0024
128.8540
41.9062
41.3872
12.6863
12.1329
O
N
NH
N
N
O
H_3C
N
CH_3
180 170 160 150 140 130 120 110 100 90 80 70 60 50 40 30 20 10 0
f1 (ppm)

PZX20210526b CDCl3

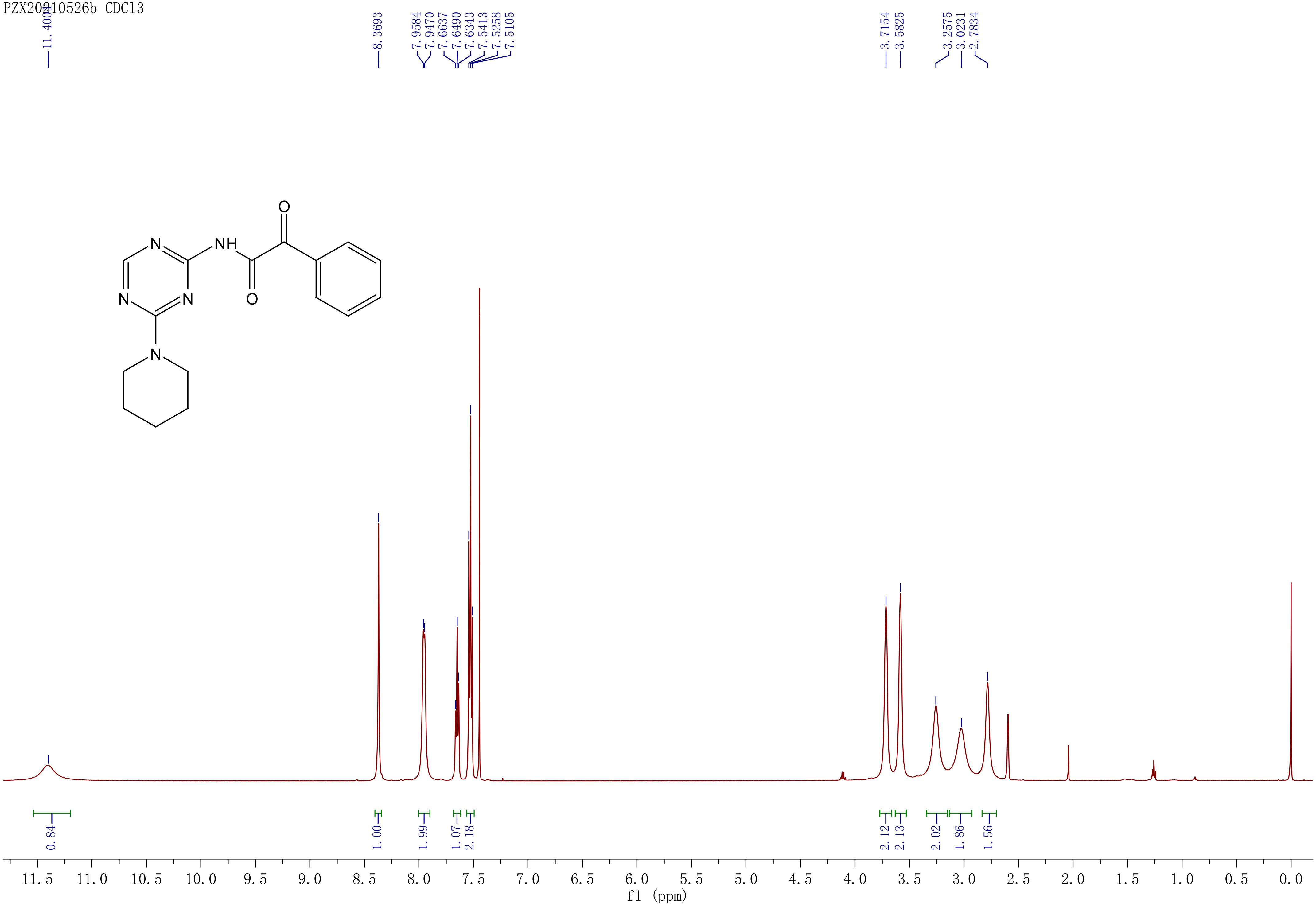

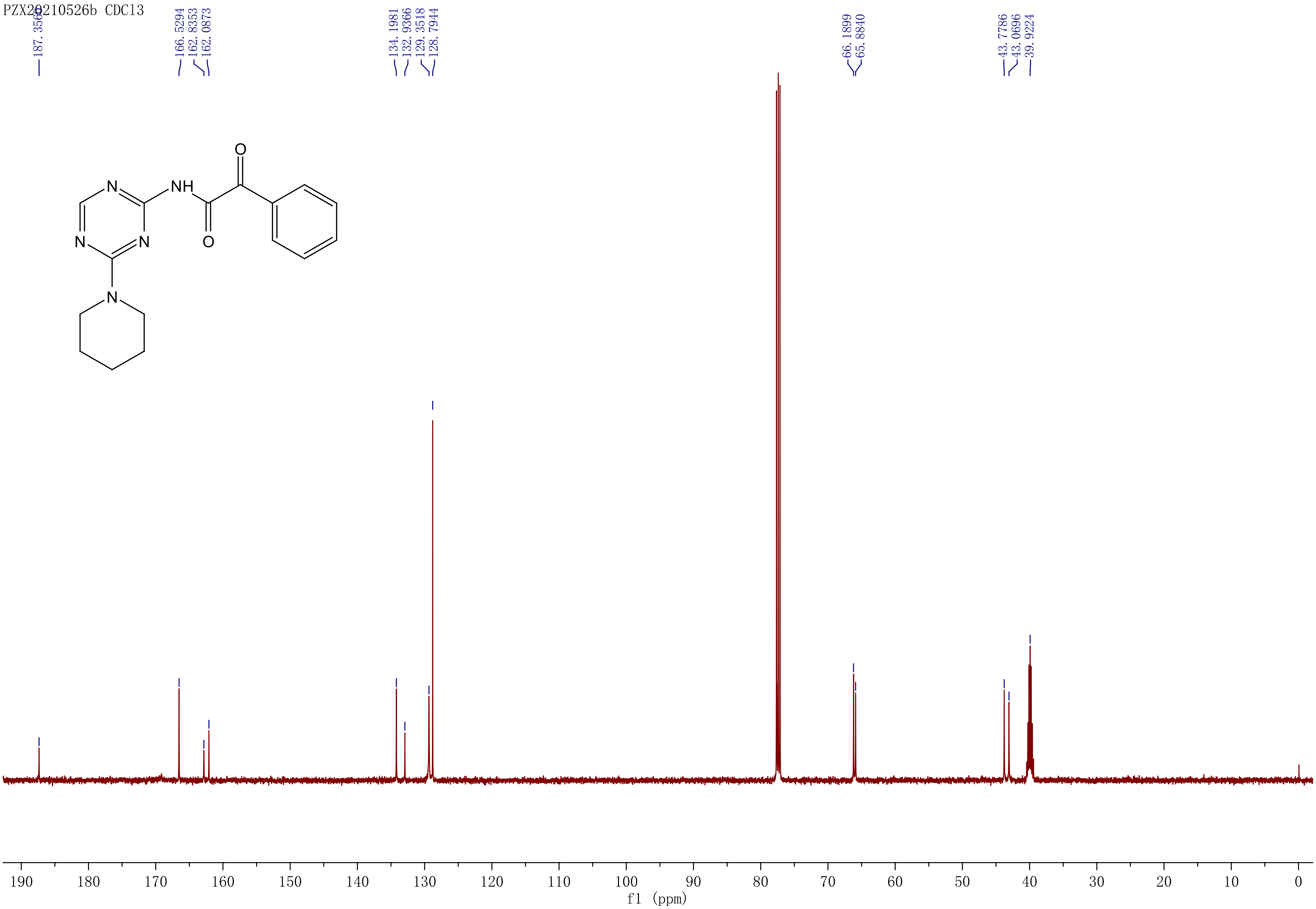

PZX20210526b CDCl3
187.356
166.5294
162.8353
162.0873
134.1981
132.9366
129.3518
128.7944
66.1899
65.8840
43.7786
43.0696
39.9224
O
N
NH
N
N
O
N
190
180
170
160
150
140
130
120
110
100
90
80
70
60
50
40
30
20
10
0
f1 (ppm)

PZX20210526a CDCl3

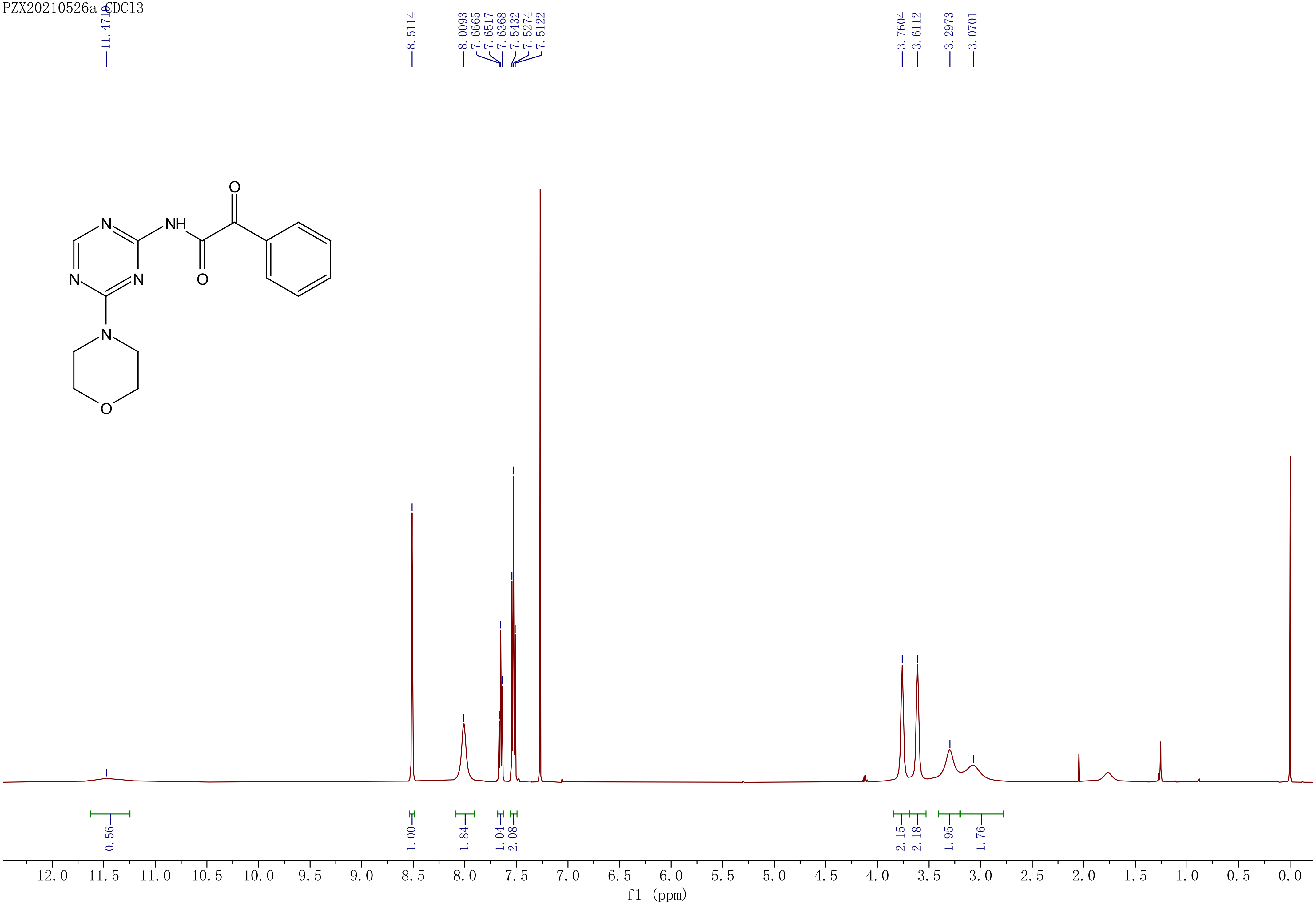

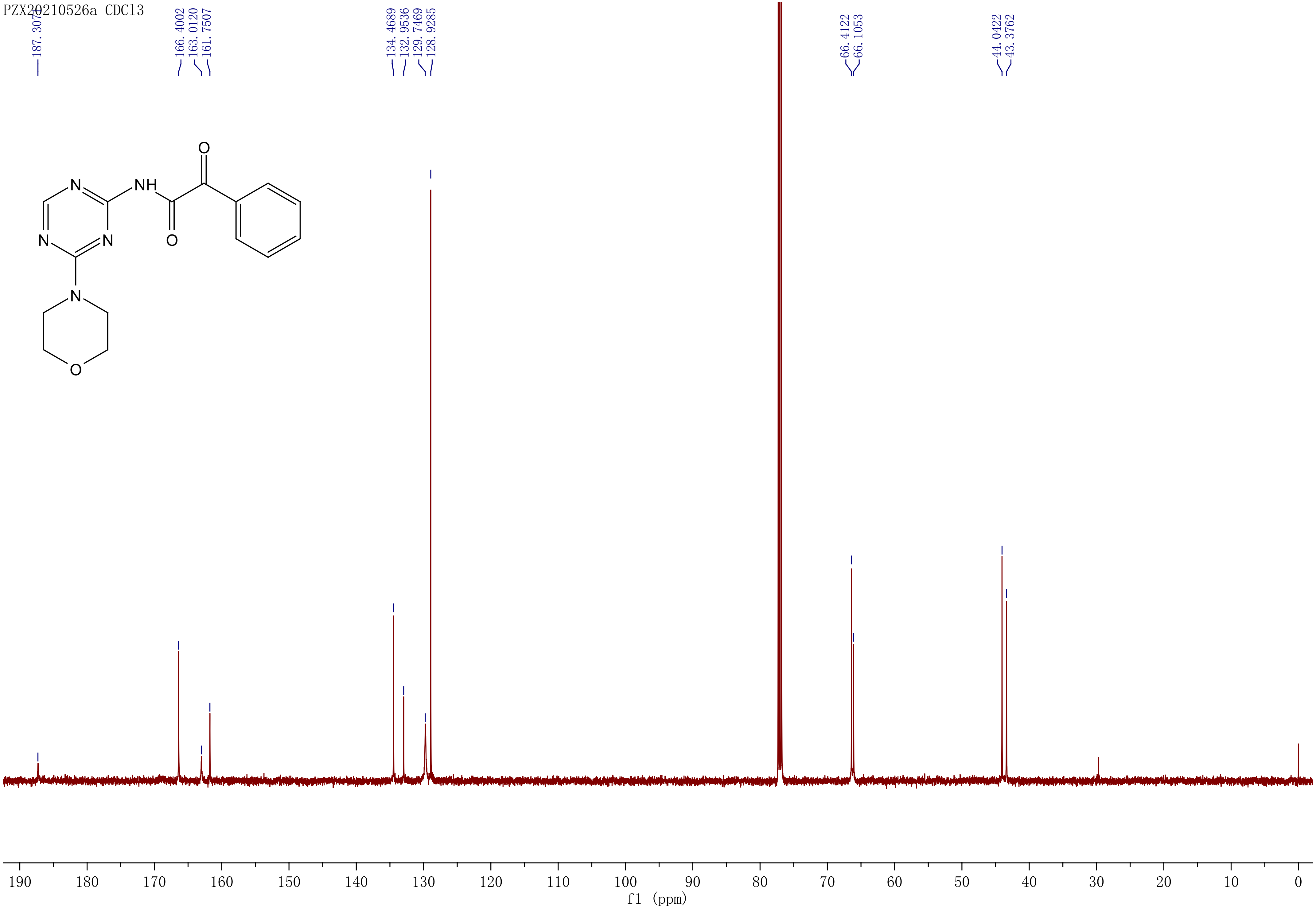

PZX20210526a CDCl3
187.307
166.4002
163.0120
161.7507
134.4689
132.9536
129.7469
128.9285
66.4122
66.1053
44.0422
43.3762
O
NH
N
N
N
O
N
O
190 180 170 160 150 140 130 120 110 100 90 80 70 60 50 40 30 20 10 0
f1 (ppm)

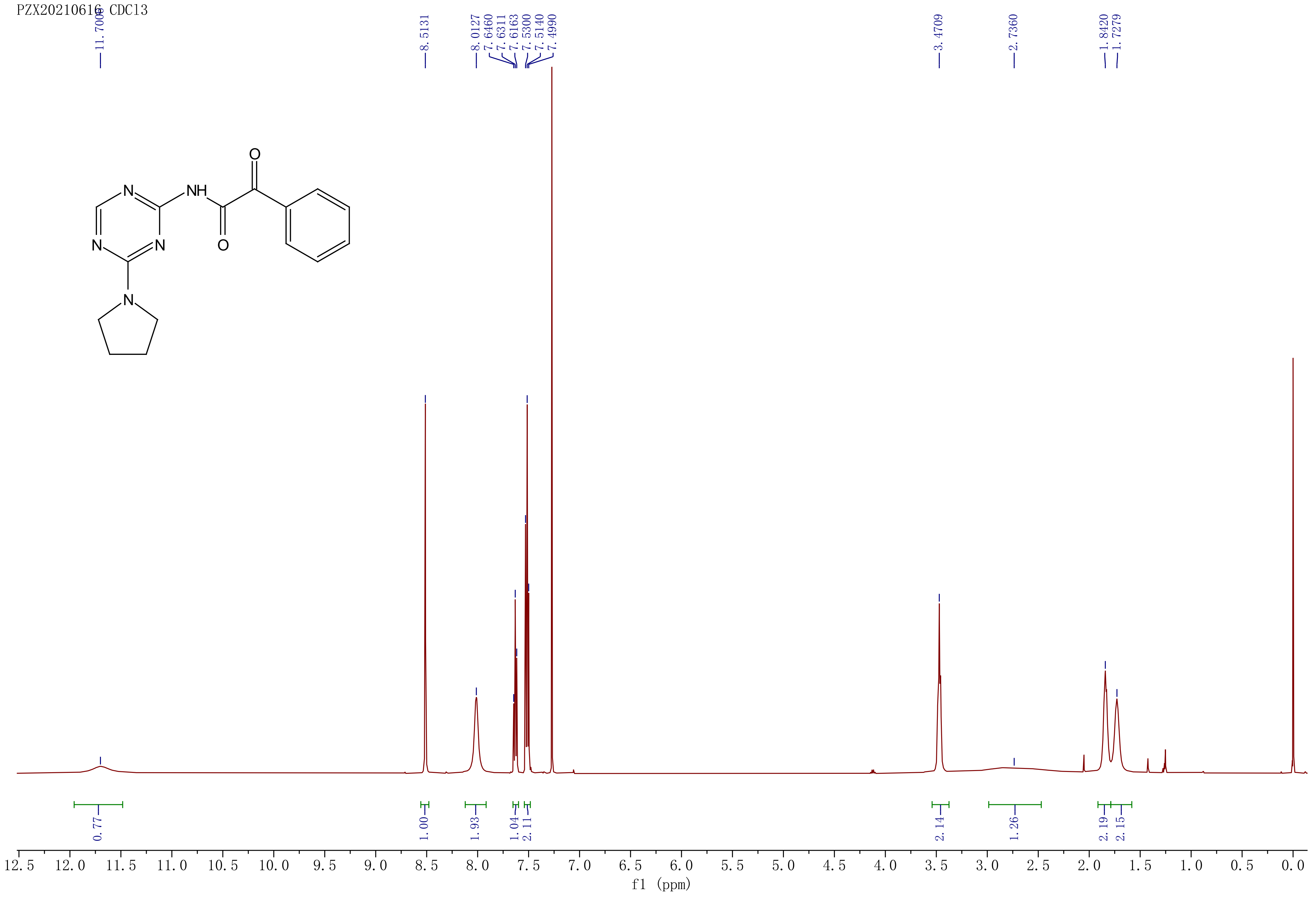
PZX20210616 CDCl3
11.7008
8.5131
8.0127
7.6460
7.6311
7.6163
7.5300
7.5140
7.4990
3.4709
2.7360
1.8420
1.7279
NH
O
N
0.77
1.00
1.93
1.04
2.11
2.14
1.26
2.19
2.15
12.5 12.0 11.5 11.0 10.5 10.0 9.5 9.0 8.5 8.0 7.5 7.0 6.5 6.0 5.5 5.0 4.5 4.0 3.5 3.0 2.5 2.0 1.5 1.0 0.5 0.0
f1 (ppm)

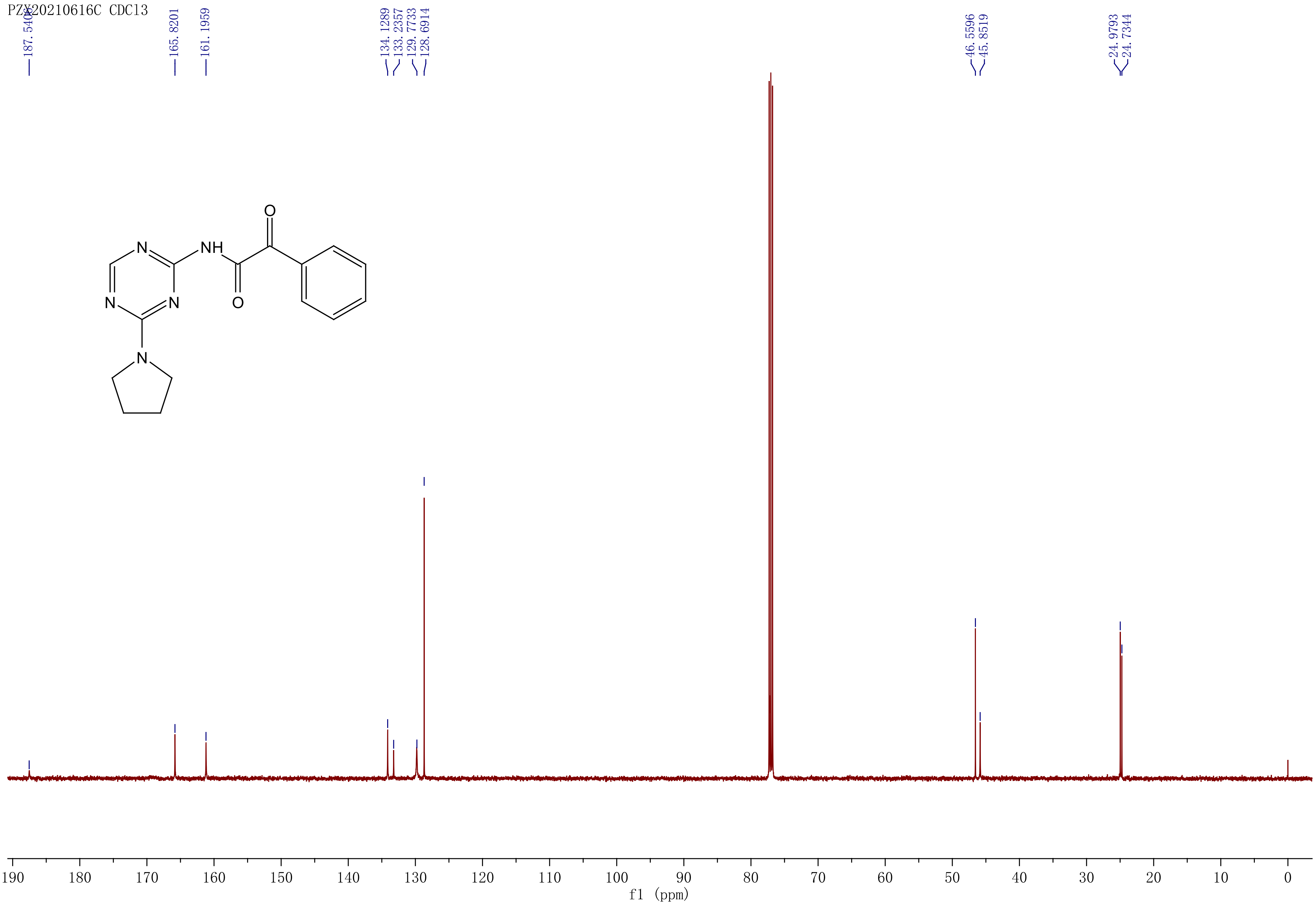
PZY20210616C CDCl3
187.5408
165.8201
161.1959
134.1289
133.2357
129.7733
128.6914
46.5596
45.8519
24.9793
24.7344
N
NH
O
N
N
O
N
190 180 170 160 150 140 130 120 110 100 90 80 70 60 50 40 30 20 10 0
f1 (ppm)

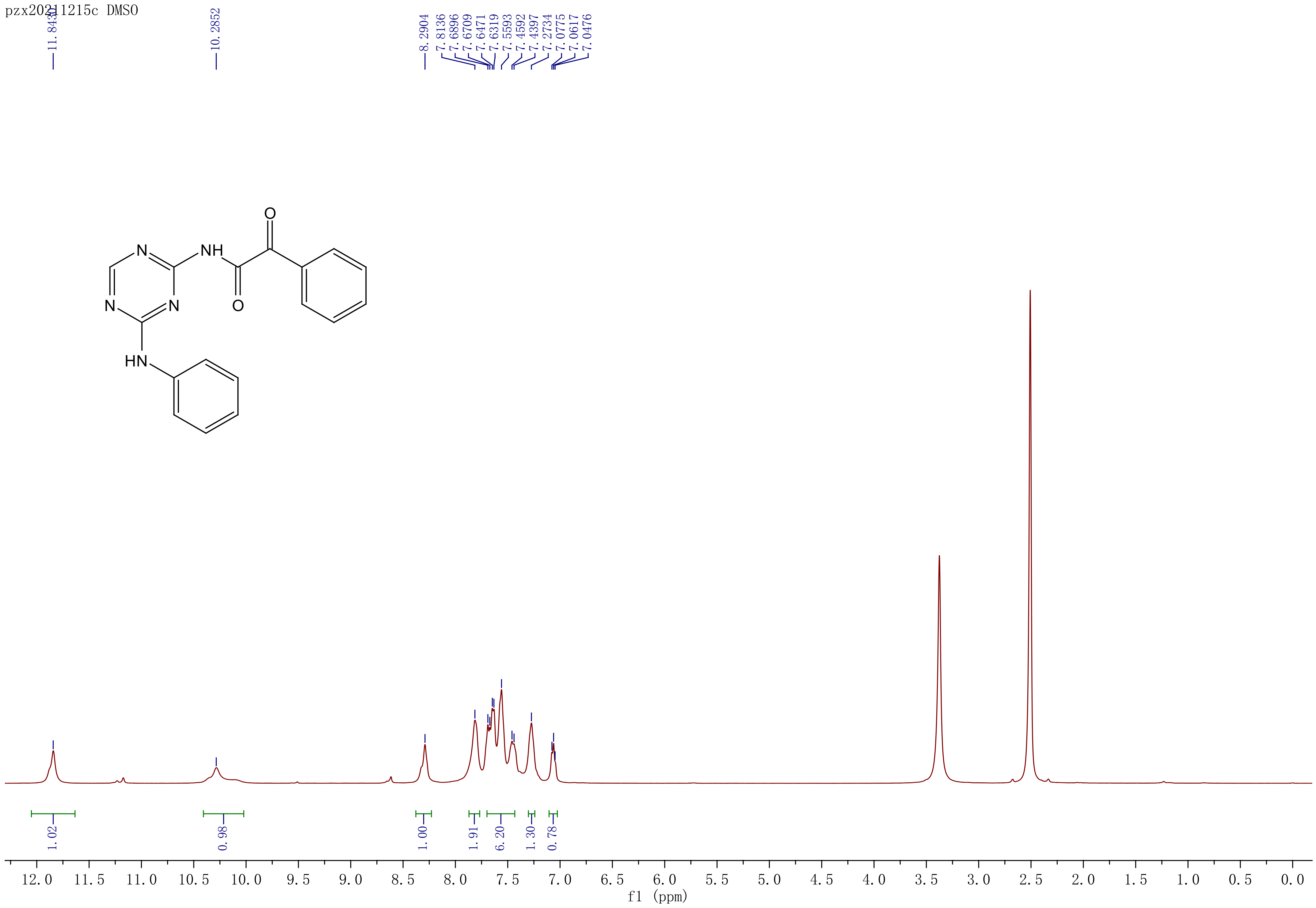
pzx20211215c DMSO
11.843
10.2852
8.2904
7.8136
7.6896
7.6709
7.6471
7.6319
7.5593
7.4592
7.4397
7.2734
7.0775
7.0617
7.0476
N
NH
O
O
N
N
HN
1.02
0.98
1.00
1.91
6.20
1.30
0.78
12.0 11.5 11.0 10.5 10.0 9.5 9.0 8.5 8.0 7.5 7.0 6.5 6.0 5.5 5.0 4.5 4.0 3.5 3.0 2.5 2.0 1.5 1.0 0.5 0.0
f1 (ppm)

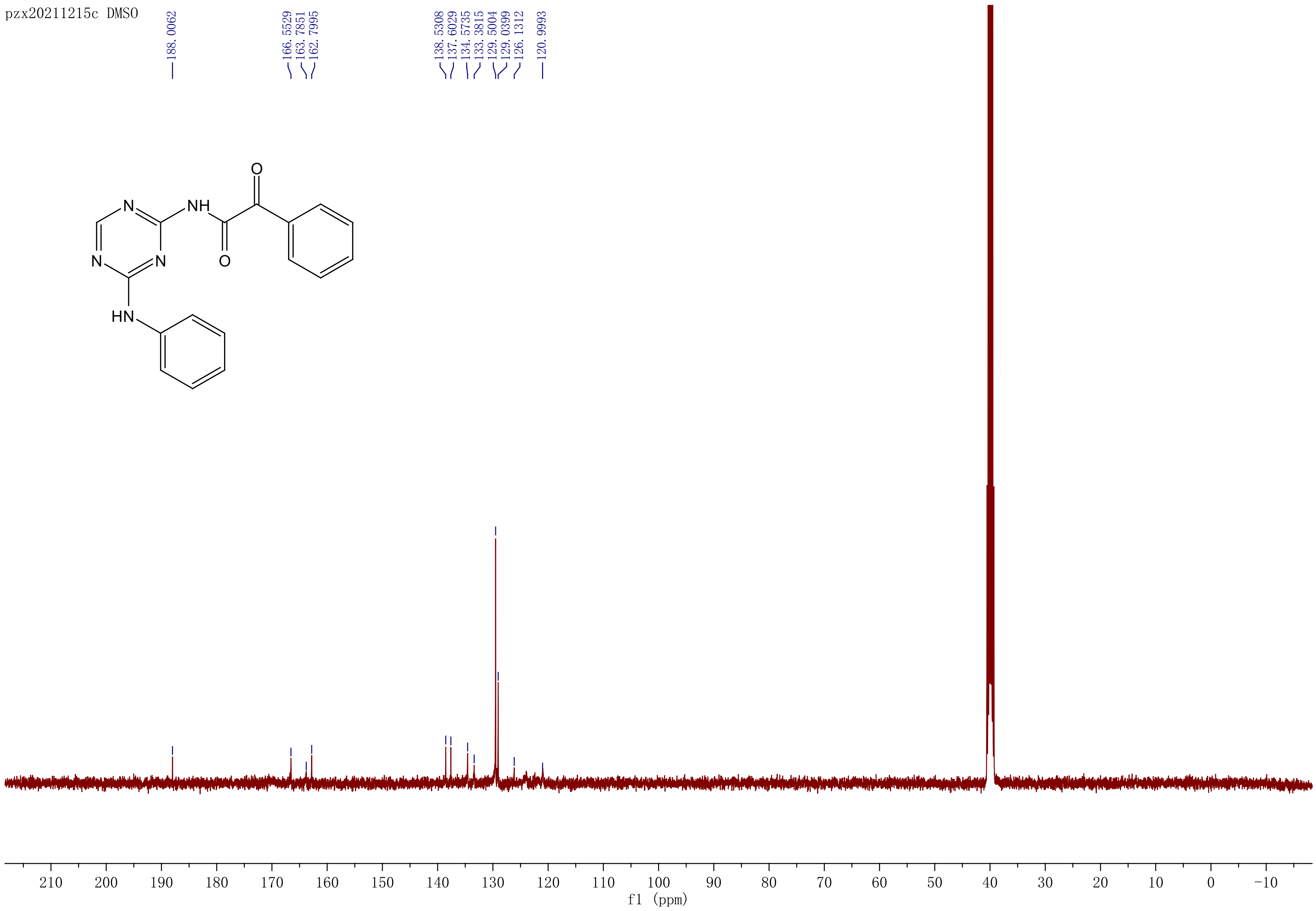

pzx20211215c DMSO
188.0062
166.5529
163.7851
162.7995
138.5308
137.6029
134.5735
133.3815
129.5004
129.0399
126.1312
120.9993
N
NH
O
O
N
N
HN
210
200
190
180
170
160
150
140
130
120
110
100
90
80
70
60
50
40
30
20
10
0
−10
f1 (ppm)

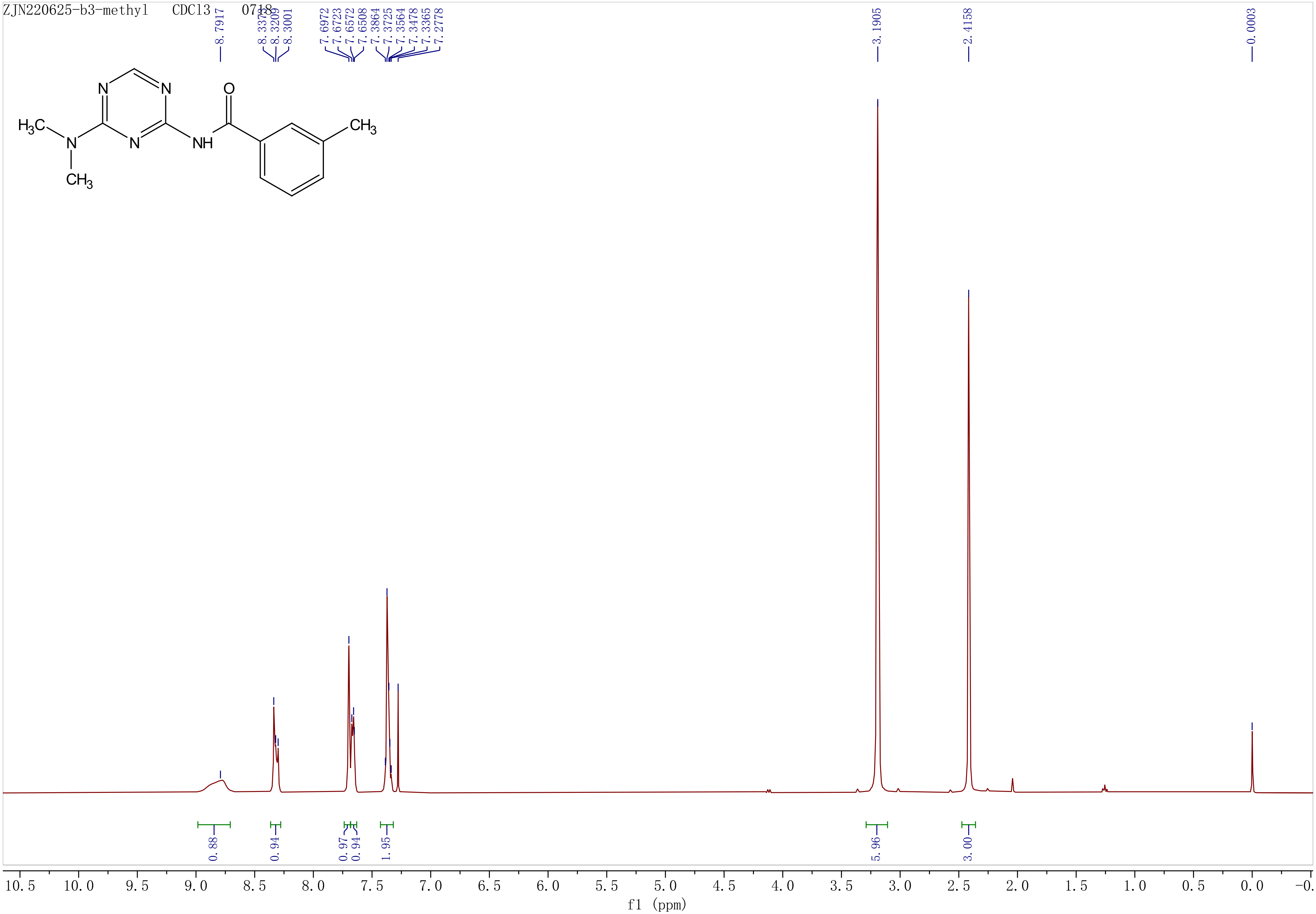

ZJN220625-b3-methyl CDCl3 0718
8.7917
8.3373
8.3209
8.3001
7.6972
7.6723
7.6572
7.6508
7.3864
7.3725
7.3564
7.3478
7.3365
7.2778
3.1905
2.4158
0.0003
N
N
O
H3C
N
N
NH
CH3
CH3
0.88
0.94
0.97
0.94
1.95
5.96
3.00
10.5 10.0 9.5 9.0 8.5 8.0 7.5 7.0 6.5 6.0 5.5 5.0 4.5 4.0 3.5 3.0 2.5 2.0 1.5 1.0 0.5 0.0 -0.
f1 (ppm)

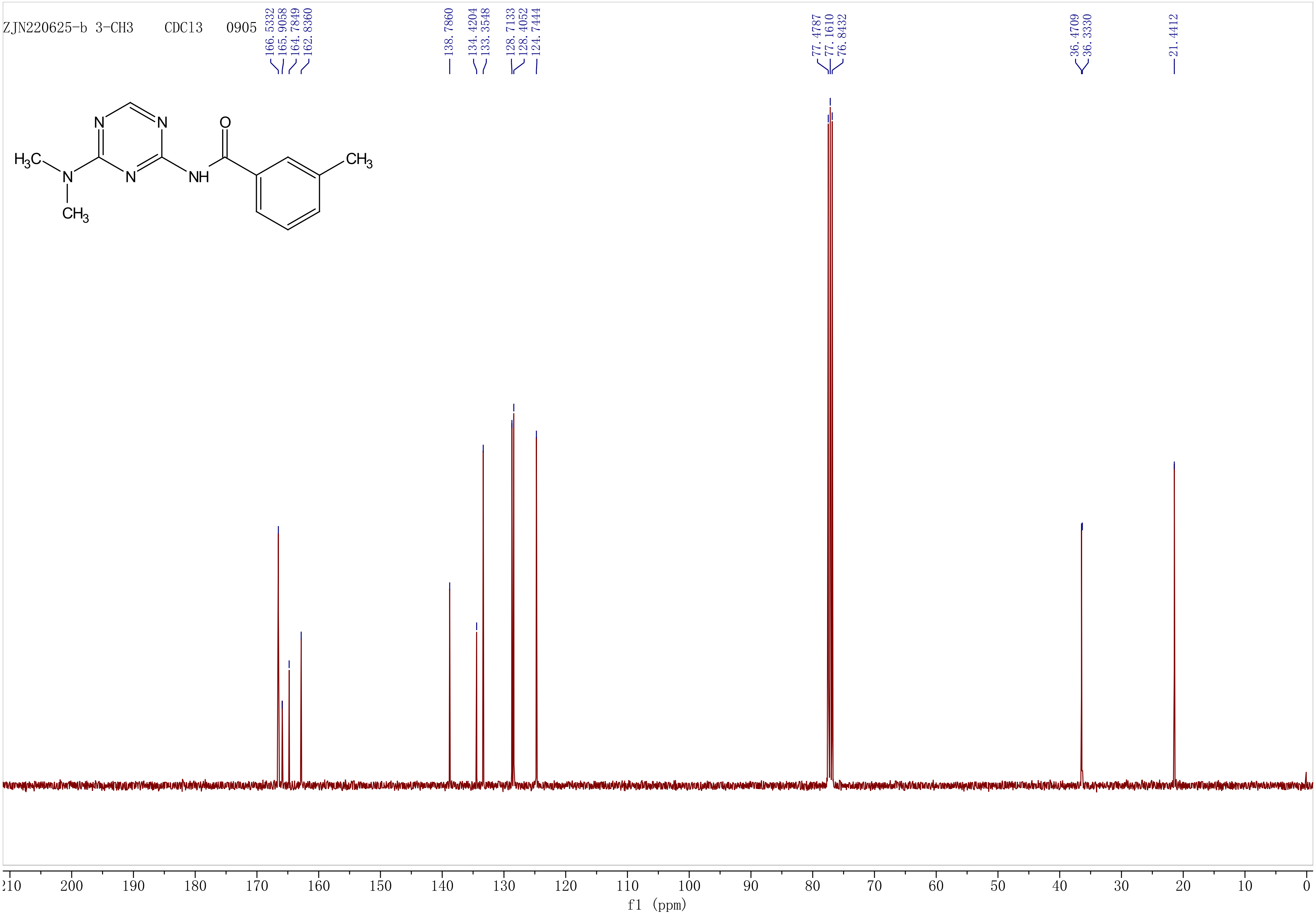
ZJN220625-b 3-CH3 CDCl3 0905
166.5332
165.9058
164.7849
162.8360
138.7860
134.4204
133.3548
128.7133
128.4052
124.7444
77.4787
77.1610
76.8432
36.4709
36.3330
21.4412
H3C
N
CH3
N
N
N
NH
O
CH3
210
200
190
180
170
160
150
140
130
120
110
100
90
80
70
60
50
40
30
20
10
0
f1 (ppm)

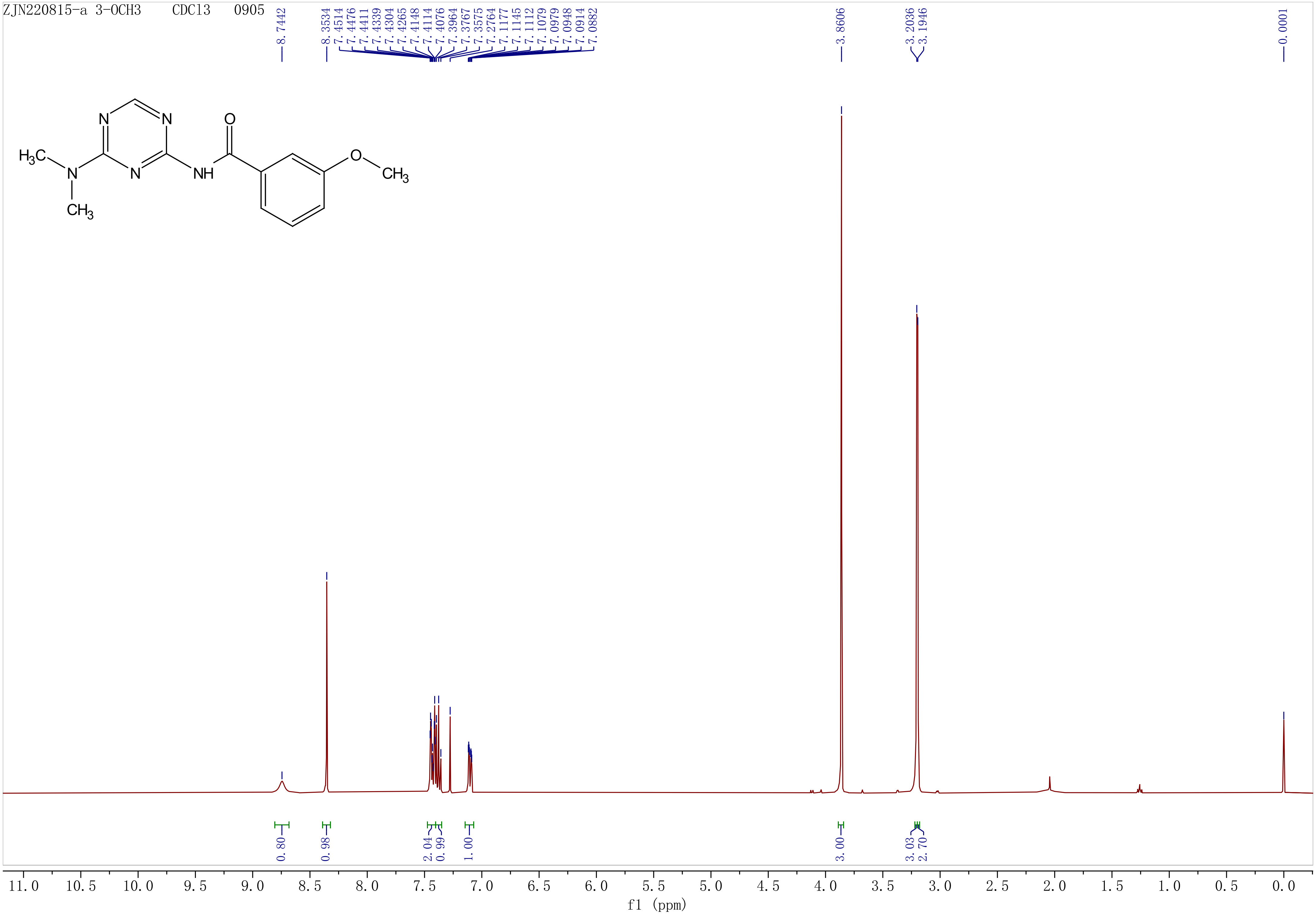

ZJN220815-a 3-OCH3 CDCl3 0905
8.7442
8.3534
7.4514
7.4476
7.4411
7.4339
7.4304
7.4265
7.4148
7.4114
7.4076
7.3964
7.3767
7.3575
7.2764
7.1177
7.1145
7.1112
7.1079
7.0979
7.0948
7.0914
7.0882
3.8606
3.2036
3.1946
0.0001
H3C
N
CH3
NH
O
CH3
0.80
0.98
2.04
0.99
1.00
3.00
3.03
2.70
11.0 10.5 10.0 9.5 9.0 8.5 8.0 7.5 7.0 6.5 6.0 5.5 5.0 4.5 4.0 3.5 3.0 2.5 2.0 1.5 1.0 0.5 0.0
f1 (ppm)

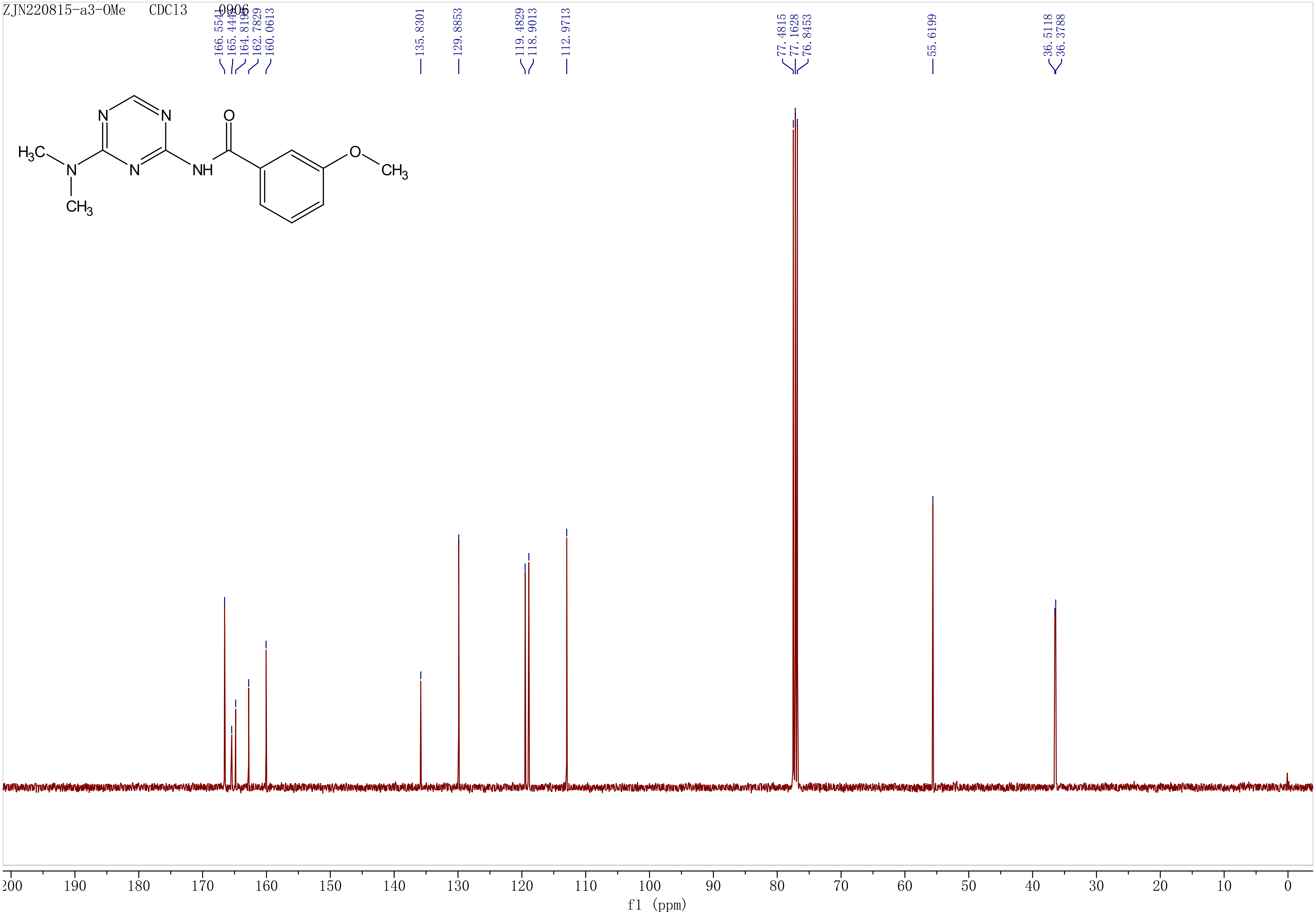
ZJN220815-a3-OMe CDCl3 0906
166.5541
165.4449
164.8193
162.7829
160.0613
135.8301
129.8853
119.4829
118.9013
112.9713
77.4815
77.1628
76.8453
55.6199
36.5118
36.3788
f1 (ppm)

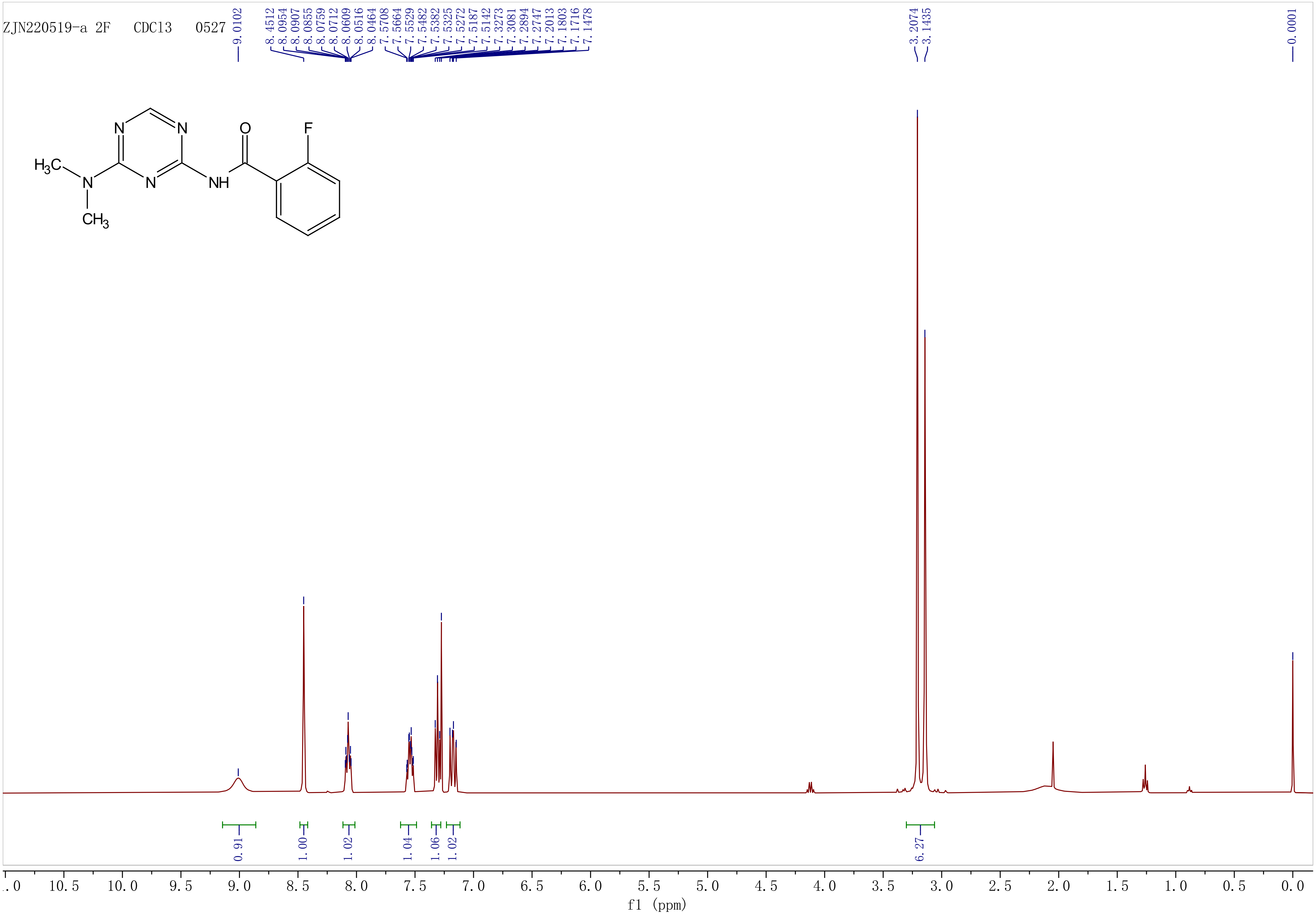
ZJN220519-a 2F CDCl3 0527
9.0102
8.4512
8.0954
8.0907
8.0855
8.0759
8.0712
8.0609
8.0516
8.0464
7.5708
7.5664
7.5529
7.5482
7.5382
7.5325
7.5272
7.5187
7.5142
7.3273
7.3081
7.2894
7.2747
7.2013
7.1803
7.1716
7.1478
3.2074
3.1435
0.0001
N
N
N
H3C
CH3
N
NH
O
F
0.91
1.00
1.02
1.04
1.06
1.02
6.27
.0
10.5
10.0
9.5
9.0
8.5
8.0
7.5
7.0
6.5
6.0
5.5
5.0
4.5
4.0
3.5
3.0
2.5
2.0
1.5
1.0
0.5
0.0
f1 (ppm)

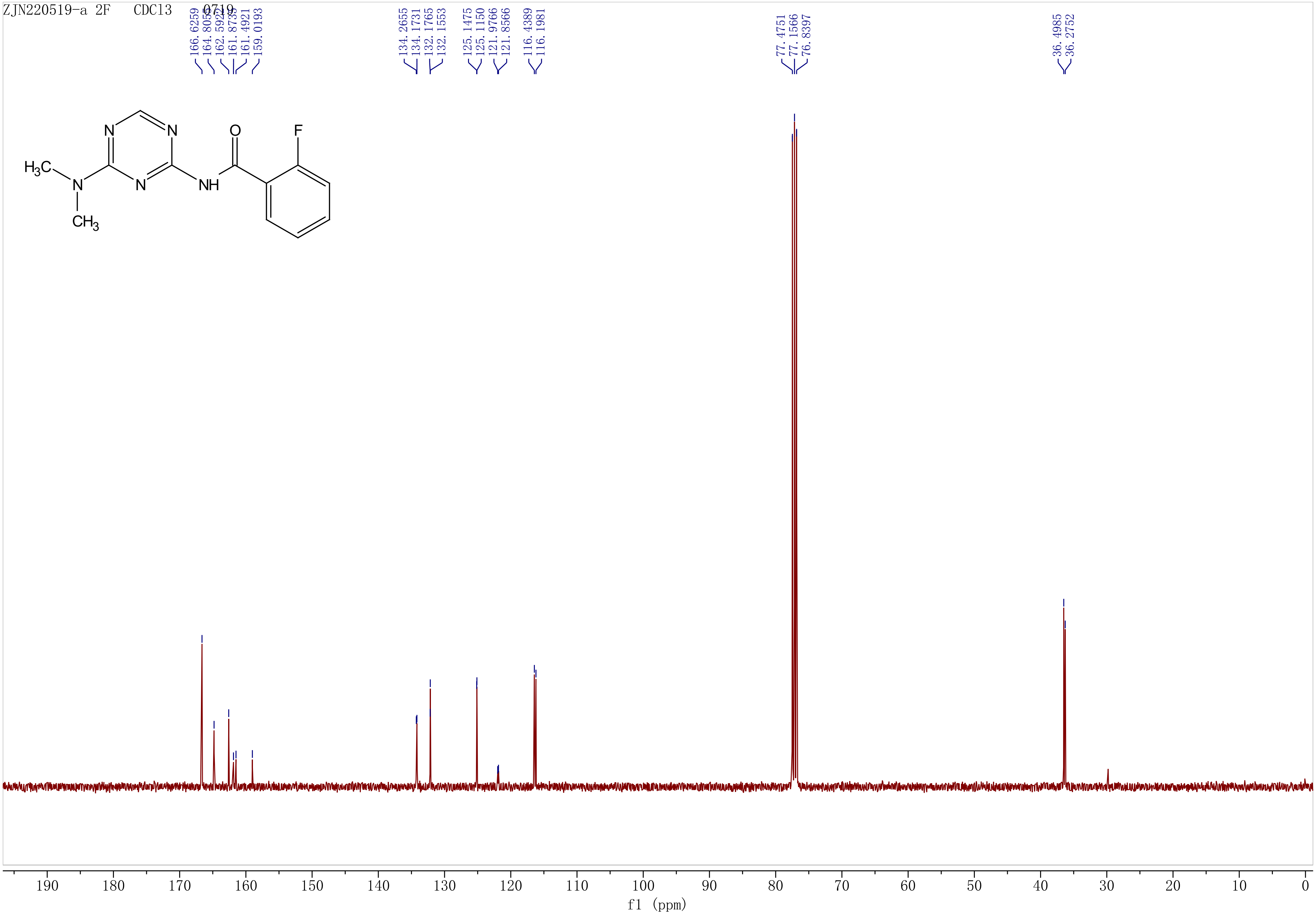
ZJN220519-a 2F CDCl3 0719
166.6259
164.8055
162.5923
161.8735
161.4921
159.0193
134.2655
134.1731
132.1765
132.1553
125.1475
125.1150
121.9766
121.8566
116.4389
116.1981
77.4751
77.1566
76.8397
36.4985
36.2752
N
N
N
O
F
H3C
N
NH
CH3
190 180 170 160 150 140 130 120 110 100 90 80 70 60 50 40 30 20 10 0
f1 (ppm)

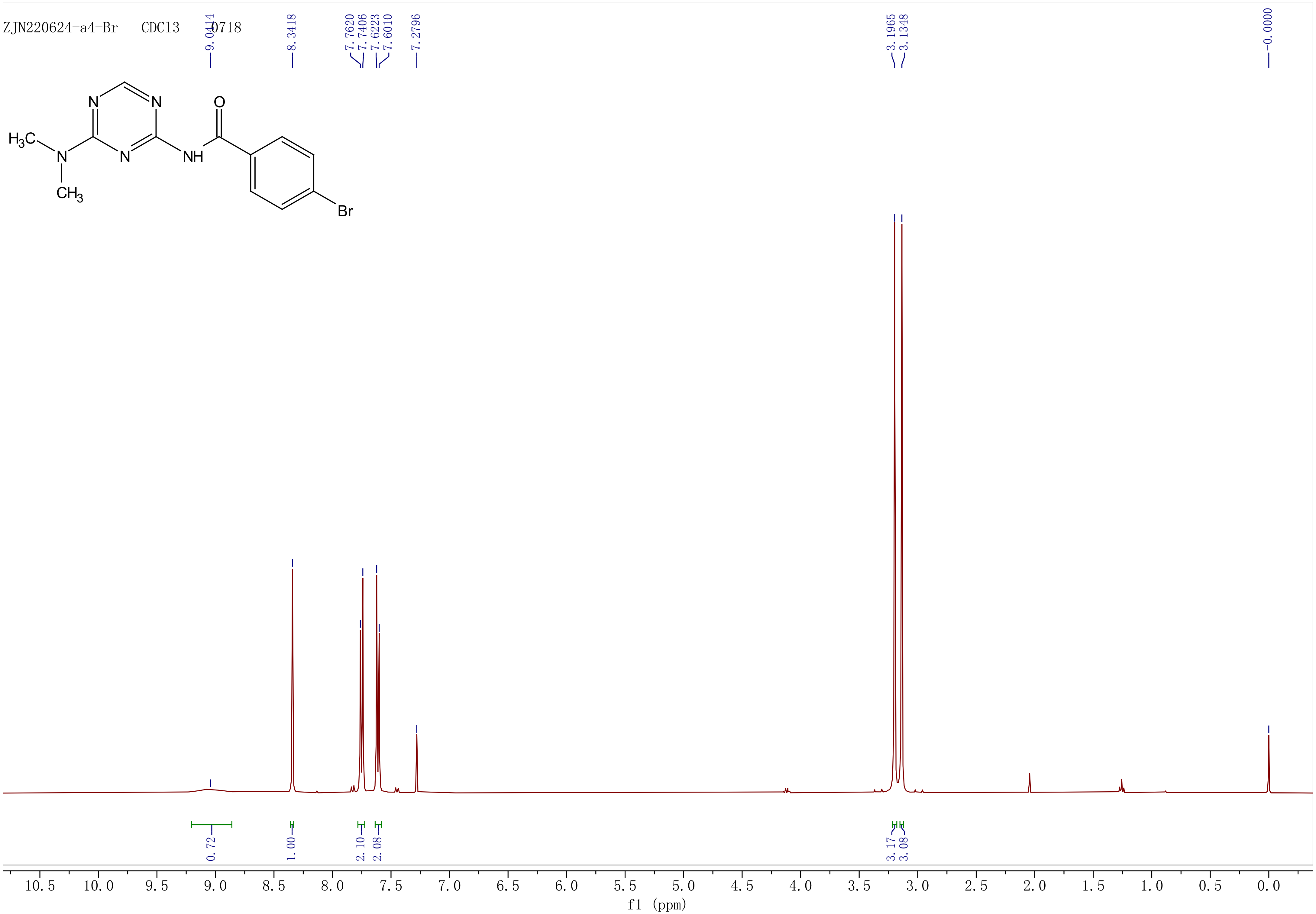

ZJN220624-a4-Br CDCl3 0718
9.0414
8.3418
7.7620
7.7406
7.6223
7.6010
7.2796
3.1965
3.1348
0.0000
0.72
1.00
2.10
2.08
3.17
3.08
f1 (ppm)

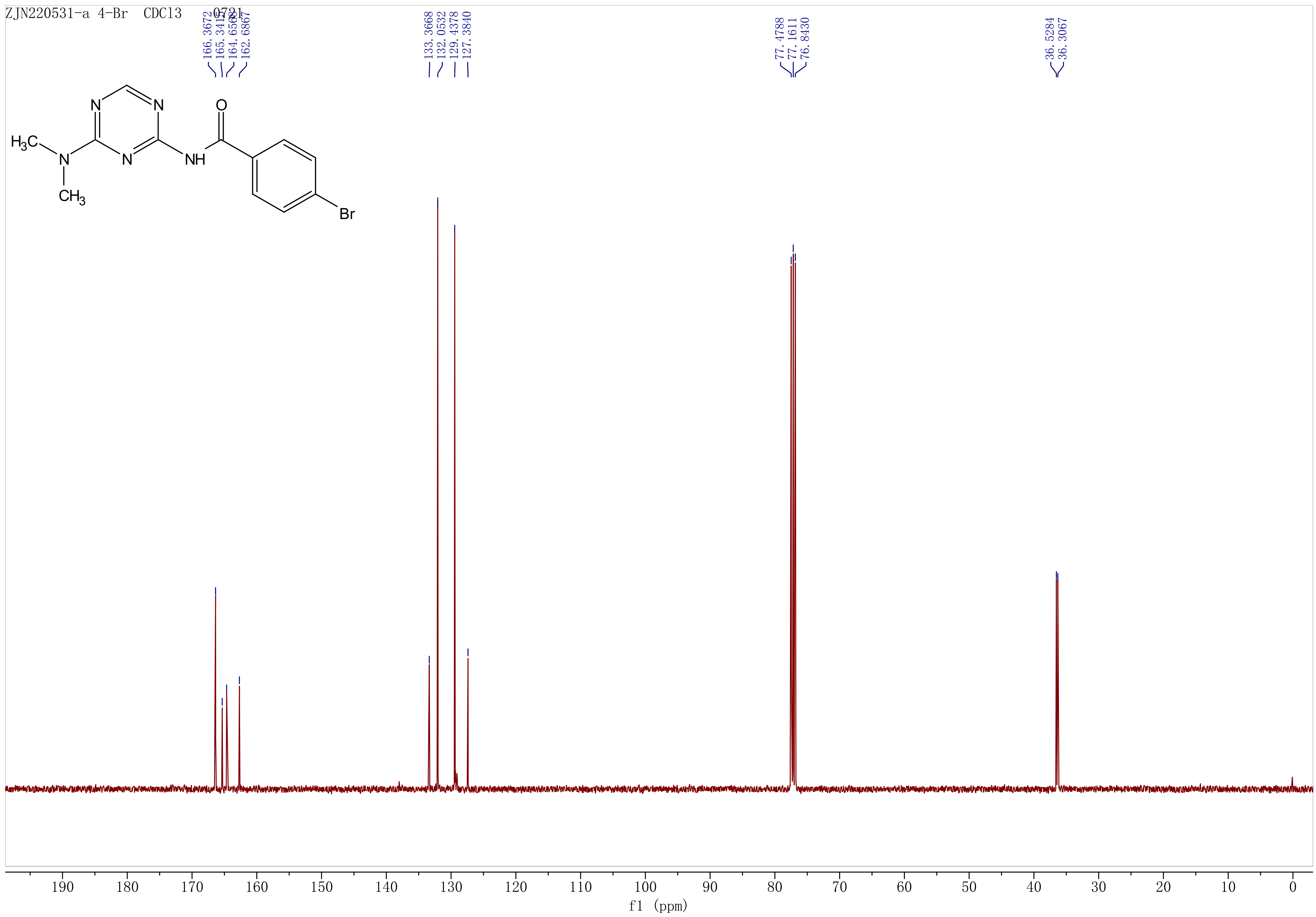

ZJN220531-a 4-Br CDCl3 0721
166.3672
165.3415
164.6561
162.6867
133.3668
132.0532
129.4378
127.3840
77.4788
77.1611
76.8430
36.5284
36.3067
H3C
N
CH3
N
N
N
NH
O
Br
190
180
170
160
150
140
130
120
110
100
90
80
70
60
50
40
30
20
10
0
f1 (ppm)

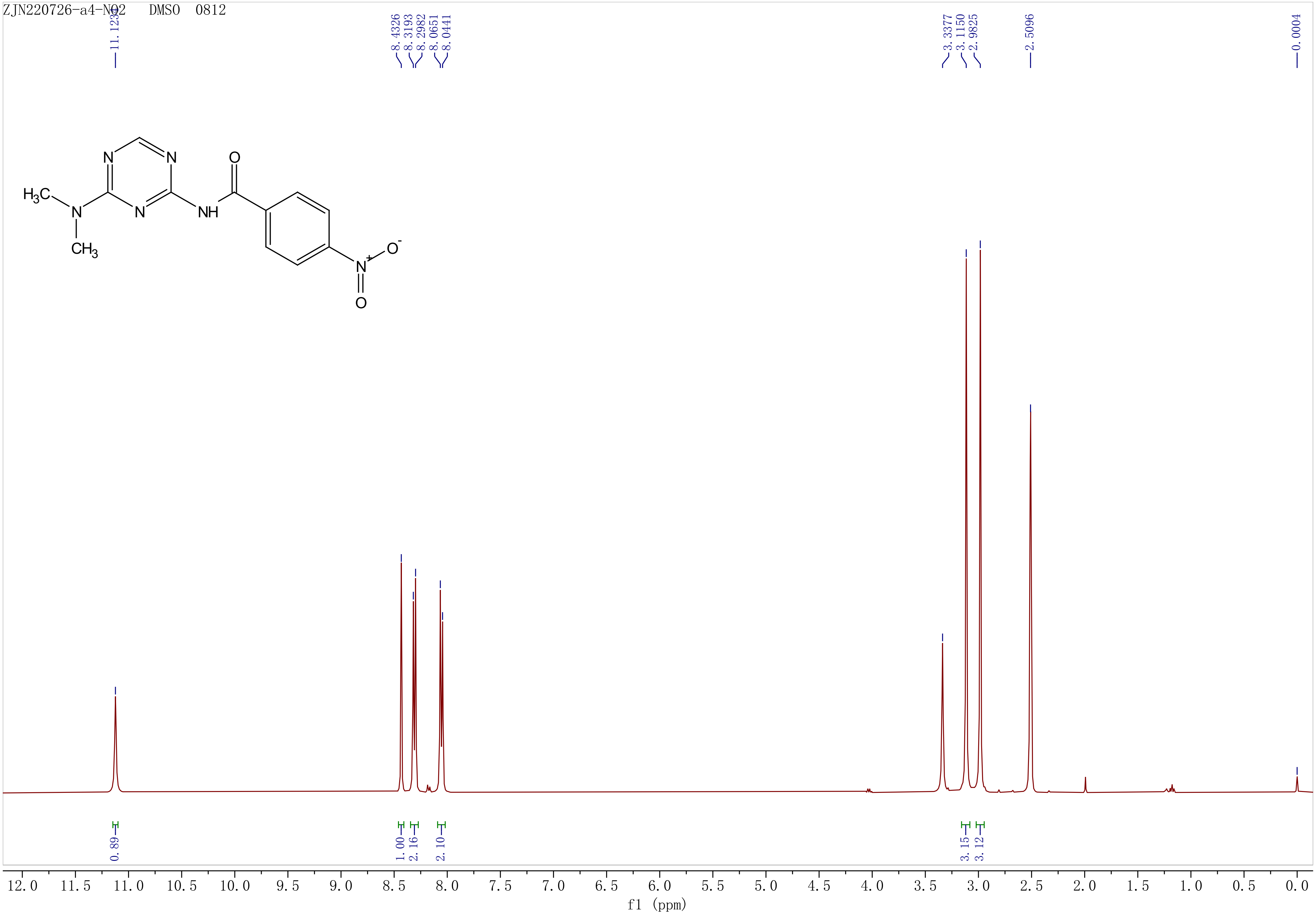
ZJN220726-a4-NO2 DMSO 0812
11.1234
8.4326
8.3193
8.2982
8.0651
8.0441
3.3377
3.1150
2.9825
2.5096
0.0004
N
N
N
O
H_3C
N
NH
CH_3
O^-
N^+
O
0.89
1.00
2.16
2.10
3.15
3.12
12.0 11.5 11.0 10.5 10.0 9.5 9.0 8.5 8.0 7.5 7.0 6.5 6.0 5.5 5.0 4.5 4.0 3.5 3.0 2.5 2.0 1.5 1.0 0.5 0.0
f1 (ppm)

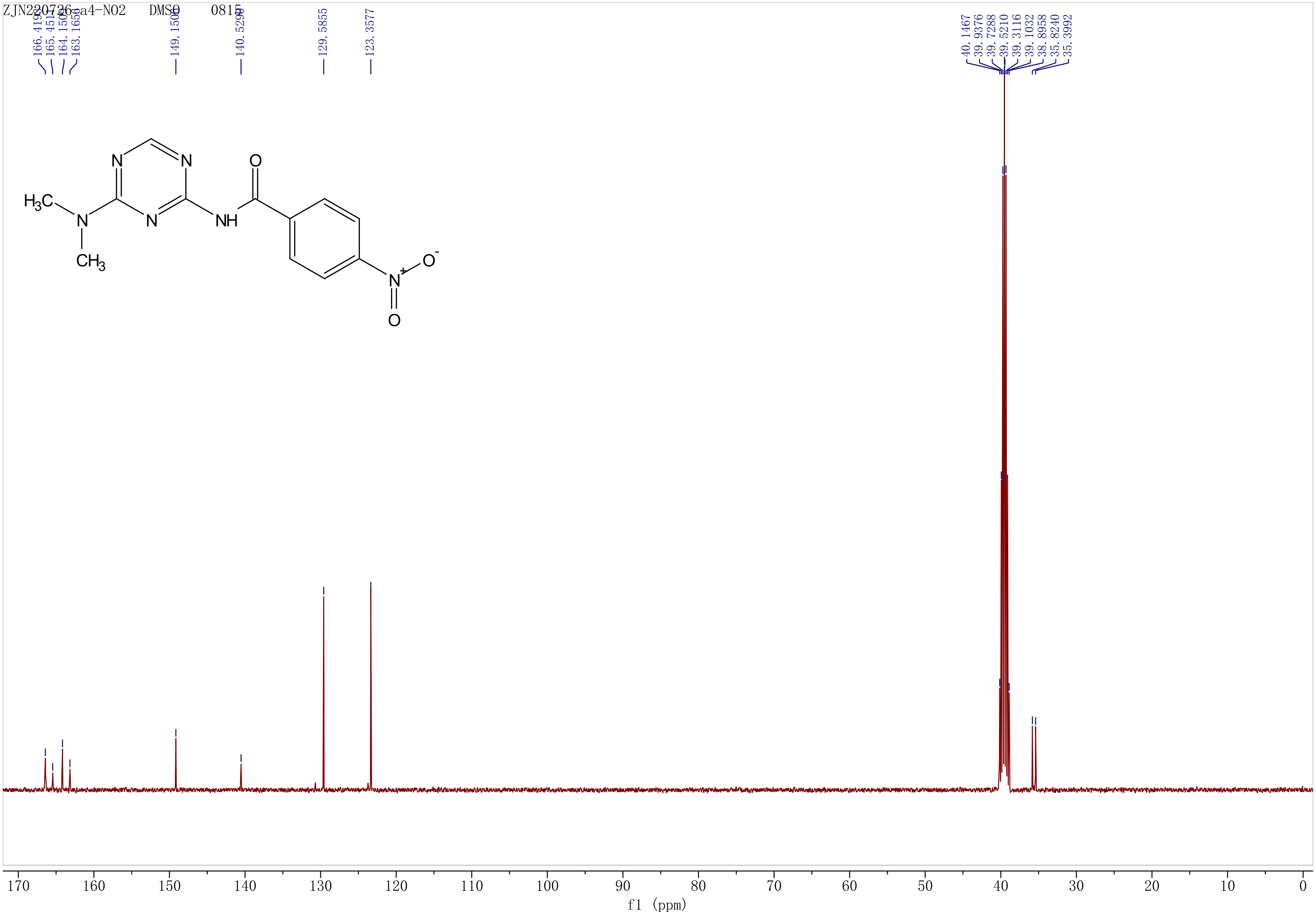
ZJN220726-a4-NO2 DMSO 0815
129.5855
123.3577
40.1467
39.9376
39.7288
39.5210
39.3116
39.1032
38.8958
35.8240
35.3992
f1 (ppm)

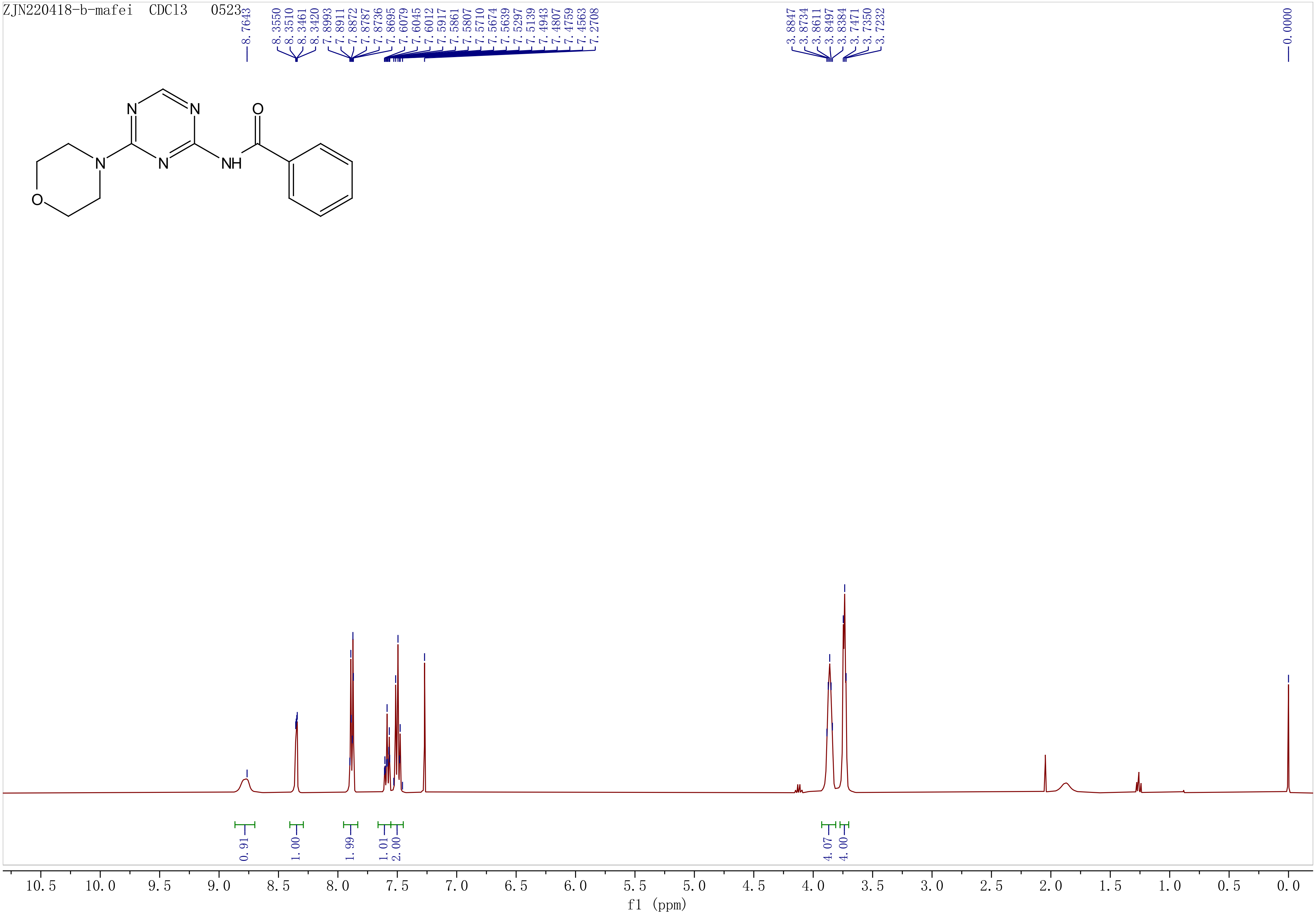
ZJN220418-b-mafei CDCl3 0523
8.7643
8.3550
8.3510
8.3461
8.3420
7.8993
7.8911
7.8872
7.8787
7.8736
7.8695
7.6079
7.6045
7.6012
7.5917
7.5861
7.5807
7.5710
7.5674
7.5639
7.5297
7.5139
7.4943
7.4807
7.4759
7.4563
7.2708
3.8847
3.8734
3.8611
3.8497
3.8384
3.7471
3.7350
3.7232
0.0000
N
N
N
O
NH
N
O
0.91
1.00
1.99
1.01
2.00
4.07
4.00
10.5 10.0 9.5 9.0 8.5 8.0 7.5 7.0 6.5 6.0 5.5 5.0 4.5 4.0 3.5 3.0 2.5 2.0 1.5 1.0 0.5 0.0
f1 (ppm)

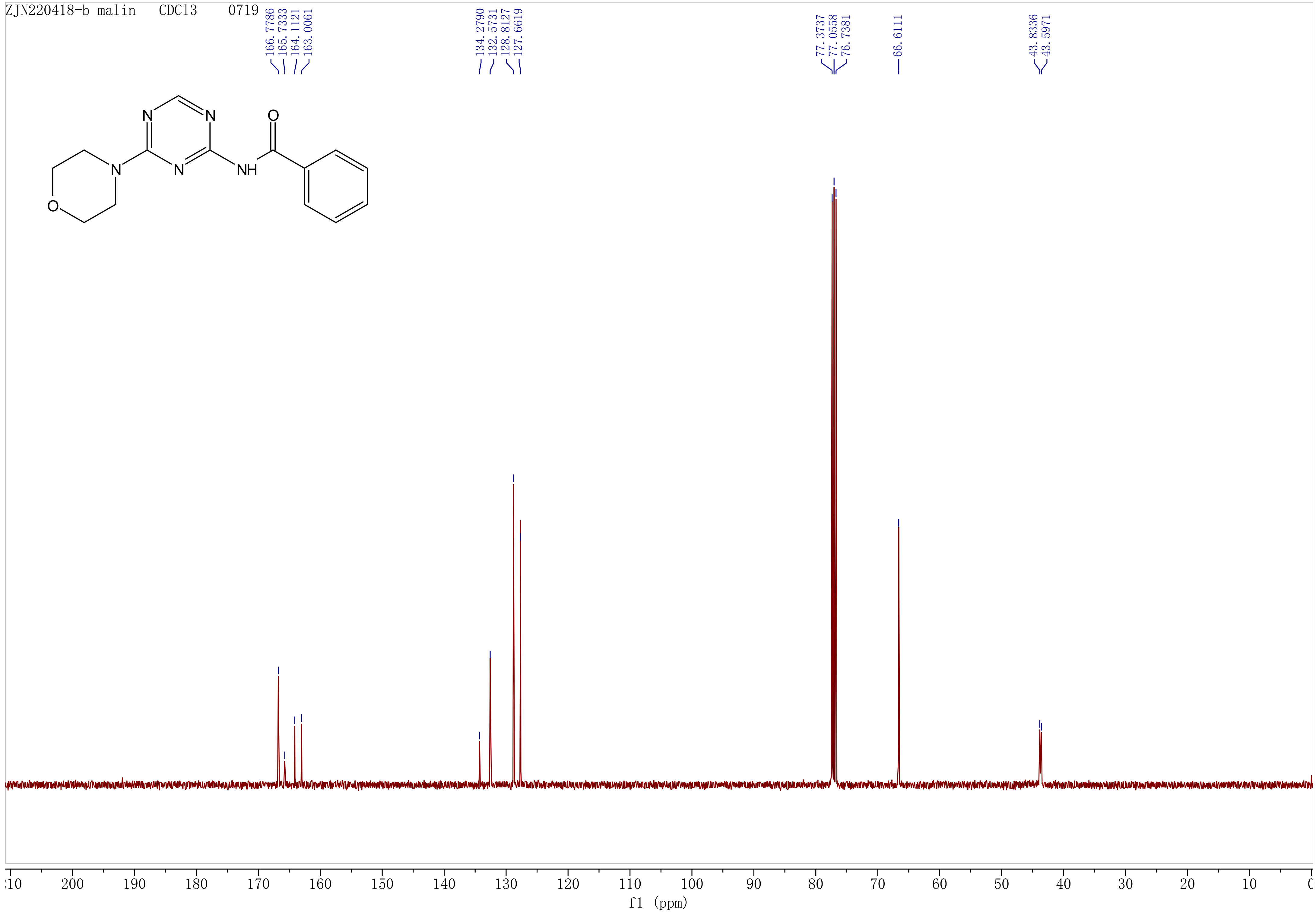

ZJN220418-b malin CDCl3 0719
166.7786
165.7333
164.1121
163.0061
134.2790
132.5731
128.8127
127.6619
77.3737
77.0558
76.7381
66.6111
43.8336
43.5971
N
N
N
O
NH
N
O
210
200
190
180
170
160
150
140
130
120
110
100
90
80
70
60
50
40
30
20
10
0
f1 (ppm)

4-F CDCl3 1018

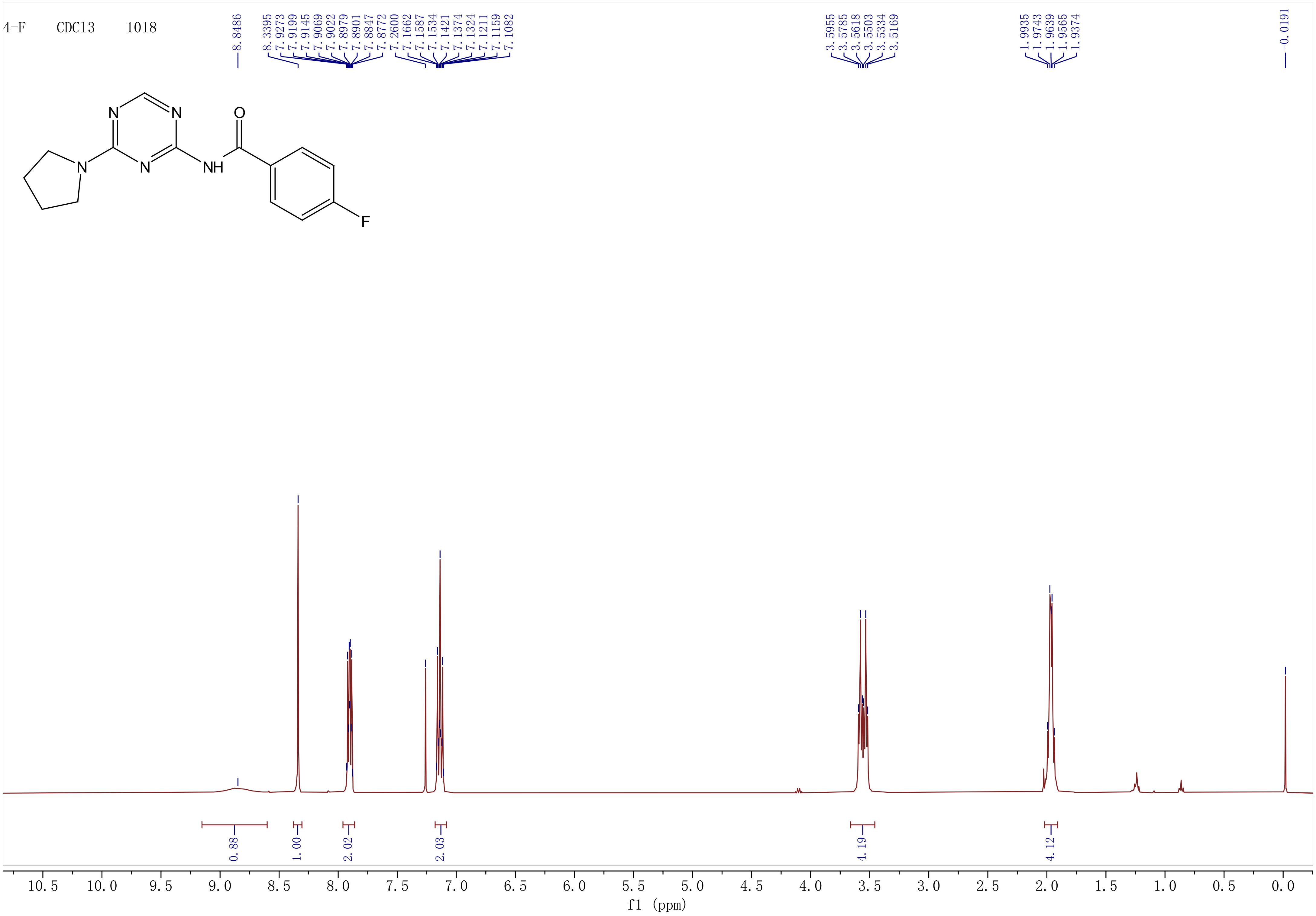

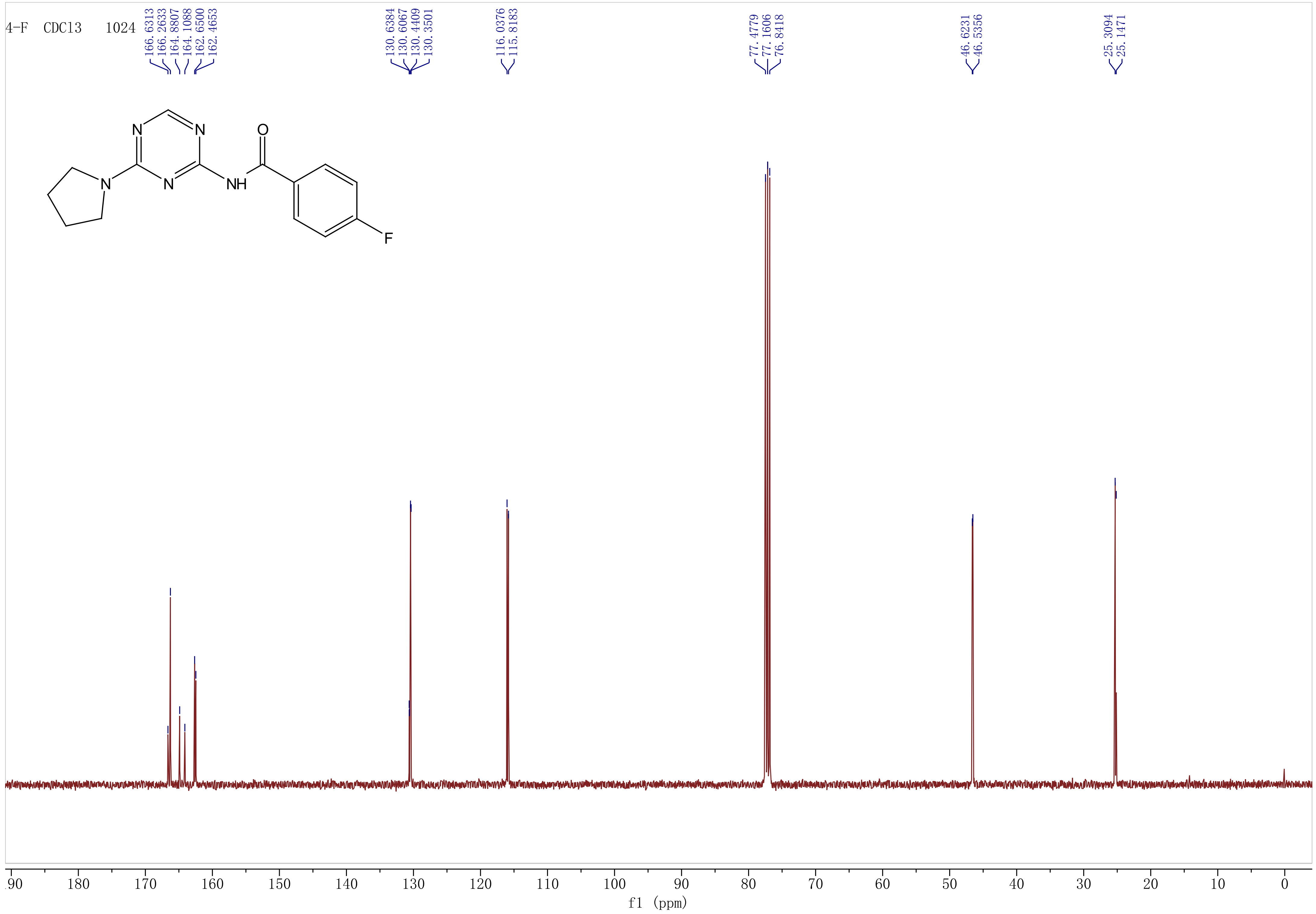
4-F CDCl3 1024
166.6313
166.2633
164.8807
164.1088
162.6500
162.4653
130.6384
130.6067
130.4409
130.3501
116.0376
115.8183
77.4779
77.1606
76.8418
46.6231
46.5356
25.3094
25.1471
N
N
N
N
NH
O
F
190
180
170
160
150
140
130
120
110
100
90
80
70
60
50
40
30
20
10
0
f1 (ppm)

HRMS for compound **3a**

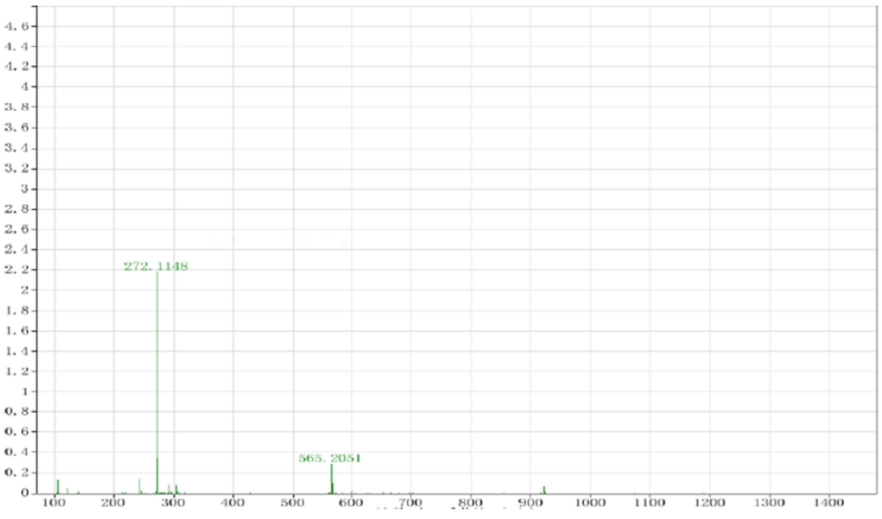

HRMS for compound **3b**

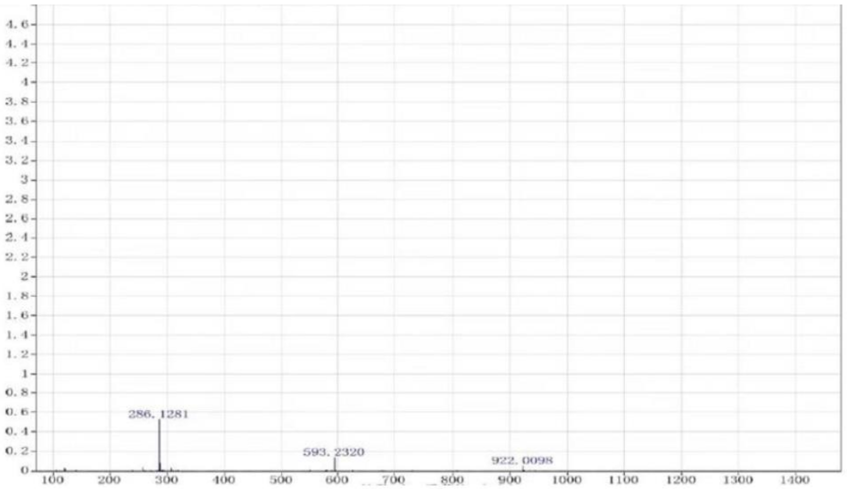

HRMS for compound **3c**

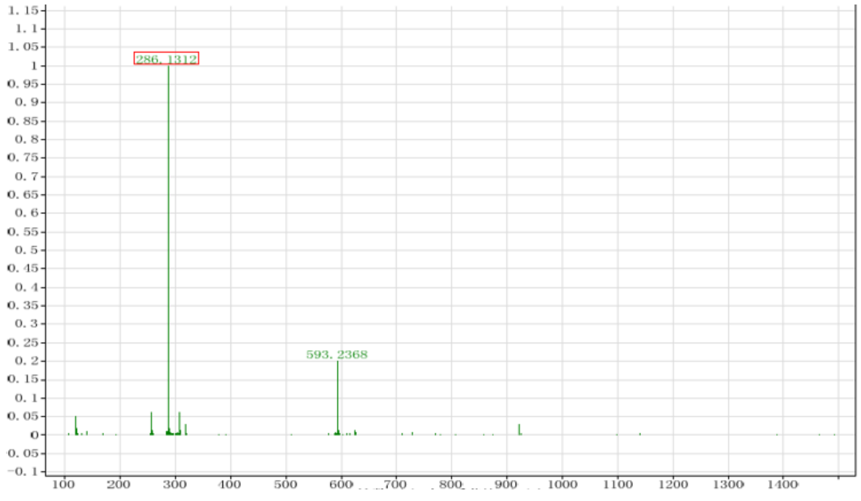

HRMS for compound **3f**

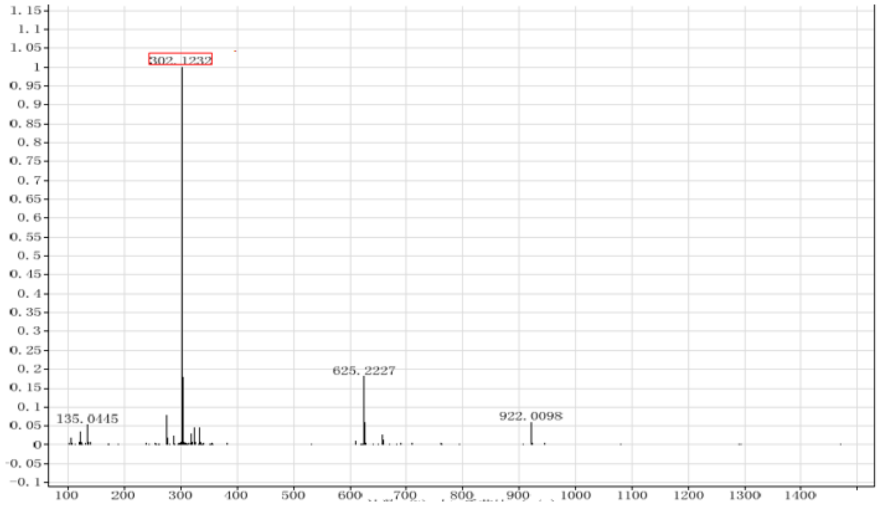

HRMS for compound **3i**

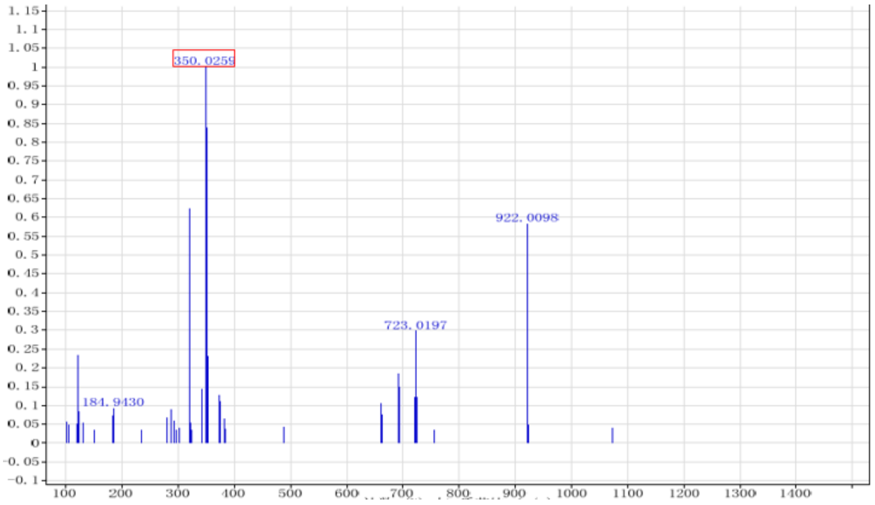

HRMS for compound **3m**

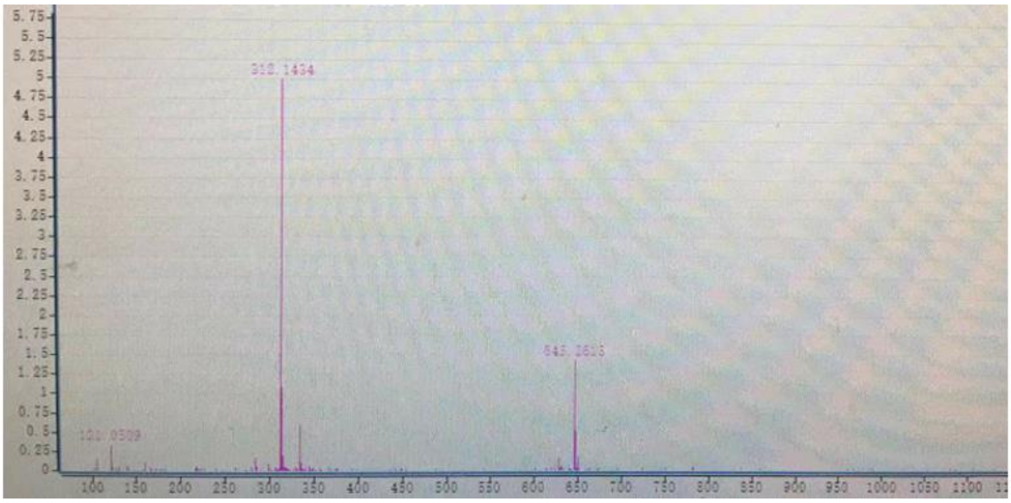

HRMS for compound **3n**

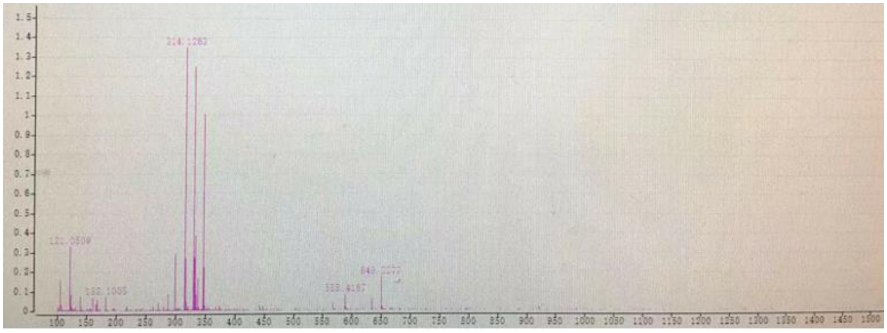

HRMS for compound **3o**

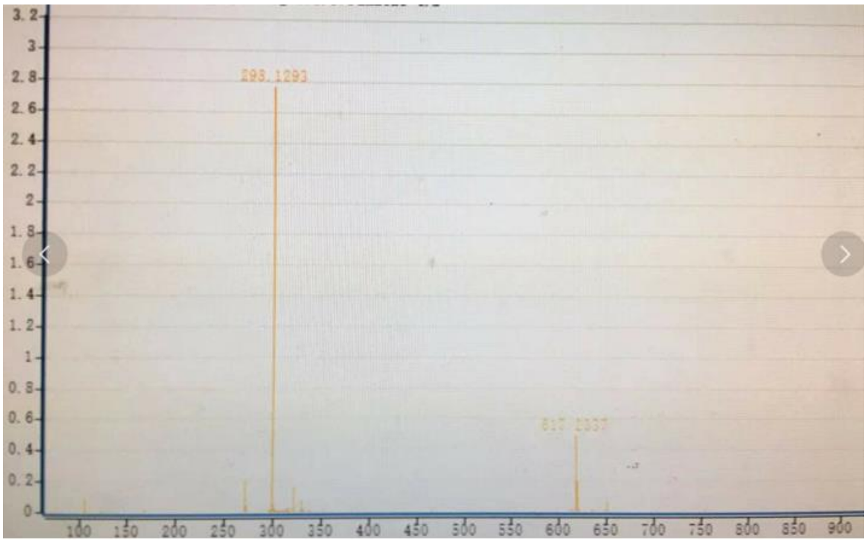

HRMS for compound **4c**

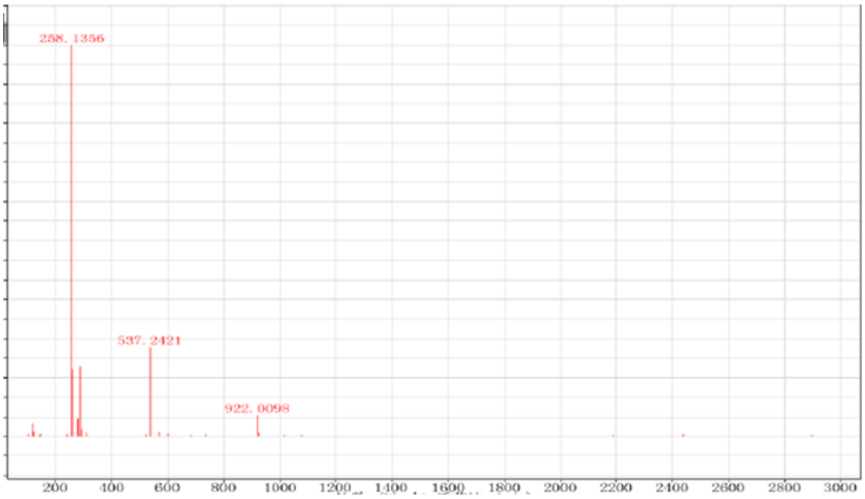

HRMS for compound **4d**

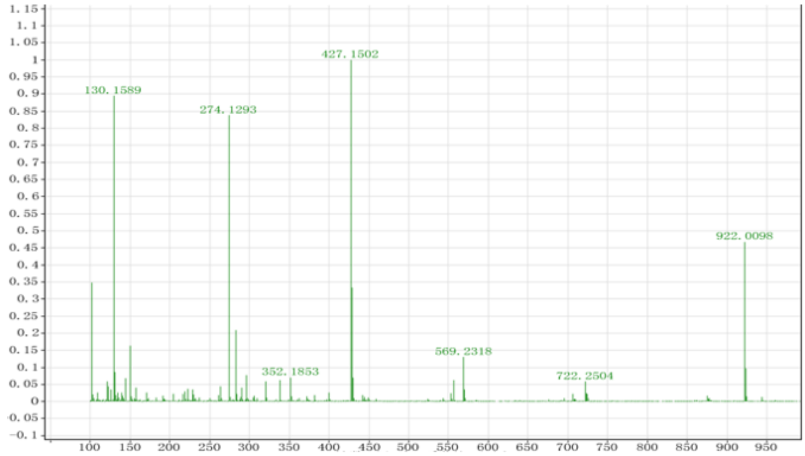

HRMS for compound **4f**

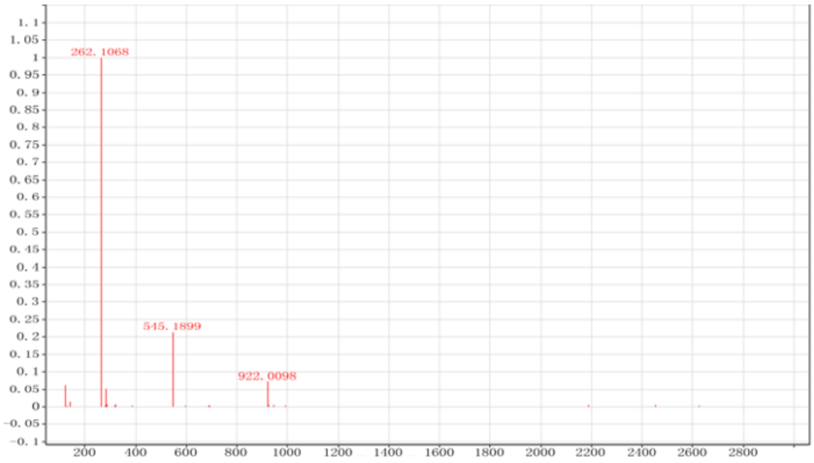

HRMS for compound **4j**

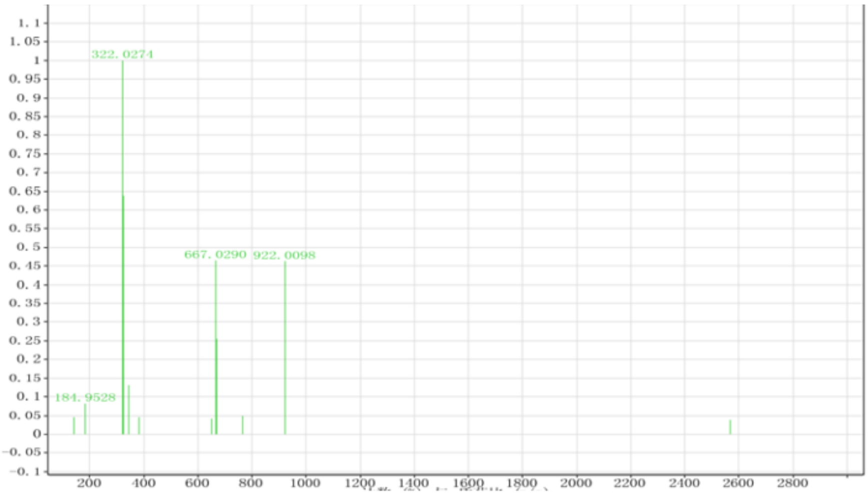

HRMS for compound **4k**

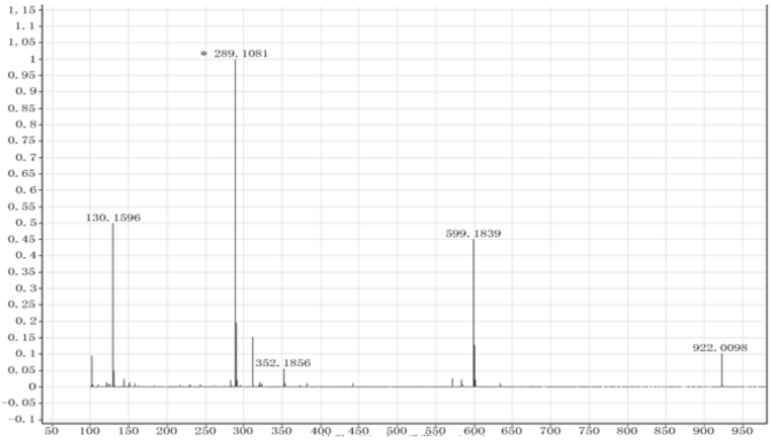

HRMS for compound **4m**

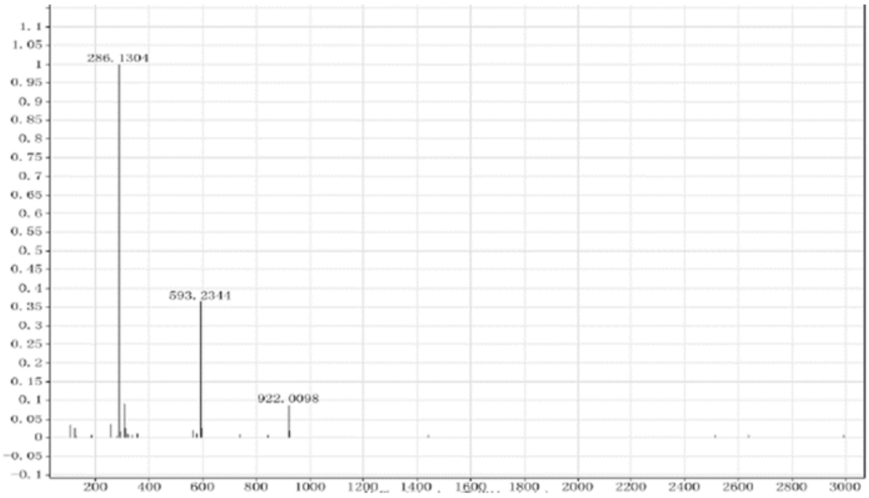

HRMS for compound **4n**

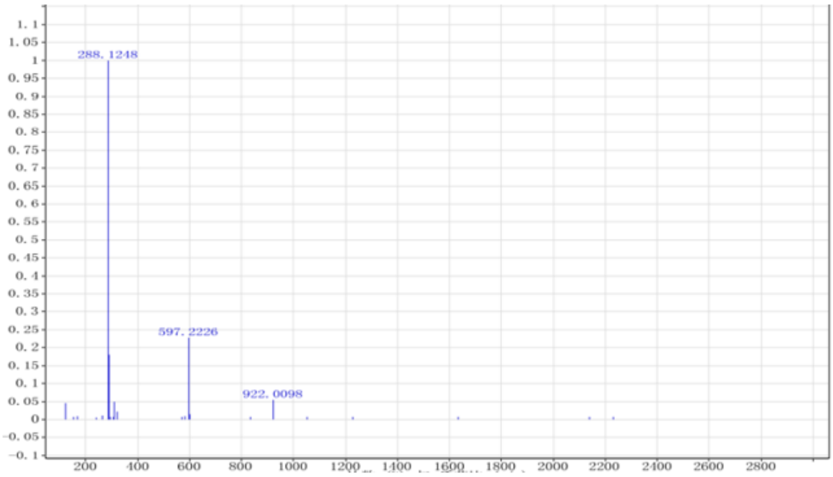

Supplement: Supplementary file 1 [file molecules-28-04338-s001.zip › molecules-2387099-supplementary.pdf]
